# A flexible kinetic assay efficiently sorts prospective biocatalysts for PET plastic subunit hydrolysis†

**DOI:** 10.1039/d2ra00612j

**Published:** 2022-03-14

**Authors:** Jessica Lusty Beech, Rita Clare, William M. Kincannon, Erika Erickson, John E. McGeehan, Gregg T. Beckham, Jennifer L. DuBois

**Affiliations:** Department of Chemistry and Biochemistry, Montana State University Bozeman MT 59717 USA jennifer.dubois1@montana.edu; Renewable Resources and Enabling Sciences Center, National Renewable Energy Laboratory Golden CO 80401 USA; Centre for Enzyme Innovation, School of Biological Sciences, Institute of Biological and Biomedical Sciences, University of Portsmouth Portsmouth PO1 2DY UK; BOTTLE Consortium Golden CO 80401 USA

## Abstract

Esterase enzymes catalyze diverse hydrolysis reactions with important biological, commercial, and biotechnological applications. For the improvement of these biocatalysts, there is a need for widely accessible, inexpensive, and adaptable activity screening assays that identify enzymes with particular substrate specificities. Natural systems for biopolymer bioconversion, and likely those designed to mimic them, depend on cocktails of enzymes, each of which specifically targets the intact material as well as water-soluble subunits of varying size. In this work, we have adapted a UV/visible assay using pH-sensitive sulfonphthalein dyes for the real-time quantification of ester hydrolysis of bis-(2-hydroxyethyl) terephthalate (BHET), a subunit of polyethylene terephthalate (PET) plastic. We applied this method to a diverse set of known PET hydrolases and commercial esterases in a microplate format. The approach identified four PET hydrolases and one commercial esterase with high levels of specificity for BHET hydrolysis. Five additional PET hydrolases and three commercial esterases, including a thermophilic enzyme, effectively hydrolyzed both BHET and its monoester product MHET (mono-(2-hydroxyethyl) terephthalate). Specific activities were discernible within one hour and reactions reached an unequivocal endpoint well within 24 hours. The results from the UV/visible method correlated well with conventional HPLC analysis of the reaction products. We examined the suitability of the method toward variable pH, temperature, enzyme preparation method, mono- and multi-ester substrate type, and level of sensitivity *versus* stringency, finding the assay to be easily adaptable to diverse screening conditions and kinetic measurements. This method offers an accurate, easily accessible, and cost-effective route towards high-throughput library screening to support the discovery, directed evolution, and protein engineering of these critical biocatalysts.

## Abstract 2

We have developed a colorimetric assay for the high throughput detection of enzymatic ester hydrolysis of PET plastic monomers, applied in a microplate format.

## Graphical abstract

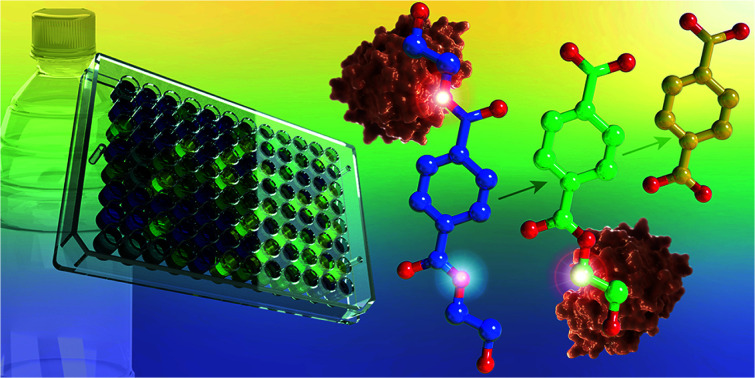

## Introduction

**Scheme 1:**
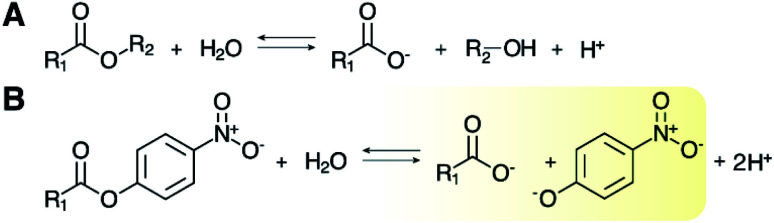
Esterase-catalyzed reaction and canonical activity assay. (A) Enzymatic hydrolysis of an ester at near neutral to alkaline pH (>6) yields an alcohol, carboxylate, and a proton (H^+^). (B) A *p*NP-ester conjugate used as an activity reporter is hydrolyzed to yield a mixture of the yellow alkaline and colorless acidic forms of *p*NP in addition to the carboxylate and H^+^. The alkaline species is shown.

Ester linkages occur in an array of natural and synthetic polymers and small molecules that serve as water-impermeable barriers, surfactants, waxes, scents, flavorings, and pharmaceuticals. Biocatalysis features prominently among green methods for selectively forming and subsequently hydrolyzing ester bonds at both laboratory- and industrial-scales.^1–5^ However, methods for monitoring esterase activity are often limited by the lack of a chromophore or fluorophore in native or desired substrates. The most commonly utilized assay, found in both published literature and commercial esterase protocols, employs alkyl-*para*-nitrophenol (*p*NP) esters (Scheme 1) that are readily hydrolyzed by a wide variety of esterases.^1^ In its alkaline form (deprotonated at the phenolic oxygen), *p*NP exhibits a yellow color and is readily detected by UV/visible spectrophotometry. UV/visible methods afford considerable advantages in accessibility, cost, and throughput over ultrahigh pressure liquid chromatography (UHPLC) methods with or without coupling to mass spectrometry (MS). Though these methods can be carried out in high throughput using robotic systems, such instrumentation is expensive and unavailable in many labs. Only a few *p*NP-linked substrates are commercially available, however, and their use sharply limits efforts focused on identifying enzymatic activity with specific substrates or substructures.

Addressing this gap, Kazlauskas and co-workers^6^ reported an assay based on detecting the proton (H^+^) released from carboxylic ester hydrolysis. The assay used free *p*NP as a colorimetric pH indicator (*p*NP–O^−^ + H^+^ ⇆ *p*NP–OH, p*K*_a_ = 7.2), where the alkaline form is yellow and the acidic form is colorless (*λ*_max_ 404 nm, *ε* = 17 800 M^−1^ cm^−1^, pH 7.2, 5 mM BES buffer).

However, while *p*NP is a well-described compound with a convenient p*K*_a_, its chromophore is relatively weak, limiting the attainable sensitivity of the assay. Moreover, the *p*NP indicator is useful only over a limited pH range. Even so, a method employing H^+^ detection, in principle, provides a readout for nearly any carboxylic ester as a hydrolysis substrate.

**Fig. 1:**
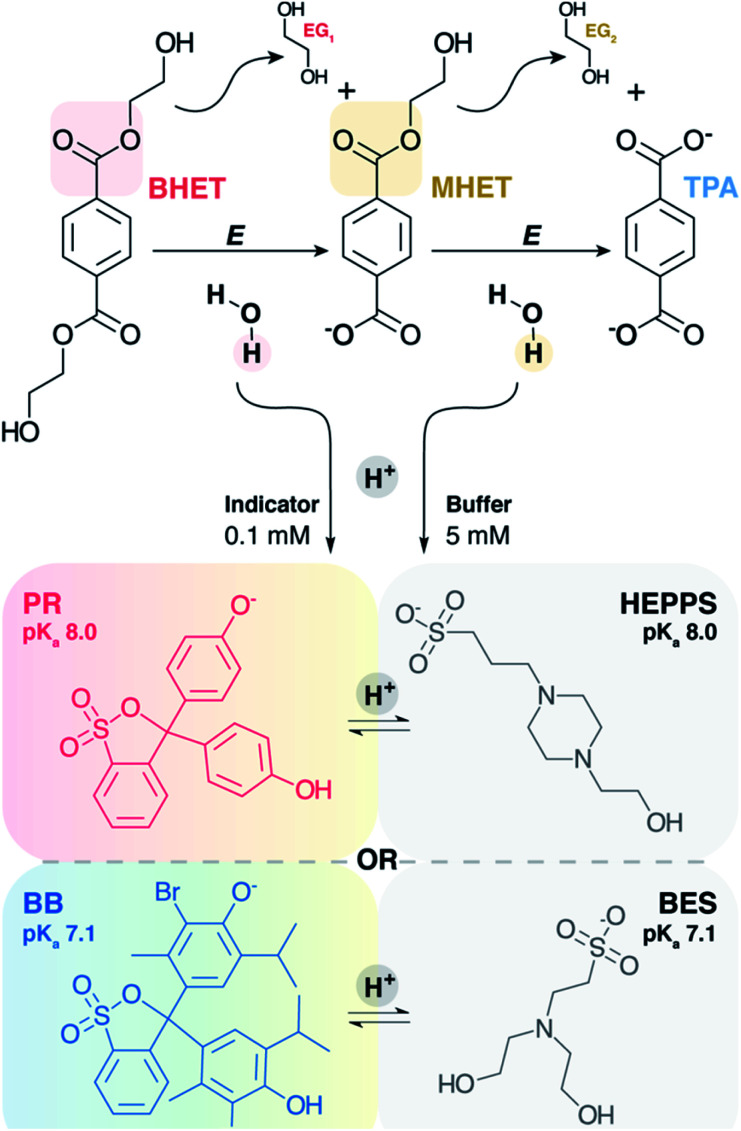
Hydrolysis of BHET esters and detection of H^+^. The first ester of BHET is enzymatically (E) hydrolyzed to ethylene glycol (EG) and mono-(2-hydroxyethyl) terephthalate (MHET), which is further hydrolyzed to EG and terephthalate (TPA). At an assay pH well above the p*K*_a_s of these products, each ester hydrolysis reaction releases nearly a full equivalent of H^+^ into solution. This binds either to a buffer or a pH sensitive dye – phenol red (PR) or bromothymol blue (BB) – having matched p*K*_a_ values. The resulting color change is detectable by UV/visible absorbance spectroscopy.

In 2018, Martinez-Martinez *et al.* adapted the Kazlauskas assay for a high throughput screen to identify the most substrate-promiscuous esterases from a group of 145 diverse enzymes.^7^ While much of the work employed *p*NP as a pH indicator, some experiments used 4,4′-(1,1-dioxido-3*H*-2,1-benzoxathiol-3-ylidene)bis-phenol, also known as phenolsulfonphthalein or phenol red (PR) (Fig. 1). Though the rationale given for the use of PR was its slightly alkaline p*K*_a_ (PR–O^−^ + H^+^ ⇆ PR–OH, p*K*_a_ = 8.0), several other of its properties recommend it for adoption in esterase assays: high absorptivity, multicolor readout, the availability of structural congeners with a broad range of p*K*_a_s (Fig. S1†), and the likely stability of its triphenolic structure under different temperature and aeration conditions.

Here, we have adapted a proton-detecting assay to identify esterases that specifically prefer bis-(2-hydroxyethyl) terephthalate (BHET) as their substrate. BHET is a small, aromatic diester that is efficiently produced from chemical glycolysis^8^ and biological depolymerization of polyethylene terephthalate (PET).^9,10^ The combined use of esterases specific for PET and mono-(2-hydroxyethyl) terephthalate (MHET) accelerates decomposition of the solid polymer.^9,10^ We expect a BHET-directed esterase to act synergistically with PET esterases in a similar fashion. Finally, BHET is also a promising precursor for metabolically engineered biocatalysis in microbial hosts, enabling the bioconversion of a plastic waste-derived molecule into valuable product(s).^8,11–15^ A potent BHET esterase (BHETase) would consequently be of significant importance for PET and/or BHET deconstruction, chemical recycling, and upcycling.

A candidate BHETase could hydrolyze BHET to the monoester MHET, and ethylene glycol (EG); alternatively, MHET may be further hydrolyzed to terephthalate (TPA) and EG as the final products (Fig. 1). Some characterized PET hydrolases have been shown to fully hydrolyze PET to its TPA and EG monomers,^16,17^ so that TPA accumulation can be used to measure the extent of PET conversion. Esterases with known PET activity consequently are of interest for screening with BHET and for exploring the structure–function relationships that distinguish hydrolysis of BHET and MHET. Many esterases are also intrinsically substrate-promiscuous, promoting their use in biotechnological applications; thus, some commercially available esterases may be active on BHET. The set of enzymes screened here included nine esterases with previously described reactivity against solid PET or PET analogs (enzymes 1–9, Tables 1 and S1†) and nine diverse, widely used commercial esterases and lipases (enzymes 10–18, Tables 1 and S2;† here simply referred to as “esterases”). The assay was examined for different preparation formats (pure, semi-pure, and freeze-dried cells) of the enzymes, reaction mixture components, and conditions, and correlated with conventional high performance liquid chromatography (HPLC) analyses of reactants and products in tandem.

**Table 1:** Enzymes used in this work

Enzyme number	Species/tissue of origin	Accession/sigma catalog number
1	*Thermobifida fusca* DSM4379 3	WP_011291330.1 (ref. 16, 17 and 29–34)
2	*Ideonella sakaiensis* 201-F6	GAP38373.1 (ref. 9 and 52)
3	Uncultured bacterium	AEV21261.1 (ref. 17, 35 and 36)
4	*Thermobifida fusca* NTU22	ADM47605.1 (ref. 37)
5	*Thermobifida fusca* DSM4434 2	ADV92528.1 (ref. 38)
6	*Thermobifida cellulosilytica* DSM4453 5	ADV92527.1 (ref. 38)
7	*Thermobifida alba* DSM4318 5	ADV92525.1 (ref. 39)
8	*Thermobifida alba*	BAI99230.2 (ref. 40 and 49)
9	*Thermobifida fusca*	ALF04778.1 (ref. 9, 16, 17 and 29–34)
10	*Geobacillus stearothermophilus*	79302-10MG^28^
		OAO77298.1
11	*Bacillus subtilis*	96667-10MG^41^
		WP_169507057
12	*Aspergillus oryzae*	62285-100MG-F
13	*Candida antarctica* (CalA)	62287-50MG-F
		W3VKA4.2
14	*Candida antarctica* (CalB)	62288-50MG-F
		WP_169507057
15	*Rhizopus oryzae*	79208-100mg-F
16	*Aspergillus oryzae*	L3295-50ML
17	*Pseudomonas* sp.	62335-10MG
18	Bovine pancreas	P8913-5MG

## Experimental

### Reagents and stocks

Chemicals and commercial enzymes were obtained from MilliporeSigma at the highest available purity grade and used as supplied unless otherwise specified. Buffers were made in Milli-Q (MilliporeSigma) purified deionized water. Commercial enzymes (Tables 1 and S2†) were resuspended from lyophilized powders into 5 mM BES (pH 7.1) or HEPPS buffer (pH 8.0). The concentrations of protein stocks were determined *via* Bradford assay.^18^

Enzymes 1–9 (Tables 1 and S1†), with previously reported activity on PET or PET analogs, were expressed and partially purified *via* affinity chromatography utilizing their 6× His-tags. The set included the structurally characterized PETase from *Ideonella sakaiensis* (enzyme 2) that catalyzes PET hydrolysis at 30 °C but has limited activity on MHET.^9,10^ Several others were cutinases derived from the closely related thermophilic (∼55 °C growth optimum)^19^*Thermobifida* or *Thermomonospora* genera. Wild type and engineered variants of these enzymes were previously examined for their ability to hydrolyze PET, and optimal reaction temperatures have been observed as high as 65 °C.^20^ Additionally, nine other diverse esterases (10–18, Tables 1 and S2†) were obtained from Sigma. Enzymes were chosen to span a variety of known substrate specificities and host organisms. Two were previously characterized for activity on PET-related substrates, enzymes 11, a *B. subtilis* esterase (also described as *p*-nitrobenzylesterase)^21^ and 14, *Candida antarctica* lipase B (CalB). The latter was shown to exhibit both MHETase and BHETase activities.^22,23^ Both CalB and its paralog CalA (enzyme 13)^7,24–27^ are popular in biotechnological applications due to their broad substrate promiscuity. We anticipated the commercial enzymes would react optimally at ∼37 °C, except enzyme 10, which comes from a *Geobacillus stearothermophilus* strain with a growth optimum of 55 °C.^28^

### Protein expression, partial purification, and quantitation

Synthetic genes for each PET hydrolase (Table S1†) were codon-optimized for expression in *Escherichia coli* and cloned into the pET21b(+) expression vector (Twist Biosciences). Proteins were expressed using either the *E. coli* strain Lemo (DE3) (New England Biolabs) (enzyme 1), or the *E. coli* strain BL21 (DE3) (New England Biolabs) (enzymes 2–9). Transformants were inoculated 1 : 200 from an overnight culture into 500 mL of culture in 2.8 L Fernbach flasks containing 2× YT (16 g L^−1^ tryptone, 10 g L^−1^ yeast extract, 10 g L^−1^ NaCl) and 50 μg mL^−1^ ampicillin at 37 °C, with shaking at 220 rpm until optical density (OD_600_) reached 0.4–0.5. For each PET hydrolase, protein expression was induced with the addition of isopropyl-β-d-thiogalactopyranoside (IPTG) at a final concentration of 0.5 mM, followed by overnight (16 h) incubation at 25 °C. Cells were harvested by centrifugation at 8000 rpm (11 867 × *g*) for 20 min at 4 °C and stored at −80 °C. For purification, the cell pellets were resuspended in cell lysis buffer (20 mM Tris, 300 mM NaCl, 10 mM imidazole, pH 8.0) and sonicated on ice. The lysate was clarified by centrifugation at 12 000 rpm (17 370 × *g*) for 30 min at 4 °C. The supernatant was loaded onto a 10 mL His-Trap Ni-NTA column (MACLAB) equilibrated with washing buffer (20 mM Tris, 300 mM NaCl, 10 mM imidazole, pH 8.0), washed, then eluted in 50 mL elution buffer (20 mM Tris, 300 mM NaCl, 250 mM imidazole, pH 8.0). Fractions containing target protein (identified by SDS-PAGE) were pooled, concentrated, and buffer exchanged using Amicon Ultra Centrifugal Filters 15kDa MWCO into 20 mM Tris, 150 mM NaCl, pH 7.5. Expression and purification were not extensively optimized, approximating semi-pure conditions typical of a large-scale screen.

Protein concentrations in partially purified proteins were measured by Bradford assay. Target protein concentrations and purities were estimated by densitometry (ImageJ) (Fig. S2†). Additionally, SDS-PAGE was used to determine approximate molecular weights for each of the commercially supplied enzymes (Fig. S3†). Notably, stocks prepared by weight from commercially produced lyophilized powders varied widely in their protein content as measured by the Bradford assay, suggesting that differences in salt and buffer components added variable mass to the lyophilized powders. Concentrations of these stocks were consequently adjusted to 10 mg mL^−1^ protein as measured by the Bradford assay. Enzymes were stored in 5 mM BES or HEPPS buffer at −80 °C for up to one month prior to use in reactions. After thawing, enzyme activities were confirmed at a concentration of 0.1 μM in a solution of 2 mM *p*NP-butyrate at 37 °C.

### UV/visible properties of PR and BB indicators

UV/visible spectra for PR or BB were measured (Cary 60 spectrophotometer) at their reported p*K*_a_s (8.0 or 7.1, respectively), where the alkaline and acidic forms of the indicator are present in equivalent concentrations. Spectra were additionally measured 2 pH units above and below the p*K*_a_ in buffer (5 mM HEPPS or BES, Fig. 1), where the alkaline and acidic forms are expected to be present at 100-fold excess over the other form, respectively. Molar absorptivity constants (*ε*) were determined by measuring the absorbance (*A*) at or near the wavelength of maximal absorbance (*λ*_max_) as a function of concentration (*c*) and applying Beer's law 
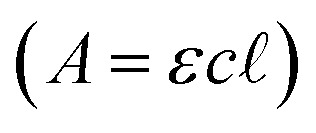
, for the alkaline form of the indicator.

### Temperature stability of PR

Freshly prepared 5 mM HEPPS buffer was equilibrated to 37 °C in a water bath and adjusted to pH 8.0. PR was added to the buffer to a final concentration of 0.1 mM and aliquoted 200 μL per well into a flat bottom 96-well plate. Plates were covered with a Bio-Rad ‘B’ seal to avoid evaporation, then placed in a 37 °C temperature-controlled plate reader. Absorbance at 550 nm (*A*_550_) was measured hourly for 4 days (Fig. S4†).

### Colorimetric activity assay

Directly before use, enzymes were diluted to a final concentration of 20 μM into either 5 mM HEPPS or 5 mM BES, pH 8.0 or 7.1, respectively. To each well of a flat bottom 96-well plate (Thermo Fisher Catalog No. 12565501), 100 μL of a 2× master mix was added. The master mix contained 2 mM BHET from a 50 mM stock in DMSO, 0.2 mM PR or BB indicator, 20 mM CaCl_2_ to assist with enzyme stability,^42,43^ and either 10 mM HEPPS (pH 8.0) or BES (pH 7.1) buffers (for a final reaction buffer concentration of 5 mM). DMSO was added to a total concentration of 20% v/v in the 2× master mix. Such concentrations of organic cosolvents are common in studies of ester hydrolases and other enzymes where the substrates and/or products are hydrophobic. Added DMSO may be omitted if more polar substrates/products are used. Reactions were initiated by adding 100 μL of 20 μM enzyme solution, resulting in a 10 μM reaction enzyme concentration, or 100 equivalents of BHET per enzyme (1 mM BHET or 2 mM total ester bonds). UV/visible absorbance at 550 nm (*λ*_max_ for PR_alkaline_) or 615 nm (*λ*_max_ for BB_alkaline_) was measured every 15 min for 24 h using a Varioskan Lux (Thermo Scientific) plate reader. Plates were covered in a translucent seal (Bio-Rad) to prevent evaporation and permit gentle shaking (60 rpm, orbital) between each reading.

The total amount of ester cleaved, based on the absolute value of the indicator absorbance change measured at a given time interval (|Δ*A*_550_| for PR), was computed as:1
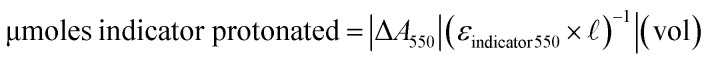

2 μmoles buffer protonated = [buffer]_total_[indicator]_total_^−1^ × eqn (1)

3
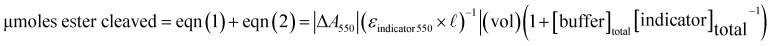
Here, *A*_550_ = the absorbance at 550 nm, 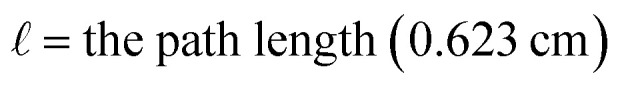, and vol = the reaction volume (200 μL). The subscript total refers to the combined concentrations of both the acidic and basic forms of either the buffer or indicator. The same expressions were applied to BB using its respective *ε* and absorbance at 615 nm. Specific activity was computed by referencing the total μmol ester cleaved per μmol enzyme during the initial reaction time period (45 min). Errors represent ±1 standard deviation. Two experimental replicate 96-well plates were measured, with each reaction present in triplicate per plate resulting in 6 total replicates per reaction.

### Upper and lower detection limits of the colorimetric assay

In the assay as described, complete hydrolysis of BHET to MHET would release 1 mM H^+^, with 98% of the H^+^ transferred to the buffer and 2% (0.02 mM) to the indicator. Further hydrolysis of MHET to TPA would result in equivalent amounts of H^+^ release. Assays were carried out at the p*K*_a_ of the indicator, at which 50% (0.05 mM) is in the alkaline, proton-accepting form. 5 mM buffer is sufficient to absorb the anticipated maximal influx of ∼1.96 mM H^+^ produced by ester cleavage, with the remaining 0.04 mM H^+^ reacting with the indicator. Hence, hydrolysis of an upper limit of 2.5 mM substrate would be detectable at these concentrations of indicator and buffer.

A lower limit of detectable ester hydrolysis was estimated as follows. A minimum measurable absorbance change of 0.01 units corresponds to a decrease in alkaline PR concentration of 0.3 μM [*c* = *Aε*^−1^*l*^−1^ = 0.01 × (46 000 M^−1^ cm^−1^)^−1^(0.623 cm)^−1^]. The uptake of H^+^ by the indicator is accompanied by a concomitant 50-fold greater (20 μM) influx of H^+^ to the buffer, based on their relative concentrations. Therefore, hydrolysis of ≥24 μM ester (1.2% of the 2 mM ester bonds in the diester substrate used here) should be readily measurable. In principle, assay sensitivity increases with larger enzyme concentrations, longer incubation periods, or the use of a microplate with a shorter path length. High sensitivity was chosen here, to eliminate any false negatives. By contrast, stringency is promoted by decreasing the concentration of enzyme and/or the assay time, resulting in the detection of higher activity levels.

### Colorimetric assays using freeze-dried cells

In addition to use with pure or semi-pure enzymes, the colorimetric assay was also investigated for suitability for use with unpurified, recombinantly expressed enzymes within lyophilized bacterial cells. *E. coli* Lemo 21 (DE3) cells expressing the *T. fusca* PET hydrolase (enzyme 1), as well as untransformed cells, were grown overnight, after which one mL of each culture was lyophilized. Reaction volumes of 10 mL contained 2 mM tributyrin, a known substrate for most cutinases and lipases, and 0.1 mM PR in HEPPS buffer, pH 8.0. Lyophilized cells were resuspended in HEPPS buffer, added to reaction volumes to initiate ester hydrolysis, and incubated at room temperature. UV/visible spectra were measured hourly using a Thermo Scientific Genesys 30 Visible Spectrophotometer. Results were compared to catalysis by the same strain not harboring a PET hydrolase plasmid (Fig. S5†).

### Product analyses

HPLC confirmed the identities and quantities of products and unreacted substrate. Reactions monitored by the colorimetric method were quenched at 24 h by adding 100 μL of the enzyme reaction to 100 μL pure, ice-cold methanol, followed by vortexing before storage at −80 °C. Prior to HPLC analysis, samples were centrifuged at 13 000 rpm (16 200 × *g*) for 5 min to pellet precipitated protein. 10 μL of the resulting supernatant was resolved on a Hypersil GOLD™ PFP HPLC C18 column (Thermo Scientific) using a Shimadzu Prominence-i LC-2050C HPLC instrument. Solvents used were HPLC-grade water with 0.1% trifluoroacetic acid (Solvent A) and HPLC-grade acetonitrile with 0.1% trifluoroacetic acid (Fisher Scientific, Solvent B). Reaction components were separated at a flow rate of 1 mL min^−1^ beginning with a column wash for 3 min with 5% B, from 3 to 6 min a 5% to 17% B gradient, from 6 to 9 min a 17% to 25% B gradient, from 9 to 15 min a 25% to 40% B gradient, and finally a wash of 100% B for 2 min. The column was re-equilibrated between samples with 5% B for 2 min. Standard solutions of 60, 125, 250, and 500 μM BHET, MHET (Sinfoo Biotech), and TPA were analyzed by the same protocol, and monitored *via* their absorbance at 243 nm (Fig. S6†). Auto integrated peak areas (LabSolutions software) were plotted as a function of standard concentrations from authentic material to generate standard curves. The functions derived from these standard curves were used to determine the concentrations of unknowns in reaction samples. BHET cleaved (mM) per μmol enzyme in the 200 μL reaction was calculated as:

4 [BHET] cleaved = ½[TPA] + [MHET]

### Applicability to solid phase PET

To each well of a flat bottom 96-well plate (Thermo Fisher Catalog No. 12565501) 100 μL of a 2× master mix was added. The master mix contained, 0.2 mM PR, 20 mM CaCl_2_ to assist with enzyme stability, and 5 mM HEPPS pH 8.0. DMSO was added to bring the final concentration to 20% v/v in the 2× master mix. To each well, one 6 mg amorphous PET coupon was added (Goodfellows 252-144-75). Reactions were initiated by adding 100 μL of 20 μM *I. sakaiensis* PETase (enzyme 2), resulting in a 10 μM enzyme concentration. UV/visible absorbance at 550 nm (*λ*_max_ for PR_alkaline_ was measured at 0, 3, 6, 10, 24, 48, 72, and 96 h using a Varioskan Lux (Thermo Scientific) plate reader). Samples were transferred to fresh wells before measurements to prevent scattering from the incident light of the spectrometer, then quenched with equivolume ice cold methanol for HPLC analysis (below) of products. Between reads, plates were covered in a translucent seal (Bio-Rad) to prevent evaporation and permit gentle shaking (60 rpm, orbital) between each reading and incubated at 37 °C.

HPLC confirmed the identities and quantities of products and unreacted substrate. Reactions monitored by the colorimetric method were quenched at 96 h by adding 100 μL of the reaction to 100 μL pure, ice-cold methanol, followed by vortexing before storage at −80 °C. Prior to HPLC analysis, samples were centrifuged at 13 000 rpm (16 200 × *g*) for 5 min to pellet precipitated protein. 10 μL of the resulting supernatant was resolved on a Hypersil GOLD™ PFP HPLC C18 column (Thermo Scientific) using a Shimadzu Prominence-i LC-2050C HPLC instrument as described above. Total products observed from colorimetric and HPLC measurements were computed as in eqn (1)–(4) and plotted in Fig. S7.†

## Results

### Optimizing assay conditions

**Fig. 2:**
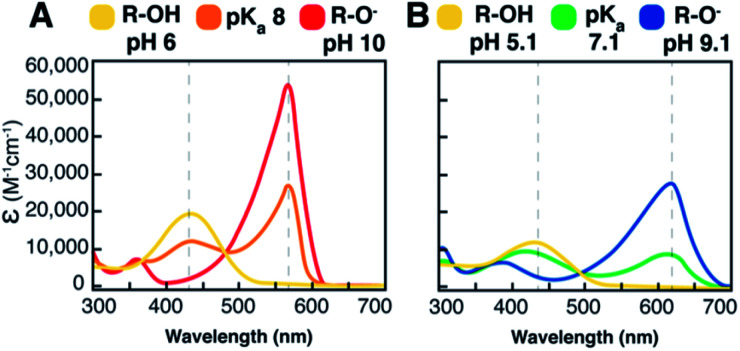
UV/visible absorption properties of the pH indicators used in this work. The extinction coefficients of (A) phenol red (PR) and (B) bromothymol blue (BB) are plotted as a function of wavelength (*λ*) at pH values equivalent to 2 units above and 2 units below their p*K*_a_s. The acidic form of PR has a *λ*_max_ at 434 nm and the alkaline form a *λ*_max_ at 550 nm, with a *λ*_isobestic_ at 480 nm. An extinction coefficient of 48 000 M^−1^ cm^−1^ was determined for the alkaline form of PR (red curve). The acidic form of BB has a *λ*_max_ at 434 nm and the alkaline form a *λ*_max_ at 615 nm, with a *λ*_isobestic_ at 500 nm. The alkaline form of BB (blue curve) had an extinction coefficient of 24 000 M^−1^ cm^−1^. A decrease in absorbance of the alkaline form of either indicator was monitored in the colorimetric assay. Determination of *ε* is shown in Fig. S8 and S9.†

Phthalein dye derivatives, particularly water soluble sulfonephthaleins, are widely used as quantitative indicators of pH, with p*K*_a_s spanning from 1.4 to 10.5 (Fig. S1†).^44,45^ The suitability of two commonly used sulfonephthaleins, PR and BB, were assessed here as assay constituents. First, we quantified the UV/visible properties of indicators having approximately neutral (BB) or higher (PR) p*K*_a_s (Fig. 2, S8 and S9†). UV/visible spectra illustrated both the intense absorptivity of the deprotonated/alkaline indicator in each case, as well as a lack of spectral overlap from the protonated/acidic form near the alkaline peak maxima (*λ*_max_). Monitoring conversion of each indicator's alkaline red (PR) or blue (BB) form to its acidic yellow form at a single wavelength is therefore uncomplicated.

We next examined the stability of the indicators at an elevated temperature (Fig. S4†). Incubating PR (or BB, data not shown) at 37 °C and 50 °C over 96 h resulted in a limited change in the absorbance spectrum, suggesting that the indicators remained stable over a period of days and at elevated temperature.

Concentrations of the indicators, buffers, substrate, and enzymes were optimized to maximize the assay sensitivity. For H^+^ detection, it is convenient to choose buffer/indicator pairs with the same H^+^ affinity (p*K*_a_). Under these conditions, the probability that either will bind the H^+^ reaction product is determined by their relative concentrations.^46^ When the assay pH = p*K*_a_ (buffer) = p*K*_a_ (indicator), half the concentration of indicator and buffer are deprotonated at the start of the assay. Under these conditions, an indicator concentration of 100 μM (50 μM in the alkaline form) yielded an absorbance near the saturation limit of the spectrometer and hence was adopted. A relatively high enzyme concentration (10 μM) was chosen to maximize the sensitivity of the assay and eliminate false negatives. 1 mM BHET was selected to be in 100-fold excess of the enzyme concentration, to maximize assay speed.

Under these conditions, specific activities could easily be determined from linear initial rates within 30–45 min of reaction initiation (Table S3†). Reaction endpoints were unequivocally reached by 24 h of incubation (Fig. S10†), at which point ≤20% of the initially present BHET was observed to be hydrolyzed in a no-enzyme control. Though not pursued here, secondary kinetic measurements on the subset of BHET-hydrolyzing enzymes identified in the initial, high-sensitivity screen could be carried out with less enzyme and varying substrate concentrations. We also observed that lyophilized cells in which production of the target enzymes was induced was likewise sufficient for qualitatively identifying enzyme activities (Fig. S5†). This enzyme format may be useful for a large-scale screen where purification of many proteins is impractical.

### Activity measurements against *p*NP-butyrate

To confirm that all enzymes were active, their hydrolytic activity was first measured with *para*-nitrophenol butyrate (*p*NP-butyrate), a widely used substrate (Scheme 1) that is rapidly hydrolyzed by most esterases. Reactions were conducted with 100-fold less enzyme than BHET reactions (0.1 μM) and 2 mM *p*NP-butyrate in 5 mM BES buffer, pH 7.1 at 37 °C for 45 min (Table S3†). While the whole enzyme set demonstrated activity, measured specific activities ranged widely, from 30–11 000 μmol ester hydrolyzed per minute per μmol enzyme, and an average value of 4000 μmol min^−1^ μmol^−1^ enzyme (standard deviation = 3200). This suggests a potentially broad range of intrinsic catalytic competencies for the enzyme set.

### Colorimetric screening identifies BHETases and likely MHETases

**Fig. 3:**
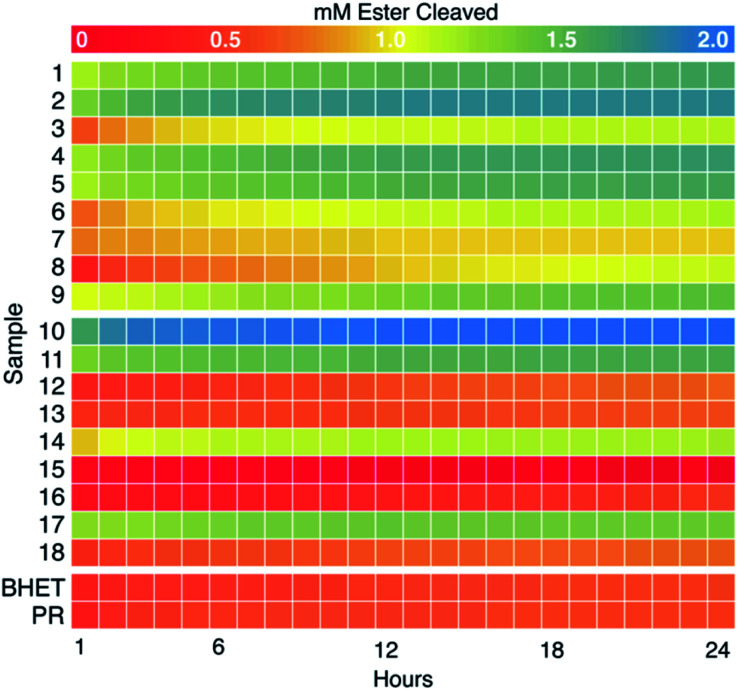
Colorimetric screening of PET hydrolases (1–9) and commercial esterases (10–18) was used to quantify hydrolysis of BHET and MHET over time. Heat map representing concentration of esters cleaved (using eqn (3)) at hourly time points over 24 h (pH 8.0, 37 °C, initial [BHET] = 1 mM) (see Fig. S7† for full absorbance *versus* time curves). H^+^ released as a result of ester hydrolysis was detected by PR and measured as a decrease in *A*_550_. Each data set was corrected for background acidification of the dye by enzyme in the absence of BHET. Data were averaged over two experimental replicates (plates) and three technical replicates (wells) per plate for a final *n* = 6. A no-enzyme control (labeled “BHET”) contained 5 mM HEPPS buffer, 0.1 mM PR, and 1 mM BHET. An indicator-only control (labeled “PR”) was the same, minus BHET. Heatmaps were generated in R using the pheatmap package.

We next examined the enzyme set for BHET hydrolysis over time at pH 8 and 37 °C. Reaction progress curves (concentration of ester cleaved as a function of time) are reported in Fig. S10.† These show a significant increase in product concentration during the initial hour of incubation, followed by slower conversion of substrate to product for the remainder of the 24 hour incubation. Points measured hourly are presented as a heatmap in Fig. 3. Colors proceeding from red to yellow on this plot indicate up to 1 mM of ester cleavage (*i.e.*, BHET conversion to MHET and possibly overlapping MHET conversion to TPA). Further conversion from yellow to blue corresponds to up to an additional ∼1.0 mM of ester cleaved, indicating that an enzyme displays activity against both BHET and MHET, yielding TPA. Data have been corrected by subtraction of an indicator/enzyme control measured for each enzyme, to control for acidification of the indicator by the enzyme. A small but noticeable acidification effect was only observed when PR was mixed with a few of the lyophilized commercial enzymes, in which buffer salts and/or other additives were far more abundant by mass than protein. Data were not otherwise corrected. Indicator-only and substrate/indicator controls labeled PR and BHET, respectively are shown in Fig. 3.

For PET-active enzymes 1–9, hydrolysis well above the no-enzyme controls was observed in every case. Enzymes 3 and 6–8 exhibited ≤1 mM of ester cleavage, while 1, 2, 4, 5, and 9 exceeded 1 mM cleavage and therefore appeared to hydrolyze both BHET and MHET. Among the commercial enzymes, the majority did not catalyze substantial BHET hydrolysis, even under the high enzyme loading conditions used here. However, enzyme 18 exhibited cleavage of ∼0.5 mM ester, while 10, 11, and 17 hydrolyzed >1 mM. Therefore, we conclude that the latter three enzymes catalyze hydrolytic activity with both BHET and MHET, with enzyme 10 exhibiting the greatest substrate turnover of the entire collection screened, including the known PET hydrolases.

Data measured over the first 45 min of the reactions yielded linear product profiles as a function of time, which were fitted to linear equations to determine specific activities (Table S3†). Measured specific activities against BHET were generally ≥1000-fold slower than those measured with *p*NP-butyrate, justifying the relatively high concentration of enzymes used in the BHET screen. For the most part, enzymes demonstrating activity with BHET by 24 h had comparable specific activities, ranging from 1–3 μmol ester cleaved per min per μmol enzyme, with one exception. Enzyme 9 was several-fold slower (0.2 μmol ester cleaved per min per μmol enzyme) than others in the set but retained enough activity over the course of the 24 h incubation to hydrolyze approximately 2 mM ester. While a short screen (<1 h) of specific activities would have been sufficient to identify most of the enzymatic “hits”, this “slow and steady” candidate might have otherwise been discarded.

### HPLC product analyses distinguish BHETase and MHETase activity

**Fig. 4:**
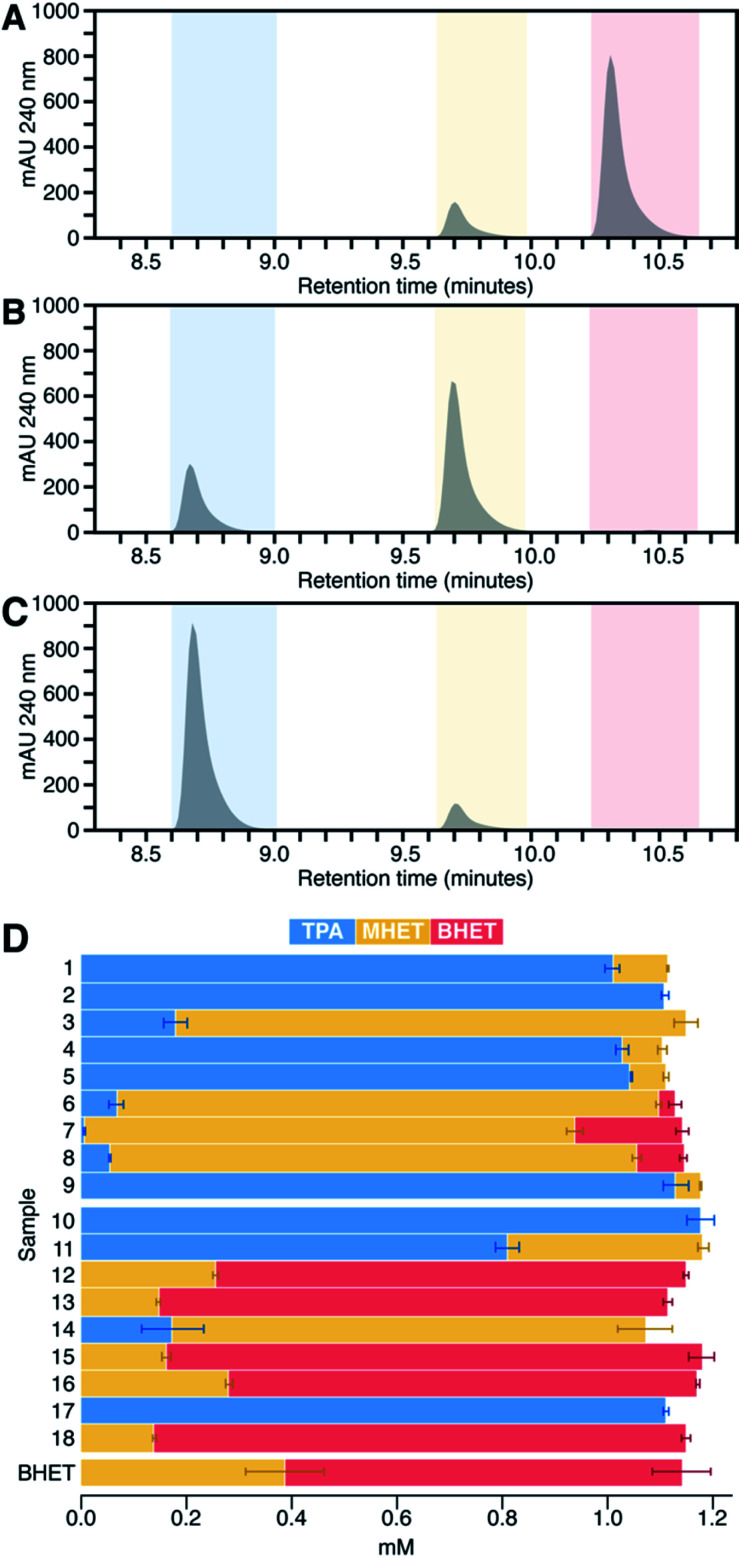
HPLC analysis was used to quantify unreacted BHET and its hydrolysis products MHET and TPA in reactions. Chromatagrams (A–C) and quantification (D) of unreacted BHET and reaction products measured by HPLC at 240 nm from assays run for 24 h at pH 8.0 and 37 °C. (A–C) Retention time zones are color-coded for BHET (red), MHET (yellow), and TPA (blue). Chromatograms are shown for single reaction samples carried out by three different enzymes, representative of the possible ester hydrolysis profiles observed. (A) Enzyme 13, a poor BHETase, closely matches the HPLC profile of the no enzyme control (see ESI†), with no discernible change in peak heights over 24 h. (B) The enzyme 14 profile indicates a good BHETase but poor MHETase, reflected by an accumulation of MHET. (C) Enzyme 1 is both a good BHETase and MHETase, with TPA as the major product and no detectable remaining BHET. (D) Bar chart showing concentrations of BHET (red), MHET (yellow), and TPA (blue) averaged from HPLC quantifications of reactions from two 96 well plates with triplicate reactions per plate (*n* = 6 for each enzyme). Uncatalyzed BHET hydrolysis is responsible for converting a fraction of the initially available BHET to MHET during the 24 h of incubation of the no-enzyme control (labeled “BHET”).

To quantify BHET *versus* MHET hydrolysis explicitly, we analyzed the reactions for unreacted BHET and products at the end of the 24 h incubation by HPLC (Fig. 4). Of the PET hydrolases, enzymes 3, 6, 7, and 8 acted primarily as BHETases, hydrolyzing one ester bond of BHET to yield the MHET monoester as the major product. In some cases, a small amount of the MHET hydrolysis product (TPA) was also observed. Enzymes 1–2, 4–5, and 9 exhibited both BHETase and MHETase activities, each having TPA as their major reaction product. These results mirror expectations based on the colorimetric screen. Similarly, consistent with the screen presented in Fig. 3, we did not observe product formation in excess of the no-enzyme controls for most of the commercial enzymes. Enzyme 14 reacted as a BHETase, converting all of the available BHET to MHET, with TPA as a minor product. Enzymes 10, 11, and 17 showed both BHETase and MHETase activity, converting most of the available BHET to TPA.

In no case were BHET and TPA observed together in significant concentrations. This suggested that the conditions used here (high enzyme loading and long incubation periods) were biased toward identifying enzymes with even small amounts of MHET-directed activity from among the BHETases. As an important example, enzyme 2 from *I. sakaiensis* previously exhibited little measurable MHET hydrolytic activity under conventional steady state conditions, and yielded only small amounts of TPA when incubated for several hours with solid PET.^9,10,17^ However, the same enzyme was shown to hydrolyze much of the available MHET under the high enzyme loading and 24 h incubation conditions used here.

### Variable pH and temperature conditions were likewise amenable to the assay

**Fig. 5:**
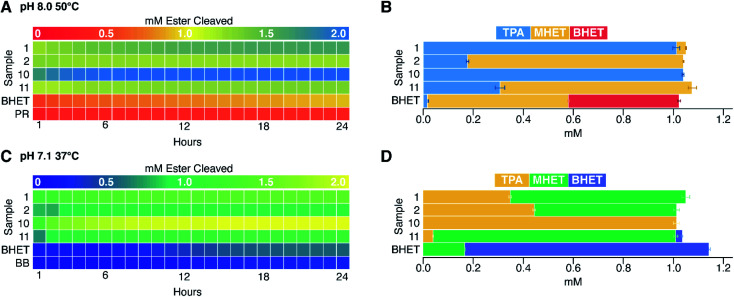
BHET hydrolysis was measured at alternate values of pH and temperature for selected enzymes. Heat maps representing total concentration of esters cleaved at hourly time points over 24 h (initial [BHET] = 1 mM) at pH 8.0, 50 °C, using PR indicator (A) and at pH 7.1, 37 °C, using BB indicator (C). HPLC analyses of unreacted substrate and products at 24 h, averaged (*n* = 6) from the reactions shown in panels (A) and (C) are shown to their right in panels (B) and (D), respectively. Non-enzymatic cleavage of BHET (labeled “BHET”) was higher in assays conducted at 50 °C than those at 37 °C, and lower at pH 7.1 than pH 8. Indicator-only controls without enzyme or BHET are labeled as PR (A) and BB (C).

Finally, to address the potential influences of pH and temperature on assay function, we re-screened a subset of four enzymes at the same temperature (37 °C) but a lower pH (7.1), or at the same pH (8.0) but a higher temperature, 50 °C (Fig. 5). Enzymes 1 and 2 are respectively thermostable and mesostable. Enzyme 10 is an esterase from *G. stearothermophilus*, a strain with a temperature optimum of 55 °C and limited tolerance to acid.^28,47^ Enzyme 11 is from *B. subtilis*, a species that grows best at 37 °C and pH 5.5.^48^

Consistent with expectations based on strain backgrounds, the enzymes from thermostable organisms (enzymes 1 and 10) both retained activity at 50 °C, while the enzymes from mesophiles (enzymes 2 and 11) hydrolyzed less substrate than they had at 37 °C. By contrast, a decrease of pH from 8.0 to 7.1 diminished turnover in each case except for enzyme 10, despite the host organism's acid sensitivity. Lastly, BHET appeared to be substantially more stable at lower pH and temperature, showing less baseline non-enzymatic hydrolysis.

### Hydrolysis of solid amorphous PET monitored by colorimetric and HPLC methods yields similar results

To assess the applicability of the colorimetric method to a solid phase substrate, we monitored hydrolysis of solid, amorphous PET film by colorimetric and HPLC based methods in parallel. These methods afforded similar quantitative results. However, because plates were monitored using a spectrophotometric light source underneath the wells, samples had to be transferred to fresh wells prior to reading, lowering the attainable throughput.

## Discussion

Over 200 enzyme families share an α/β hydrolase fold, which serves as the structural support for a variety of biocatalytic hydrolytic reactions.^57^ The majority possess a canonical catalytic triad of amino acids at their active site – a serine nucleophile activated by hydrogen-bonding to a histidine–aspartate pair – which initiates cleavage of the ester bond. What distinguishes these otherwise similar enzymes, both in their natural contexts and in biotechnological applications, is their ability to interface with specific esters as substrates. The widely used *p*NP-ester reporter substrates (Scheme 1), while inexpensive and easily adaptable to microplate formats, are insufficient for sorting esterases with desired substrate preferences, as their variability generally lies in hydrocarbon tail length, which poorly represents the diversity of possible ester-containing substrates. The same methods are also incapable of elucidating active site structure–activity relationships in important esterases of any subtype. By contrast, ultrahigh pressure liquid chromatography coupled to mass spectrometry (UHPLC-MS) is capable of detecting reaction products from diverse substrates; however, high quality UHPLC-MS instrumentation is expensive, and the robotic autosampling required for high throughput screening of thousands of samples is not available in many labs.

We optimized time-resolved, proton-sensing colorimetric assays to screen both known PET hydrolases and diverse commercial enzymes for hydrolytic activity against the PET-derived substrate, BHET. Esterases with BHETase specificity are desirable as members of PET-degrading cocktails in conjunction with PET- and MHET-hydrolyzing enzymes, and for upcycling of pure BHET produced by chemical glycolysis. Additionally, H^+^ released from ester hydrolysis was quantified over timescales appropriate for both initial rate (specific activity) and reaction-endpoint (total turnover) assessments *via* a colorimetric, UV/visible assay. This method utilized two members of a family of sulfonephthalein indicators (PR and BB) and aminosulfonate buffers with matched p*K*_a_s. Though our focus was on comparing reactions at pH 7.1 and 8.0 (Fig. 5), the use of additional members of these indicator/buffer families extends the potential application of these screening methods to a wide range of biologically accessible pH values (Fig. S1†).^45^ The same assay could likewise be used with virtually any ester substrate with a suitably acidic carboxylic acid product, including solid phase amorphous PET (Fig. S9†).

The tested enzymes included previously described PET hydrolases, which we assumed would display variable levels of BHET hydrolysis. This enzyme group also serves as a representative set of catalysts to be further refined by evolution or engineering approaches for biotechnological application.^52,53,55,56^ Though not pursued here, the flexibility and throughput of this assay would be ideal for use comparing the activity of wild-type enzymes to such a panel of mutants against native or non-native substrates. Additionally, we examined commercial esterases, which may better simulate the type of highly diverse enzyme sets obtained in a library screen or an environmental sample.^51,54^

All nine PET hydrolases were active as BHET hydrolases. Of these, five (originating from *I. sakaiensis* and a variety of *T. fusca* strains) additionally hydrolyzed MHET, even though BHET is neutral and MHET is negatively charged. Interestingly, the *I. sakaiensis* enzyme (enzyme 2) was not previously identified as an effective MHETase under steady-state conditions (high substrate and low enzyme concentrations).^9,10,17^ However, the relatively weak endogenous MHETase activity of this enzyme was captured by the high sensitivity, low stringency conditions chosen for the screen used here. These conditions could be modulated to increase stringency by decreasing enzyme loading and/or reducing the assay time, thereby excluding enzymes with low activity levels.

Four commercial enzymes, enzymes 10, 11, 14, and 17, hydrolyzed BHET. Two of these, CalB from *C. antarctica* (enzyme 14) and an esterase from *B. subtilis* (enzyme 11), had previously been identified as having activity on PET-related substrates.^22,41^ While CalB was specific for BHET, the remaining three enzymes hydrolyzed both BHET and MHET. Enzyme 10, from *Geobacillus stearothermophilus* (previously known as *Bacillus stearothermophilus*), was not previously known to hydrolyze any PET-related substrate. Consistent with the parent strain's 55 °C growth optimum, enzyme 10 retained its activity at both the 37 and 50 °C conditions examined here.

The *B. subtilis* esterase (enzyme 11) is a widely-used commercial enzyme^21,59,60^ with, by contrast, a conventional, apparently buried active site. In a 2012 paper by Ribitsch *et al.*, this group measured the enzyme's activity against a synthesized PET trimer (3PET), noting that turnover from PET to BHET and MHET was fast, while the secondary conversion of MHET to TPA was slower.^41^ Sequence alignment of the *B. subtilis* esterase and the *I. sakaiensis* PETase through Emboss Needle^61^ demonstrated very little conservation and only 17.1% similarity (Fig. S11†). *G. stearothermophilus* esterase has only a slightly higher percent similarity at 18.1% (Fig. S12†). As expected, the catalytic Ser and His residues were conserved in all sequences, but the catalytic Asp was replaced by a glutamic acid at position 310 in enzyme 11.

**Fig. 6:**
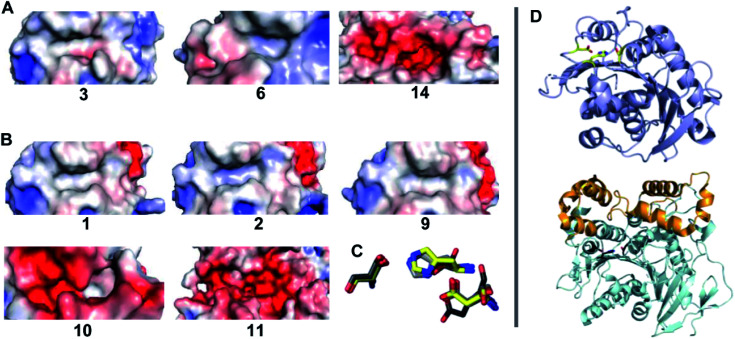
Electrostatic mapping of the active sites of enzymes with available crystallographic structures is not predictive of catalytic activity with BHET *versus* MHET. Substrate binding regions near the active site serine–histidine–aspartate/glutamate triad of residues are shown for (A) BHET-active enzymes 3 (PDB ID 4EB0), 6 (PDB ID 5LUJ), and 14 (PDB ID 6J1T) and (B) BHET/MHET active enzymes 1 (PDB ID 4CG1), 2 (PDB ID 6EQD), 9 (PDB ID 5ZOA) 10 (PDB ID 1TQH) and 11. Electrostatic potential distribution was calculated using APBS^58^ and mapped to the solvent-accessible surface of the enzymes as a colored gradient from red (acidic) at −5 *kT*/*e* to blue (basic) at 5 *kT*/*e* (where *k* is Boltzmann's constant, *T* is temperature and *e* is the charge on an electron and presented in a similar orientation). (C) The side chains of the catalytic triad of residues for all 8 structures are shown as an overlay. Side chains of enzymes 1 and 11 are shown in yellow and black respectively, and all others are in varying shades of grey. The catalytic glutamate residue of enzyme 11 has the highest degree of displacement in this active site alignment. (D) The compact structure of enzyme 1 is rendered as cartoon with the triad side chains highlighted in yellow (top). The larger enzyme 11 is shown with the catalytic domain in a similar orientation in light cyan, the lid domain highlighted orange, and the triad side chains in black.

The identification of five BHET-specific enzymes in this study is significant for applications where activity with specific PET-derived substrates is important for formulating enzyme cocktails. It also suggests the potential interest in using diverse substrates to probe substrate–activity relationships in enzymes from this family. We examined the available enzymatic crystal structures (Fig. 6) to see if their selectivity for BHET and/or MHET substrates could be rationalized in a straightforward way. Two features are notable. First, the structurally characterized PET hydrolase from *I. sakaiensis* (enzyme 2) possesses a largely surface-exposed active site inside a narrow groove bordered by aromatic residues, an arrangement conserved in the other PETases in Fig. 6. Exposure may allow the enzyme to interact with the surface of solid PET, where the aromatic side chains appear to act as guiderails for individual polymer chains of the PET substrate.^50,62,63^ Exposure may also leave the active site relatively open to binding a variety of small molecules. By contrast, the BHET/MHET-active enzymes 10 and 11, from *G. stearothermophilus* and *B. subtilis* respectively, have lid domains extending above their respective active sites.^28^ The lid domain encloses the active site, forcing the substrate to enter through a structurally defined opening that could serve as a specificity filter. However, here we observed both lidded and unlidded active sites having BHETase or combined BHETase/MHETase activity, indicating that active site accessibility does not decide specificity. Second, electrostatic maps of the protein surfaces surrounding the catalytic triad did not reveal a consistent pattern of hydrophobicity (exemplified by enzymes 3 and 6, and 1, 2, and 9) or hydrophilicity (enzymes 14, 10, and 11) between the BHETase and BHETase/MHETase sets, though BHET is highly hydrophobic and MHET is expected to be negatively charged. These results suggest that simple structural analyses were not predictive of substrate preference in this case, underscoring the relevance of functional screening.

Our results demonstrate the ease, adaptability, and cost effectiveness of the screening methods used here, which are amenable to virtually any ester substrate with a suitably acidic carboxylic acid product, over a range of timescales, temperatures, pHs, and enzymes. Important limitations of the approach include its sensitivity to acidic reaction constituents, as we noted with lyophilized commercial enzymes that contained large amounts of buffering salts. UHPLC in conjunction with robotic autosampling systems, while costly, may offer the most flexible, high-throughput alternative. Expanded use of these high-throughput methods holds great potential for further such insights, with possible applications for directed evolution and mutant library screening, bioprospecting, and characterization of engineered esterases.

## Author contributions

J. L. B.: investigation, methodology, conceptualization, visualization, writing – original draft; R. C.: investigation, visualization, writing – original draft; W. M. K.: investigation, methodology, writing – review & editing; E. E.: resources, methodology, conceptualization, writing – review & editing; J. E. M.: project administration, conceptualization, methodology; G. T. B.: project administration, funding acquisition, conceptualization, methodology, writing – review & editing; J. L. D.: project administration, conceptualization, methodology, funding acquisition, supervision, writing – original draft.

## Conflicts of interest

There are no conflicts to declare.

## Supplementary Material

RA-012-D2RA00612J-s001

RA-012-D2RA00612J-s002

RA-012-D2RA00612J-s003

RA-012-D2RA00612J-s004

RA-012-D2RA00612J-s005

RA-012-D2RA00612J-s006

RA-012-D2RA00612J-s007

RA-012-D2RA00612J-s008

RA-012-D2RA00612J-s009

RA-012-D2RA00612J-s010

RA-012-D2RA00612J-s011

RA-012-D2RA00612J-s012

RA-012-D2RA00612J-s013

RA-012-D2RA00612J-s014

RA-012-D2RA00612J-s015

RA-012-D2RA00612J-s016

RA-012-D2RA00612J-s017

RA-012-D2RA00612J-s018

RA-012-D2RA00612J-s019

RA-012-D2RA00612J-s020

RA-012-D2RA00612J-s021

RA-012-D2RA00612J-s022

RA-012-D2RA00612J-s023

RA-012-D2RA00612J-s024

RA-012-D2RA00612J-s025

RA-012-D2RA00612J-s026

RA-012-D2RA00612J-s027

RA-012-D2RA00612J-s028

RA-012-D2RA00612J-s029

RA-012-D2RA00612J-s030

RA-012-D2RA00612J-s031

RA-012-D2RA00612J-s032

RA-012-D2RA00612J-s033

RA-012-D2RA00612J-s034

RA-012-D2RA00612J-s035

RA-012-D2RA00612J-s036

RA-012-D2RA00612J-s037

RA-012-D2RA00612J-s038
