# Supplementary material for: A flexible kinetic assay efficiently sorts prospective biocatalysts for PET plastic subunit hydrolysis
Source: RSC Adv. 2022 Mar 14;12(13):8119–30. doi: 10.1039/d2ra00612j (PMC8982334; doi:10.1039/d2ra00612j)
Supplement: RA-012-D2RA00612J-s005 [file RA-012-D2RA00612J-s005.pdf]

SHIMADZU LabSolutions

Analysis Report

<Sample Information>

Sample Name	: 0 hr Control ER2 pH7		
Sample ID	:		
Data Filename	: 0 hr Control ER2 pH7_049.lcd		
Method Filename	: MHET_BHET_rpamide_060721.lcm		
Batch Filename	: BHET_Colorimetric_37C_pH7_09072021.lcb		
Vial #	: 3-33	Sample Type	: Unknown
Injection Volume	: 10 uL		
Date Acquired	: 9/8/2021 5:02:54 AM	Acquired by	: System Administrator
Date Processed	: 9/8/2021 10:30:52 AM	Processed by	: System Administrator

<Chromatogram>

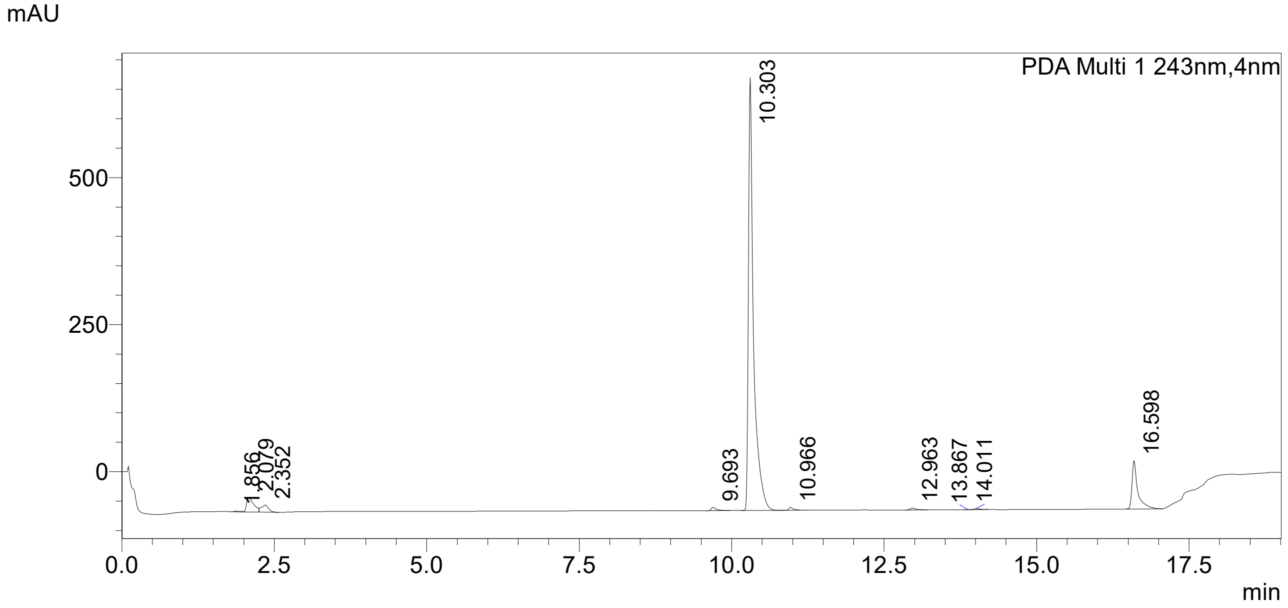

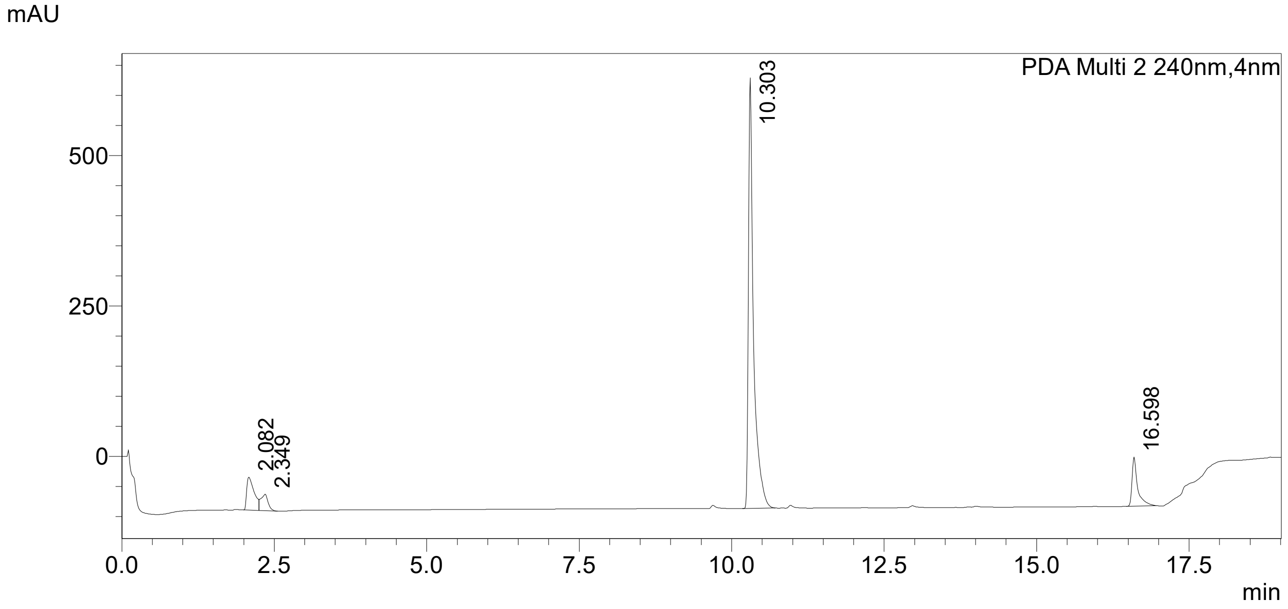

<Peak Table>

PDA Ch1 243nm

Peak#	Ret. Time	Area	Height	Conc.	Unit	Mark	Name
1	1.856	9090	1023	0.000			
2	2.079	204331	24304	0.000		V	
3	2.352	98509	11947	0.000		V	
4	9.693	32478	5503	-2.567	uM		MHET
5	10.303	4373374	736075	446.641	uM		BHET
6	10.966	30307	4931	0.000		V	
7	12.963	21878	3269	0.000			
8	13.867	3645	690	0.000			
9	14.011	11395	1739	0.000		V	
10	16.598	542347	82006	0.000			
Total		5327355	871487				

PDA Ch2 240nm

Peak#	Ret. Time	Area	Height	Conc.	Unit	Mark	Name
1	2.082	477948	54609	0.000			
2	2.349	221586	27334	0.000		V	
3	10.303	4236645	715402	0.000			
4	16.598	523463	81123	0.000			
Total		5459643	878468				
