# Supplementary material for: A flexible kinetic assay efficiently sorts prospective biocatalysts for PET plastic subunit hydrolysis
Source: RSC Adv. 2022 Mar 14;12(13):8119–30. doi: 10.1039/d2ra00612j (PMC8982334; doi:10.1039/d2ra00612j)
Supplement: RA-012-D2RA00612J-s006 [file RA-012-D2RA00612J-s006.pdf]

SHIMADZU LabSolutions

Analysis Report

<Sample Information>

Sample Name	: 24 hr Control ER1 pH7		
Sample ID	:		
Data Filename	: 24 hr Control ER1 pH7_030.lcd		
Method Filename	: MHET_BHET_rpamide_060721.lcm		
Batch Filename	: BHET_Colorimetric_37C_pH7_09072021.lcb		
Vial #	: 3-14	Sample Type	: Unknown
Injection Volume	: 10 uL		
Date Acquired	: 9/7/2021 10:55:25 PM	Acquired by	: System Administrator
Date Processed	: 9/8/2021 10:30:17 AM	Processed by	: System Administrator

<Chromatogram>

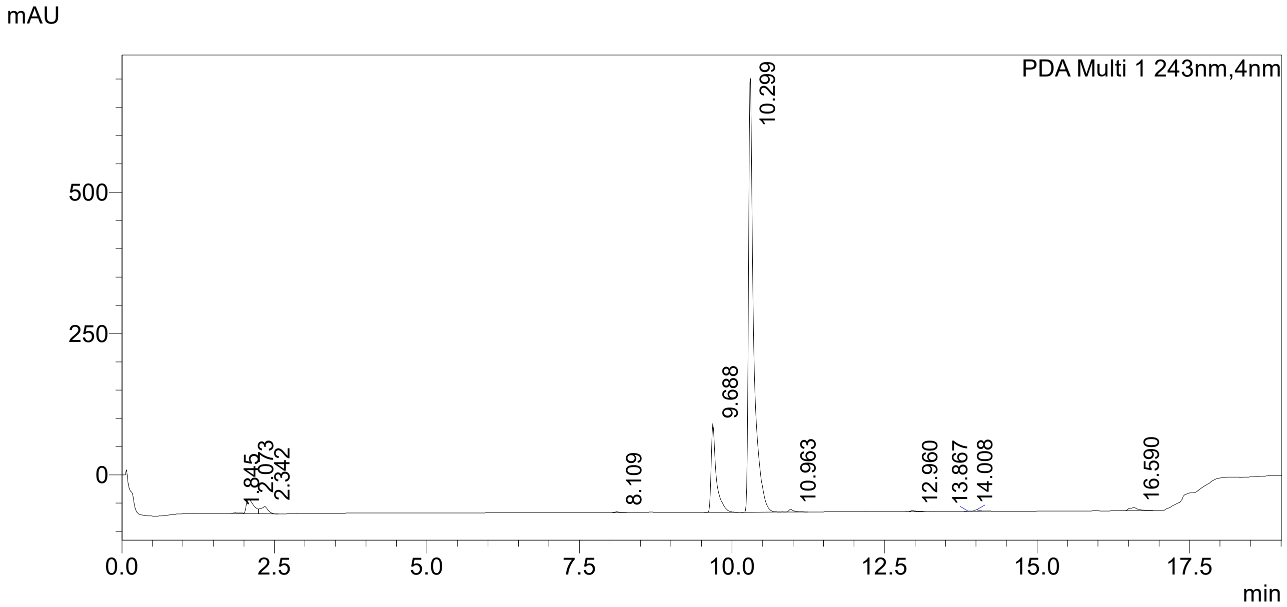

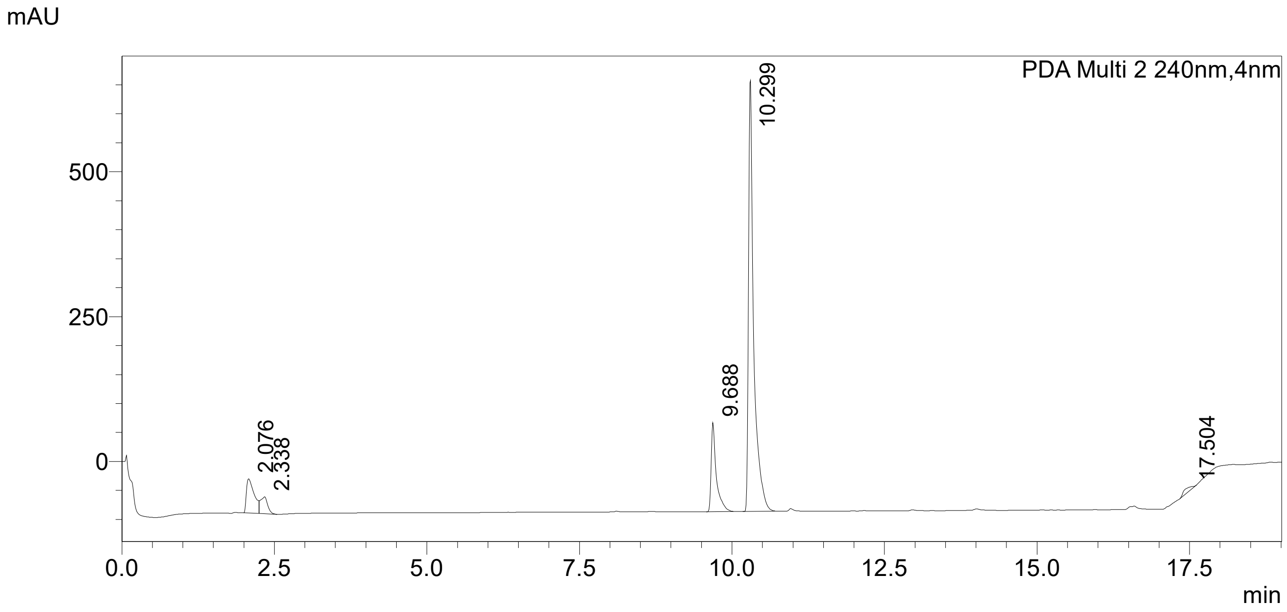

<Peak Table>

PDA Ch1 243nm

Peak#	Ret. Time	Area	Height	Conc.	Unit	Mark	Name
1	1.845	10013	1122	0.000			
2	2.073	226442	26171	0.000		V	
3	2.342	108462	12787	0.000		V	
4	8.109	5898	1244	0.000			
5	9.688	921253	155036	81.925	uM		MHET
6	10.299	4634348	765428	474.487	uM		BHET
7	10.963	29664	4782	0.000		V	
8	12.960	10779	1683	0.000			
9	13.867	3738	743	0.000			
10	14.008	18639	2639	0.000		V	
11	16.590	59822	5890	0.000			
Total		6029057	977524				

PDA Ch2 240nm

Peak#	Ret. Time	Area	Height	Conc.	Unit	Mark	Name
1	2.076	542924	58986	0.000			
2	2.338	231622	29345	0.000		V	
3	9.688	904306	153211	0.000			
4	10.299	4489777	743877	0.000			
5	17.504	86414	6211	0.000			
Total		6255042	991629				
