# Supplementary material for: A flexible kinetic assay efficiently sorts prospective biocatalysts for PET plastic subunit hydrolysis
Source: RSC Adv. 2022 Mar 14;12(13):8119–30. doi: 10.1039/d2ra00612j (PMC8982334; doi:10.1039/d2ra00612j)
Supplement: RA-012-D2RA00612J-s007 [file RA-012-D2RA00612J-s007.pdf]

SHIMADZU LabSolutions

Analysis Report

<Sample Information>

Sample Name	: E1 pH7		
Sample ID	:		
Data Filename	: E1 pH7 _019.lcd		
Method Filename	: MHET_BHET_rpamide_060721.lcm		
Batch Filename	: BHET_Colorimetric_37C_pH7_09072021.lcb		
Vial #	: 3-3	Sample Type	: Unknown
Injection Volume	: 10 uL		
Date Acquired	: 9/7/2021 7:22:41 PM	Acquired by	: System Administrator
Date Processed	: 9/8/2021 10:29:57 AM	Processed by	: System Administrator

<Chromatogram>

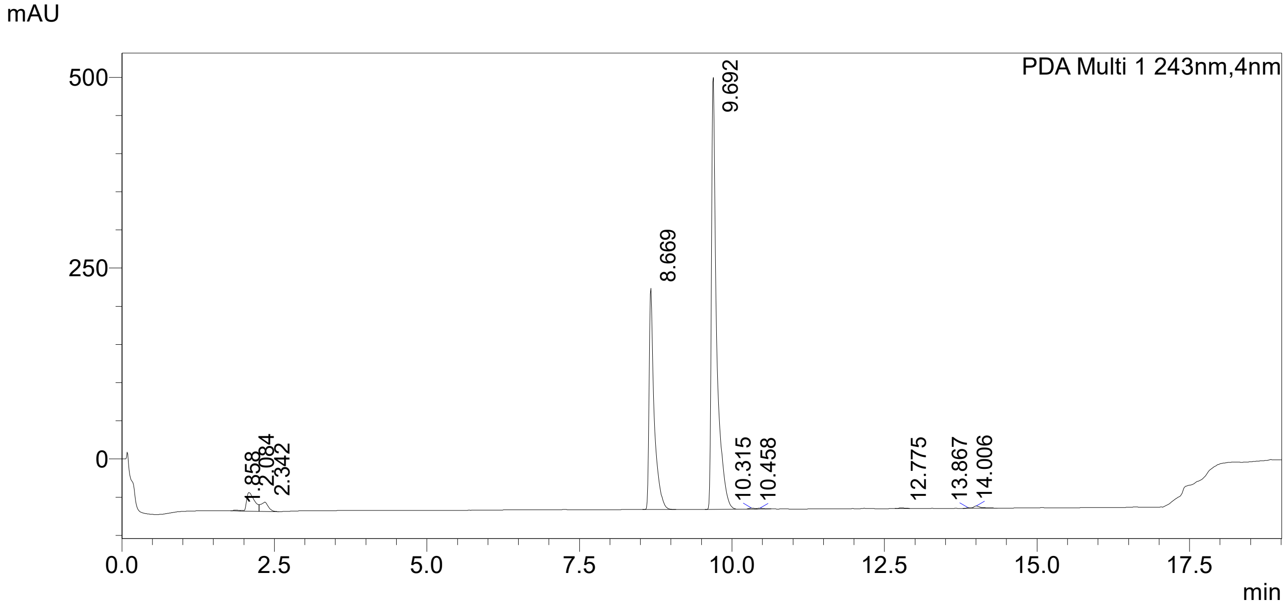

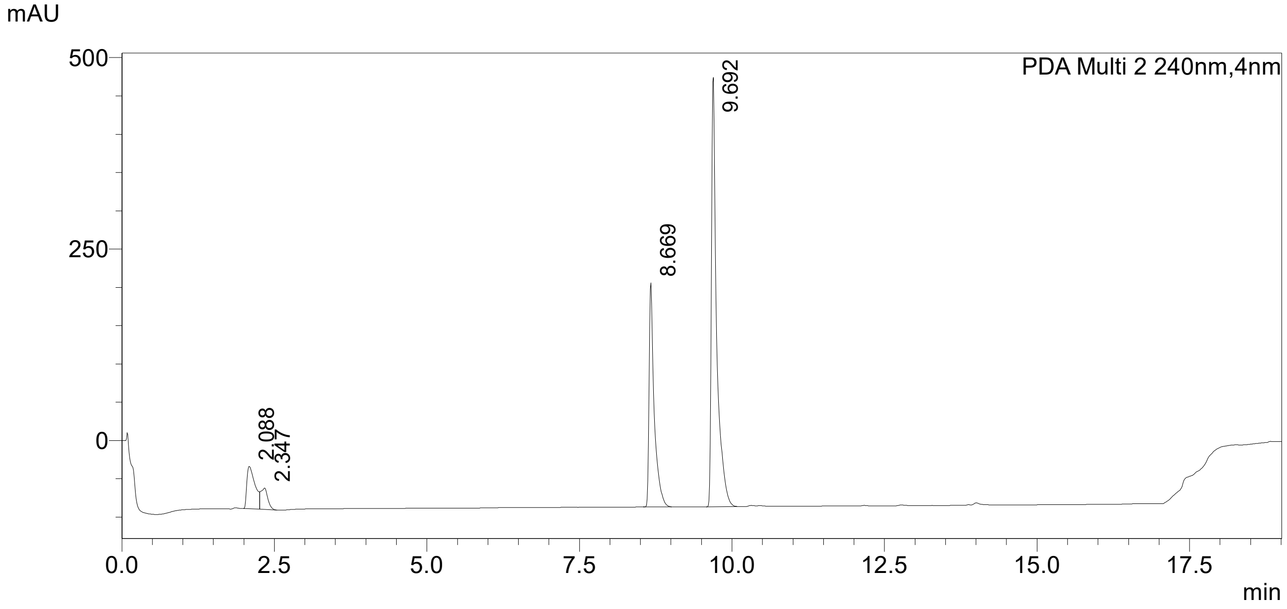

<Peak Table>

PDA Ch1 243nm

Peak#	Ret. Time	Area	Height	Conc.	Unit	Mark	Name
1	1.858	7486	1227	0.000			
2	2.084	225737	24326	0.000		V	
3	2.342	101521	12106	0.000		V	
4	8.669	1712815	290059	0.000			
5	9.692	3413818	565814	324.502	uM		MHET
6	10.315	10768	1526	-4.895	uM		BHET
7	10.458	8155	1255	0.000		V	
8	12.775	5684	1017	0.000			
9	13.867	4080	786	0.000			
10	14.006	25396	3060	0.000		V	
Total		5515460	901177				

PDA Ch2 240nm

Peak#	Ret. Time	Area	Height	Conc.	Unit	Mark	Name
1	2.088	535721	55277	0.000			
2	2.347	214063	27594	0.000		V	
3	8.669	1723938	292729	161.765	uM		TPA
4	9.692	3375466	560667	0.000			
Total		5849188	936267				
