# Supplementary material for: A flexible kinetic assay efficiently sorts prospective biocatalysts for PET plastic subunit hydrolysis
Source: RSC Adv. 2022 Mar 14;12(13):8119–30. doi: 10.1039/d2ra00612j (PMC8982334; doi:10.1039/d2ra00612j)
Supplement: RA-012-D2RA00612J-s008 [file RA-012-D2RA00612J-s008.pdf]

SHIMADZU LabSolutions

Analysis Report

<Sample Information>

Sample Name	: E10 pH 7		
Sample ID	:		
Data Filename	: E10 pH 7_043.lcd		
Method Filename	: MHET_BHET_rpamide_060721.lcm		
Batch Filename	: BHET_Colorimetric_37C_pH7_09072021.lcb		
Vial #	: 3-27	Sample Type	: Unknown
Injection Volume	: 10 uL		
Date Acquired	: 9/8/2021 3:06:52 AM	Acquired by	: System Administrator
Date Processed	: 9/8/2021 10:30:41 AM	Processed by	: System Administrator

<Chromatogram>

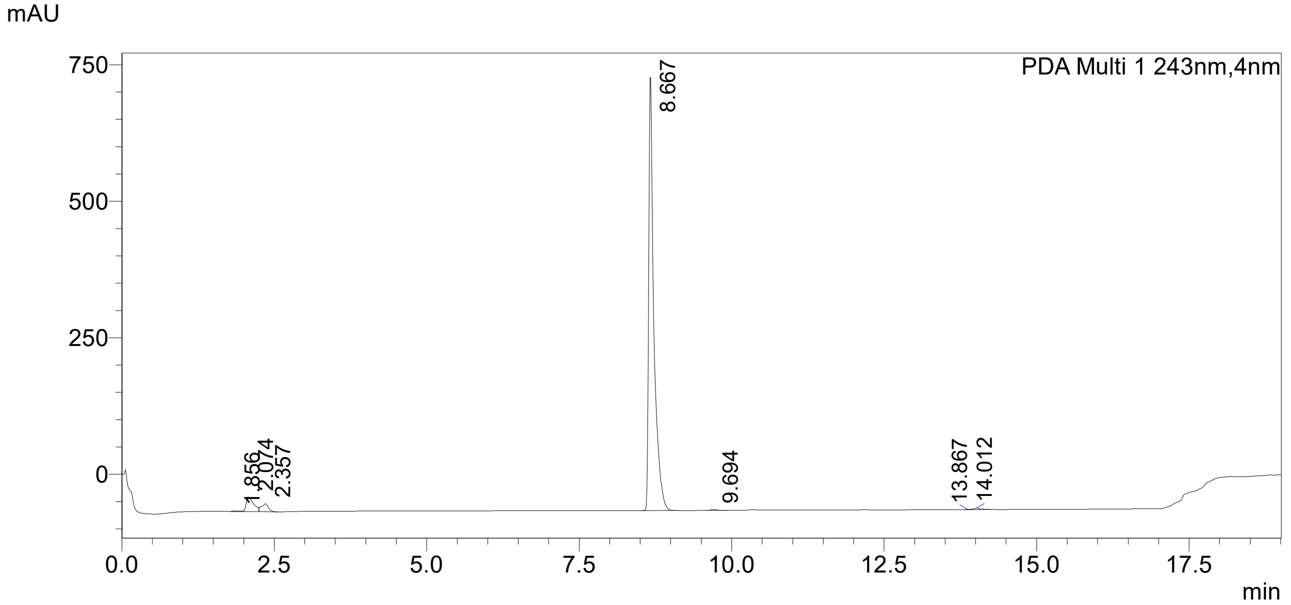

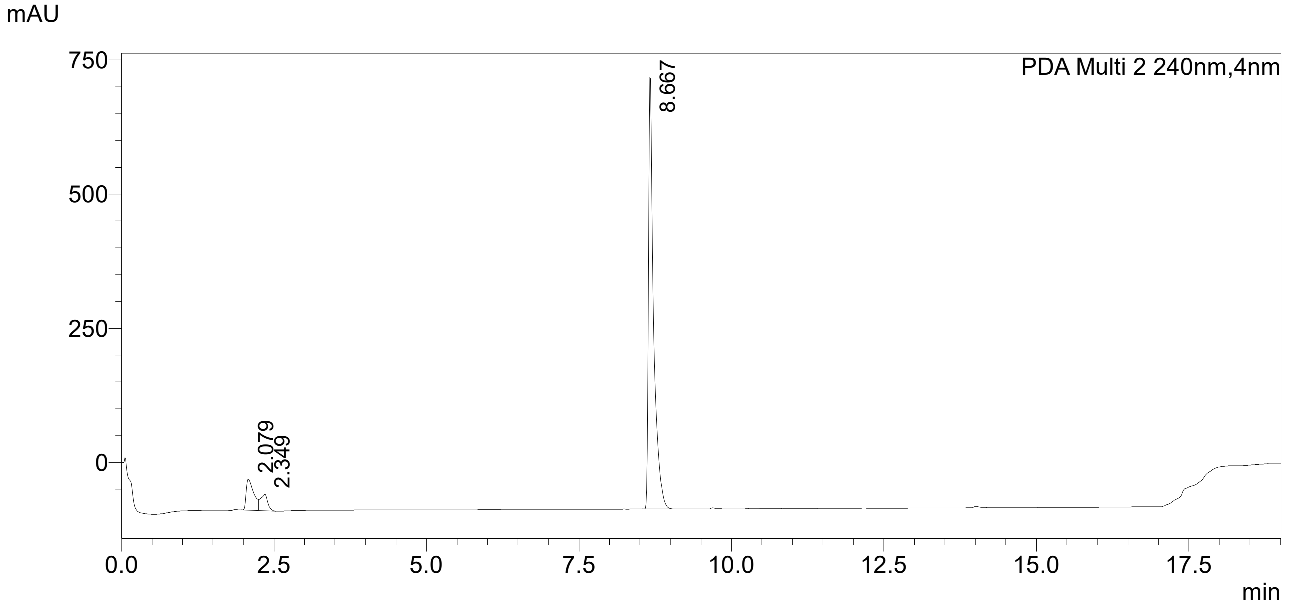

<Peak Table>

PDA Ch1 243nm

Peak#	Ret. Time	Area	Height	Conc.	Unit	Mark	Name
1	1.856	9504	1196	0.000			
2	2.074	233156	26757	0.000		V	
3	2.357	111893	14673	0.000		V	
4	8.667	4739134	793137	0.000			
5	9.694	12223	2137	-4.493	uM		MHET
6	13.867	3074	614	0.000			
7	14.012	19089	2725	0.000		V	
Total		5128073	841240				

PDA Ch2 240nm

Peak#	Ret. Time	Area	Height	Conc.	Unit	Mark	Name
1	2.079	519709	57805	0.000			
2	2.349	242945	30939	0.000		V	
3	8.667	4789150	804054	475.899	uM		TPA
Total		5551804	892798				
