# Supplementary material for: A flexible kinetic assay efficiently sorts prospective biocatalysts for PET plastic subunit hydrolysis
Source: RSC Adv. 2022 Mar 14;12(13):8119–30. doi: 10.1039/d2ra00612j (PMC8982334; doi:10.1039/d2ra00612j)
Supplement: RA-012-D2RA00612J-s009 [file RA-012-D2RA00612J-s009.pdf]

SHIMADZU LabSolutions

# Analysis Report

## <Sample Information>

| | | | |
|---|---|---|---|
| Sample Name | : E11 pH7 | | |
| Sample ID | : | | |
| Data Filename | : E11 pH7_027.lcd | | |
| Method Filename | : MHET_BHET_rpamide_060721.lcm | | |
| Batch Filename | : BHET_Colorimetric_37C_pH7_09072021.lcb | | |
| Vial # | : 3-11 | Sample Type | : Unknown |
| Injection Volume | : 10 uL | | |
| Date Acquired | : 9/7/2021 9:57:24 PM | Acquired by | : System Administrator |
| Date Processed | : 9/8/2021 10:30:12 AM | Processed by | : System Administrator |

## <Chromatogram>

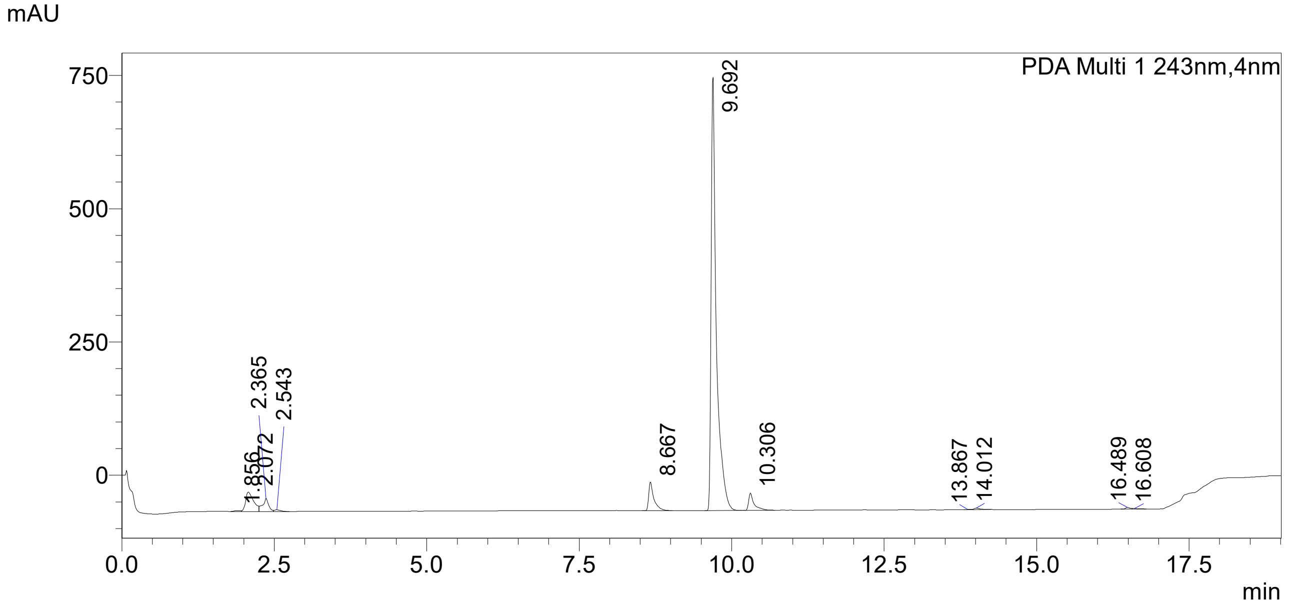


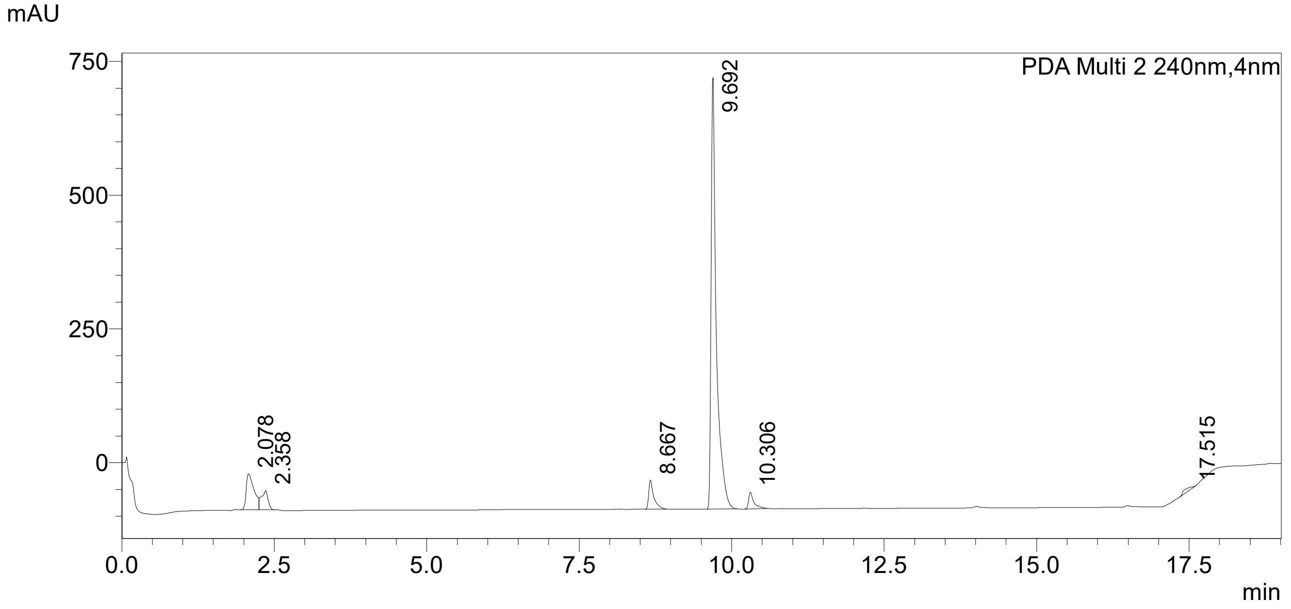


## <Peak Table>

PDA Ch1 243nm

| Peak# | Ret. Time | Area | Height | Conc. | Unit | Mark | Name |
|---|---|---|---|---|---|---|---|
| 1 | 1.856 | 12843 | 1427 | 0.000 | | | |
| 2 | 2.072 | 339485 | 36680 | 0.000 | | V | |
| 3 | 2.365 | 165467 | 24299 | 0.000 | | V | |
| 4 | 2.543 | 22136 | 3277 | 0.000 | | V | |
| 5 | 8.667 | 317461 | 53390 | 0.000 | | | |
| 6 | 9.692 | 4895040 | 812572 | 474.681 | uM | | MHET |
| 7 | 10.306 | 202341 | 32284 | 14.428 | uM | | BHET |
| 8 | 13.867 | 2702 | 525 | 0.000 | | | |
| 9 | 14.012 | 20474 | 2881 | 0.000 | | V | |
| 10 | 16.489 | 17018 | 3141 | 0.000 | | | |
| 11 | 16.608 | 7539 | 1166 | 0.000 | | V | |
| Total | | 6002506 | 971642 | | | | |

PDA Ch2 240nm

| Peak# | Ret. Time | Area | Height | Conc. | Unit | Mark | Name |
|---|---|---|---|---|---|---|---|
| 1 | 2.078 | 619593 | 67331 | 0.000 | | | |
| 2 | 2.358 | 269324 | 36292 | 0.000 | | V | |
| 3 | 8.667 | 315529 | 53723 | 25.066 | uM | | TPA |
| 4 | 9.692 | 4846599 | 806647 | 0.000 | | | |
| 5 | 10.306 | 185425 | 31077 | 0.000 | | | |
| 6 | 17.515 | 73401 | 4921 | 0.000 | | | |
| Total | | 6309871 | 999990 | | | | |
