# Supplementary material for: A flexible kinetic assay efficiently sorts prospective biocatalysts for PET plastic subunit hydrolysis
Source: RSC Adv. 2022 Mar 14;12(13):8119–30. doi: 10.1039/d2ra00612j (PMC8982334; doi:10.1039/d2ra00612j)
Supplement: RA-012-D2RA00612J-s010 [file RA-012-D2RA00612J-s010.pdf]

SHIMADZU LabSolutions

Analysis Report

<Sample Information>

Sample Name	: E2 pH7		
Sample ID	:		
Data Filename	: E2 pH7_040.lcd		
Method Filename	: MHET_BHET_rpamide_060721.lcm		
Batch Filename	: BHET_Colorimetric_37C_pH7_09072021.lcb		
Vial #	: 3-24	Sample Type	: Unknown
Injection Volume	: 10 uL		
Date Acquired	: 9/8/2021 2:08:49 AM	Acquired by	: System Administrator
Date Processed	: 9/8/2021 10:30:36 AM	Processed by	: System Administrator

<Chromatogram>

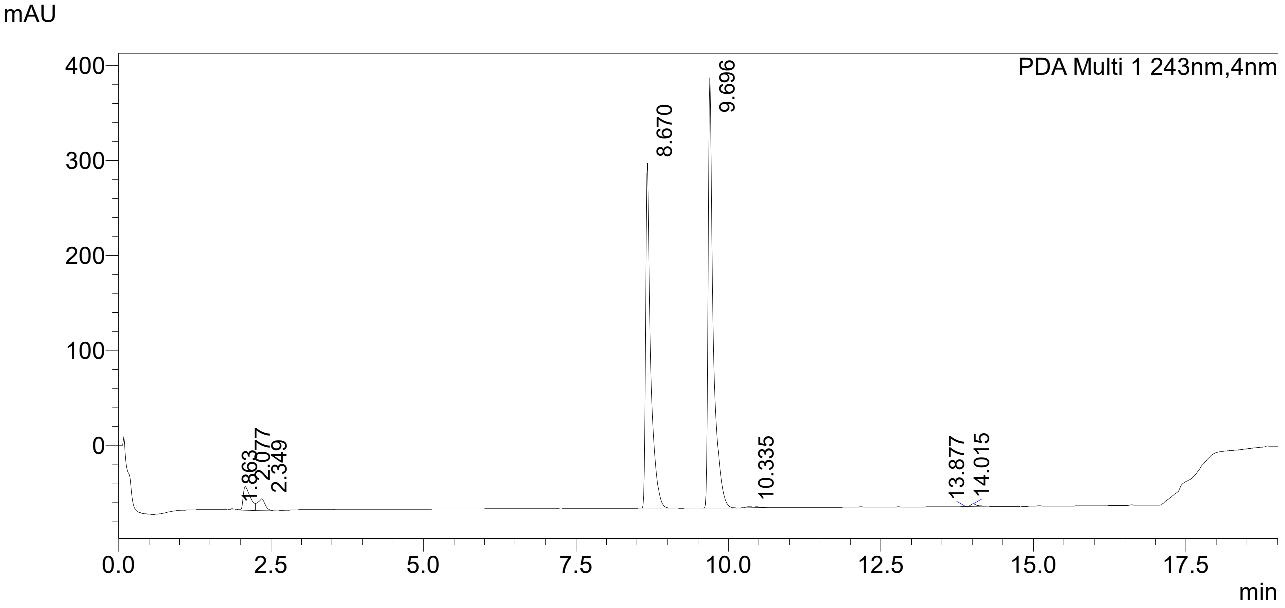

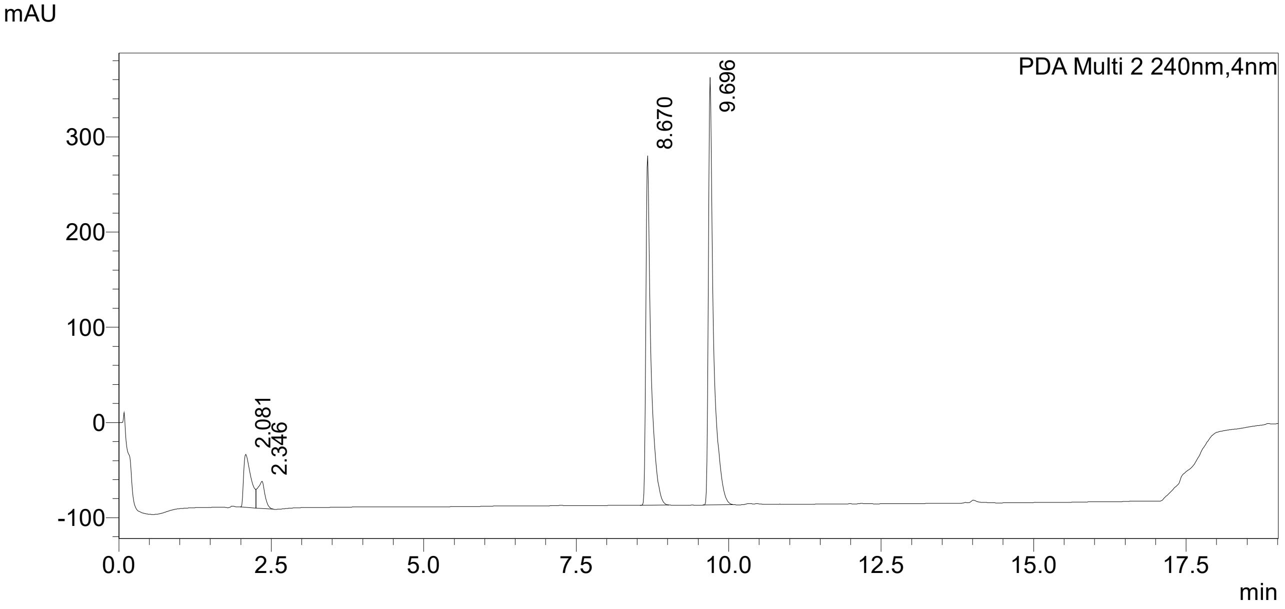

<Peak Table>

PDA Ch1 243nm

Peak#	Ret. Time	Area	Height	Conc.	Unit	Mark	Name
1	1.863	7738	1192	0.000			
2	2.077	212740	24733	0.000		V	
3	2.349	101314	12496	0.000		V	
4	8.670	2133850	363360	0.000			
5	9.696	2684692	453404	250.578	uM		MHET
6	10.335	14456	1093	-4.523	uM		BHET
7	13.877	2906	645	0.000			
8	14.015	19463	2748	0.000		V	
Total		5177159	859670				

PDA Ch2 240nm

Peak#	Ret. Time	Area	Height	Conc.	Unit	Mark	Name
1	2.081	491550	55616	0.000			
2	2.346	227943	28468	0.000		V	
3	8.670	2149632	366878	204.100	uM		TPA
4	9.696	2651310	448941	0.000			
Total		5520435	899903				
