# Supplementary material for: A flexible kinetic assay efficiently sorts prospective biocatalysts for PET plastic subunit hydrolysis
Source: RSC Adv. 2022 Mar 14;12(13):8119–30. doi: 10.1039/d2ra00612j (PMC8982334; doi:10.1039/d2ra00612j)
Supplement: RA-012-D2RA00612J-s011 [file RA-012-D2RA00612J-s011.pdf]

SHIMADZU LabSolutions

Analysis Report

<Sample Information>

Sample Name	: E1		
Sample ID	:		
Data Filename	: E1 _006.lcd		
Method Filename	: MHET_BHET_rpamide_060721.lcm		
Batch Filename	: BHET_Colorimetric_37C_pH8_plate1_RECALC.lcb		
Vial #	: 4-19	Sample Type	: Unknown
Injection Volume	: 10 uL		
Date Acquired	: 8/24/2021 9:39:39 AM	Acquired by	: System Administrator
Date Processed	: 9/3/2021 9:16:19 AM	Processed by	: System Administrator

<Chromatogram>

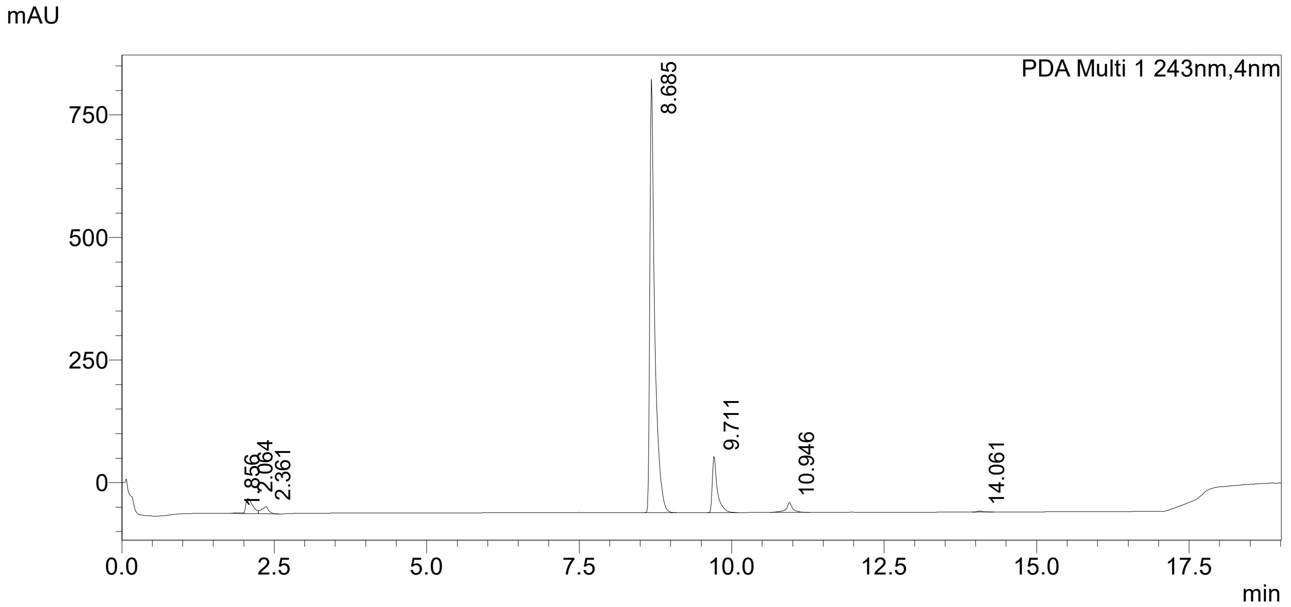

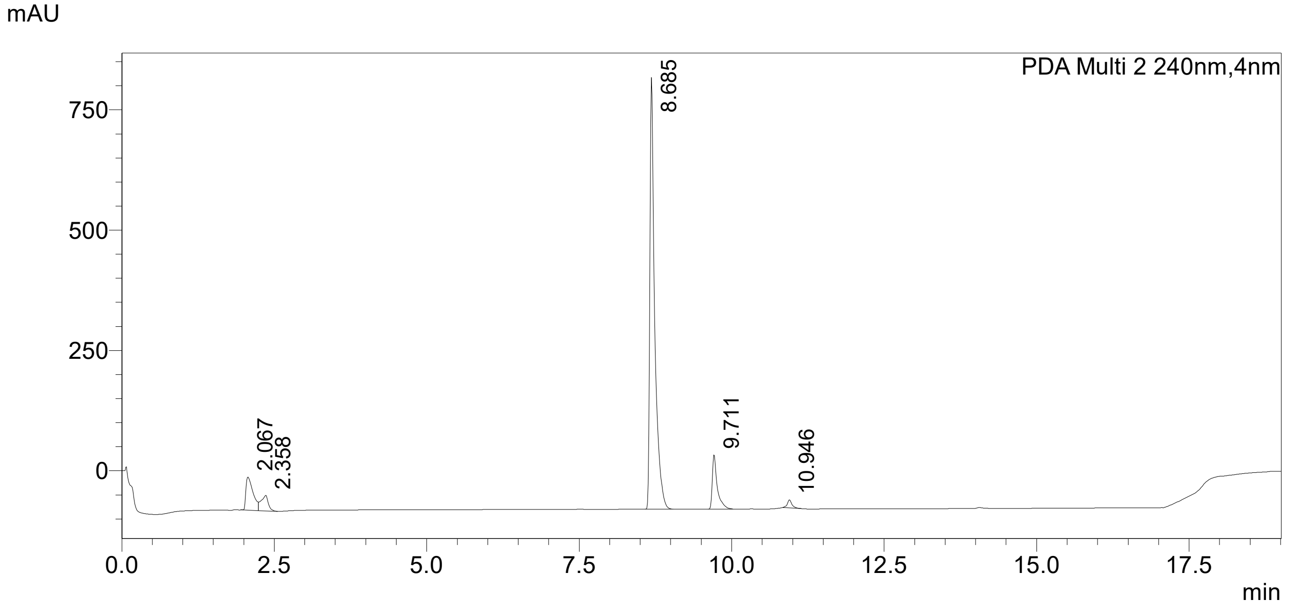

<Peak Table>

PDA Ch1 243nm

Peak#	Ret. Time	Area	Height	Conc.	Unit	Mark	Name
1	1.856	11012	1174	0.000			
2	2.064	248544	30930	0.000		V	
3	2.361	115377	14667	0.000		V	
4	8.685	4993544	883628	0.000			
5	9.711	656641	113940	57.341	uM		MHET
6	10.946	151375	20672	0.000			
7	14.061	14113	1982	0.000			
Total		6190606	1066993				

PDA Ch2 240nm

Peak#	Ret. Time	Area	Height	Conc.	Unit	Mark	Name
1	2.067	575060	68773	0.000			
2	2.358	259428	32504	0.000		V	
3	8.685	5051996	896992	487.791	uM		TPA
4	9.711	644336	112610	0.000			
5	10.946	87262	16596	0.000			
Total		6618083	1127475				
