# Supplementary material for: A flexible kinetic assay efficiently sorts prospective biocatalysts for PET plastic subunit hydrolysis
Source: RSC Adv. 2022 Mar 14;12(13):8119–30. doi: 10.1039/d2ra00612j (PMC8982334; doi:10.1039/d2ra00612j)
Supplement: RA-012-D2RA00612J-s012 [file RA-012-D2RA00612J-s012.pdf]

SHIMADZU LabSolutions

Analysis Report

<Sample Information>

Sample Name	E10		
Sample ID	:		
Data Filename	: E10 _015.lcd		
Method Filename	: MHET_BHET_rpamide_060721.lcm		
Batch Filename	: BHET_Colorimetric_37C_pH8_plate1.lcb		
Vial #	: 3-9	Sample Type	: Unknown
Injection Volume	: 10 uL		
Date Acquired	: 8/24/2021 11:33:24 PM	Acquired by	: System Administrator
Date Processed	: 9/3/2021 9:07:02 AM	Processed by	: System Administrator

<Chromatogram>

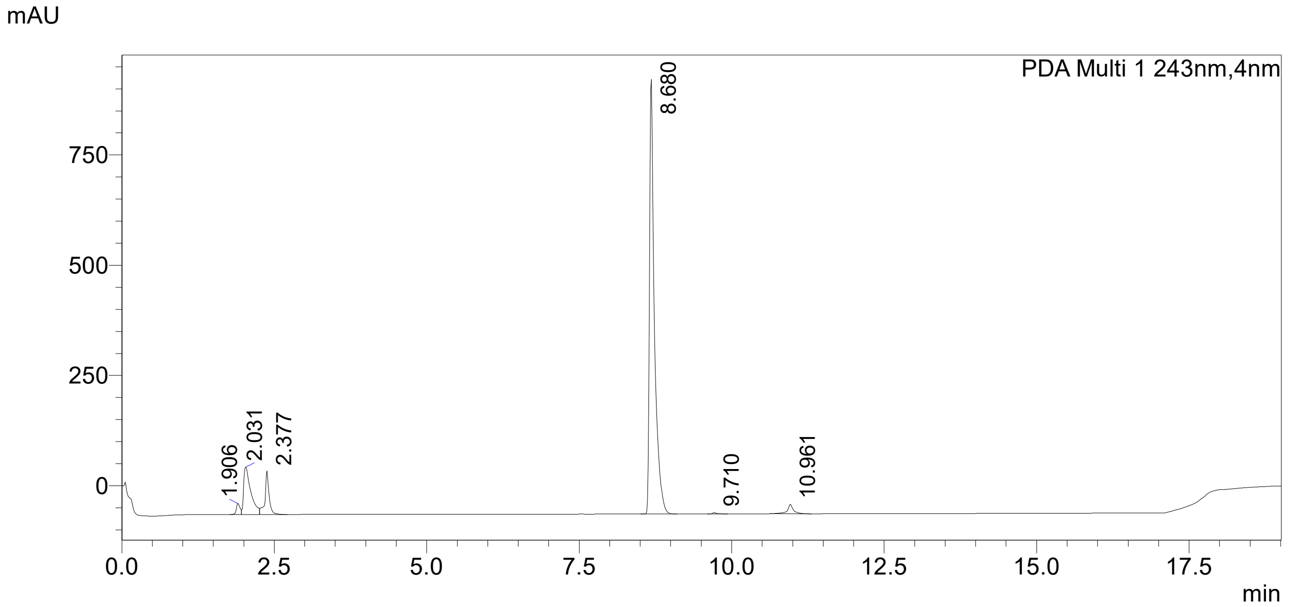

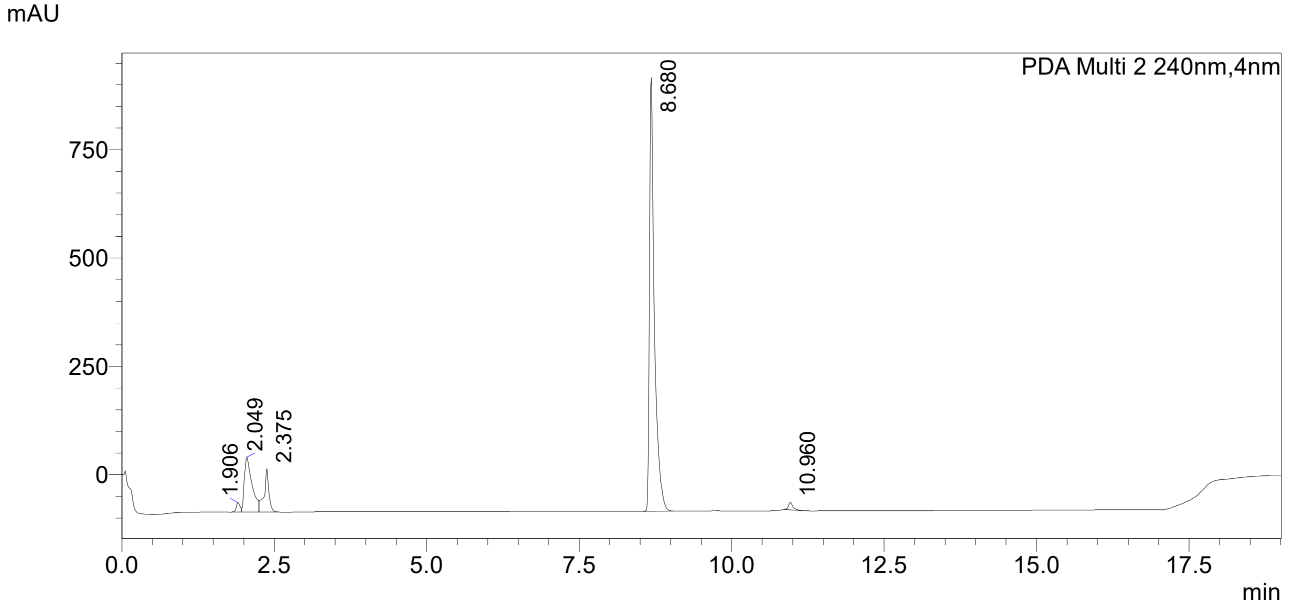

<Peak Table>

PDA Ch1 243nm

Peak#	Ret. Time	Area	Height	Conc.	Unit	Mark	Name
1	1.906	111829	25111	0.000			
2	2.031	900993	107641	0.000		V	
3	2.377	464664	98229	0.000		V	
4	8.680	5623676	985323	0.000			
5	9.710	15164	2665	-1.687	uM		MHET
6	10.961	151676	21082	0.000			
Total		7268002	1240052				

PDA Ch2 240nm

Peak#	Ret. Time	Area	Height	Conc.	Unit	Mark	Name
1	1.906	97256	21757	0.000			
2	2.049	1135302	126147	0.000		V	
3	2.375	547999	98906	0.000		V	
4	8.680	5694606	1001225	560.884	uM		TPA
5	10.960	87036	16874	0.000			
Total		7562198	1264909				
