# Supplementary material for: A flexible kinetic assay efficiently sorts prospective biocatalysts for PET plastic subunit hydrolysis
Source: RSC Adv. 2022 Mar 14;12(13):8119–30. doi: 10.1039/d2ra00612j (PMC8982334; doi:10.1039/d2ra00612j)
Supplement: RA-012-D2RA00612J-s013 [file RA-012-D2RA00612J-s013.pdf]

SHIMADZU LabSolutions

Analysis Report

<Sample Information>

Sample Name	: Bacillis subtilis Plate 1		
Sample ID	:		
Data Filename	: Bacillis subtilis Plate 1_017.lcd		
Method Filename	: MHET_BHET_rpamide_060721.lcm		
Batch Filename	: BHET_Colorimetric_37C_pH8_plate1.lcb		
Vial #	: 3-11	Sample Type	: Unknown
Injection Volume	: 10 uL		
Date Acquired	: 8/25/2021 12:12:05 AM	Acquired by	: System Administrator
Date Processed	: 9/3/2021 9:07:06 AM	Processed by	: System Administrator

<Chromatogram>

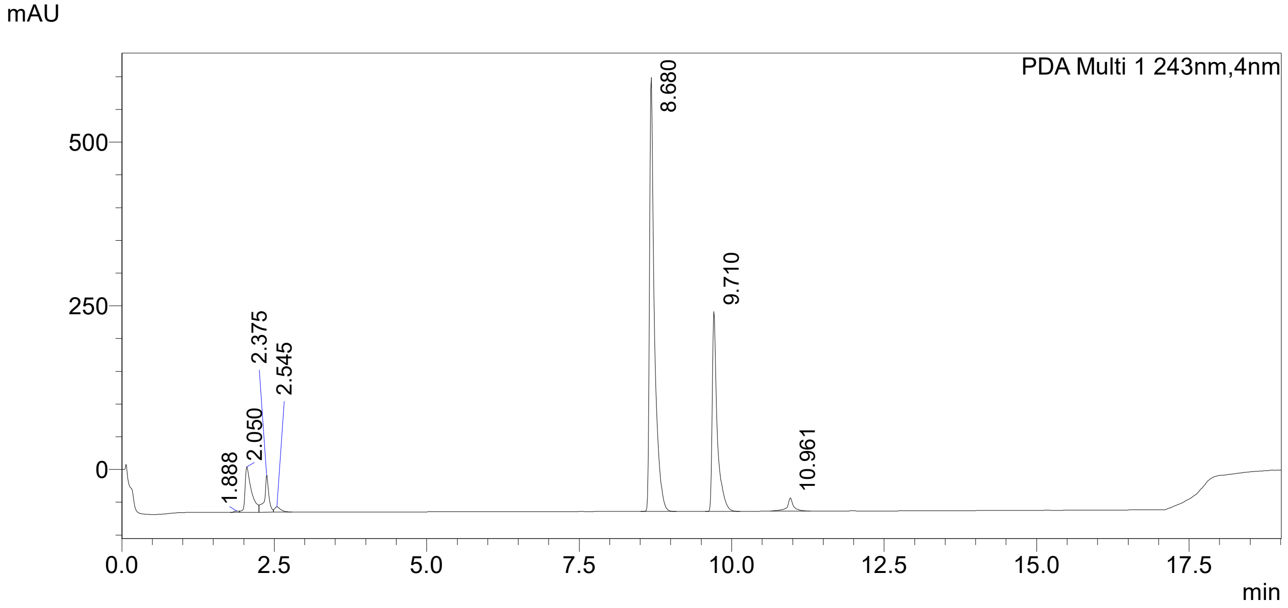

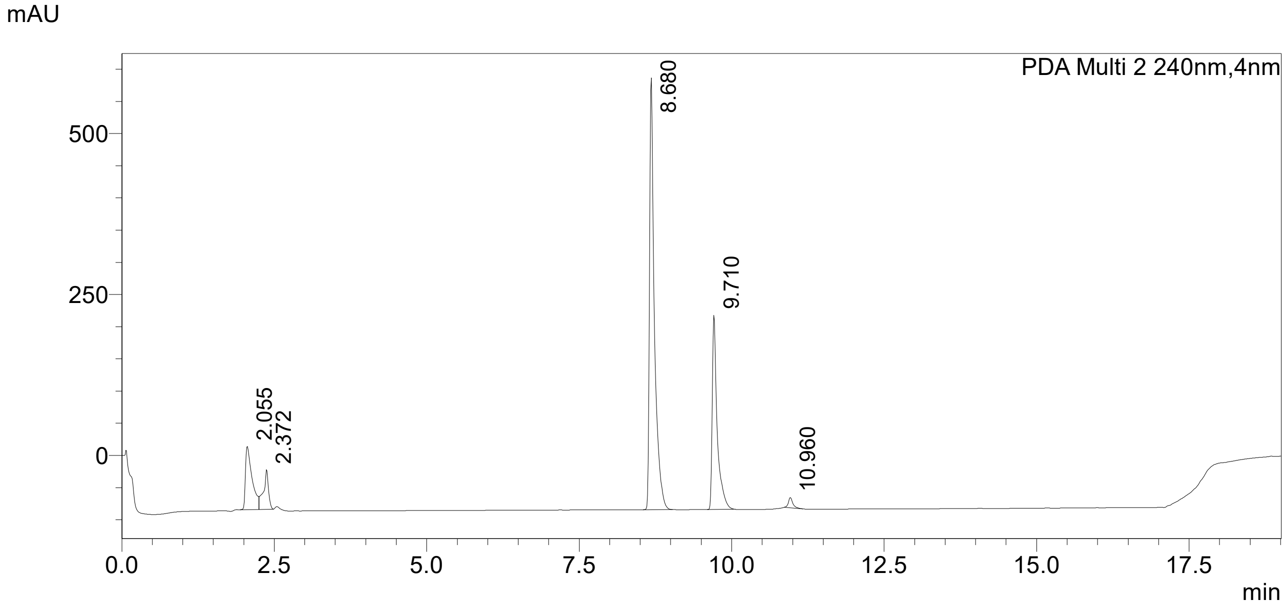

<Peak Table>

PDA Ch1 243nm

Peak#	Ret. Time	Area	Height	Conc.	Unit	Mark	Name
1	1.888	11997	2071	0.000			
2	2.050	517541	69300	0.000		V	
3	2.375	282842	56017	0.000		V	
4	2.545	54869	8132	0.000		V	
5	8.680	3761649	662883	0.000			
6	9.710	1748690	305326	161.551	uM		MHET
7	10.961	142008	19866	0.000			
Total		6519594	1123594				

PDA Ch2 240nm

Peak#	Ret. Time	Area	Height	Conc.	Unit	Mark	Name
1	2.055	769822	98392	0.000			
2	2.372	350030	61465	0.000		V	
3	8.680	3798618	670839	367.001	uM		TPA
4	9.710	1725703	302101	0.000			
5	10.960	82111	15927	0.000			
Total		6726283	1148724				
