# Supplementary material for: A flexible kinetic assay efficiently sorts prospective biocatalysts for PET plastic subunit hydrolysis
Source: RSC Adv. 2022 Mar 14;12(13):8119–30. doi: 10.1039/d2ra00612j (PMC8982334; doi:10.1039/d2ra00612j)
Supplement: RA-012-D2RA00612J-s014 [file RA-012-D2RA00612J-s014.pdf]

SHIMADZU LabSolutions

Analysis Report

<Sample Information>

Sample Name	: E12		
Sample ID	:		
Data Filename	: E12 _013.lcd		
Method Filename	: MHET_BHET_rpamide_060721.lcm		
Batch Filename	: BHET_Colorimetric_37C_pH8_plate1_Commercials.lcb		
Vial #	: 3-6	Sample Type	: Unknown
Injection Volume	: 10 uL		
Date Acquired	: 8/25/2021 11:10:58 AM	Acquired by	: System Administrator
Date Processed	: 9/3/2021 9:02:37 AM	Processed by	: System Administrator

<Chromatogram>

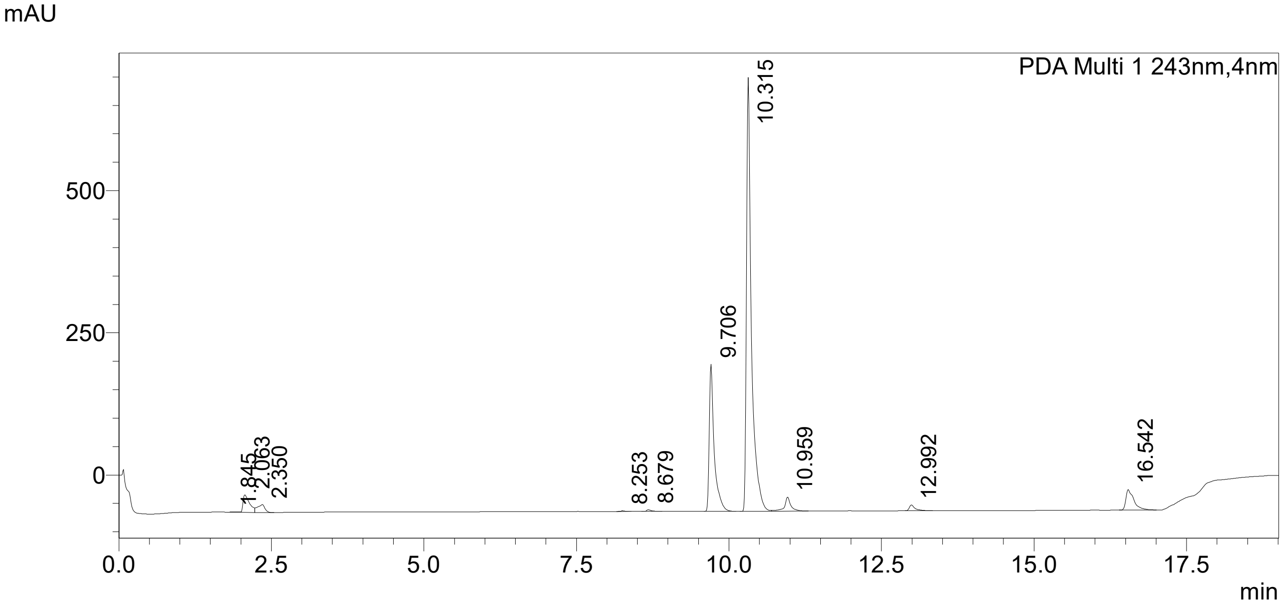

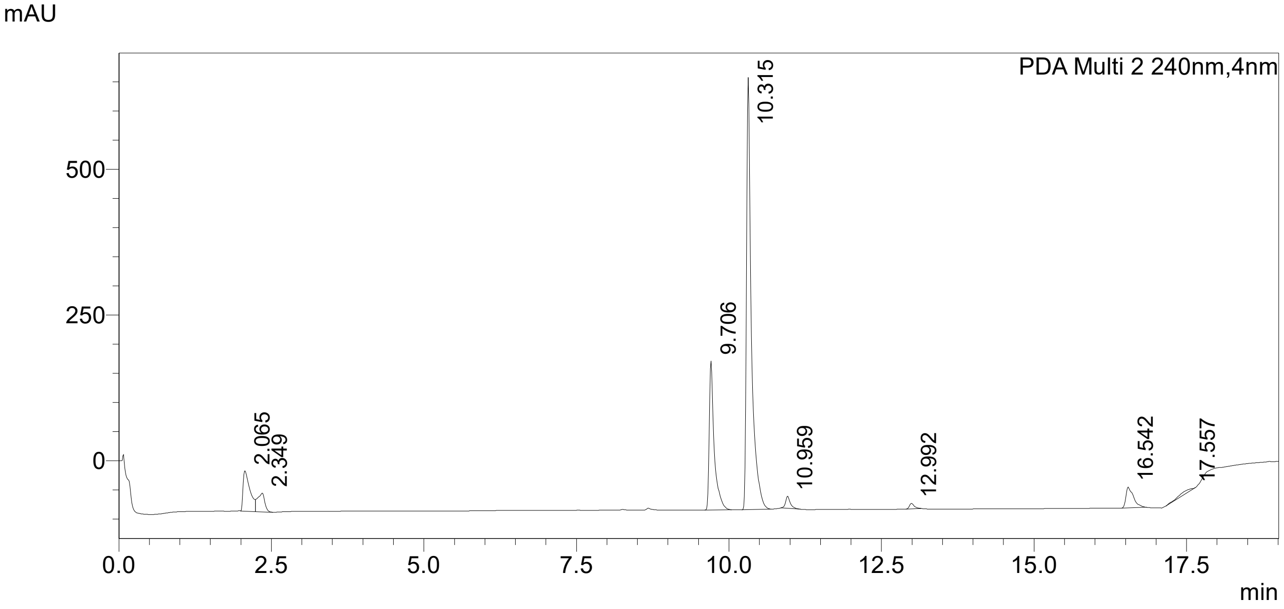

<Peak Table>

PDA Ch1 243nm

Peak#	Ret. Time	Area	Height	Conc.	Unit	Mark	Name
1	1.845	7719	637	0.000			
2	2.063	236320	30727	0.000		V	
3	2.350	118078	14185	0.000		V	
4	8.253	5595	1188	0.000			
5	8.679	16832	3036	0.000			
6	9.706	1473190	258768	133.326	uM		MHET
7	10.315	4362271	762960	427.132	uM		BHET
8	10.959	181085	24790	0.000		V	
9	12.992	69454	10355	0.000			
10	16.542	326557	35933	0.000			
Total		6797102	1142579				

PDA Ch2 240nm

Peak#	Ret. Time	Area	Height	Conc.	Unit	Mark	Name
1	2.065	564150	68798	0.000			
2	2.349	256947	32069	0.000		V	
3	9.706	1452684	255954	0.000			
4	10.315	4217804	741330	0.000			
5	10.959	112272	20503	0.000			
6	12.992	54072	9655	0.000			
7	16.542	307674	35677	0.000			
8	17.557	86673	3254	0.000			
Total		7052275	1167238				
