# Supplementary material for: A flexible kinetic assay efficiently sorts prospective biocatalysts for PET plastic subunit hydrolysis
Source: RSC Adv. 2022 Mar 14;12(13):8119–30. doi: 10.1039/d2ra00612j (PMC8982334; doi:10.1039/d2ra00612j)
Supplement: RA-012-D2RA00612J-s015 [file RA-012-D2RA00612J-s015.pdf]

SHIMADZU LabSolutions

Analysis Report

<Sample Information>

Sample Name	: E13		
Sample ID	:		
Data Filename	: E13_016.lcd		
Method Filename	: MHET_BHET_rpamide_060721.lcm		
Batch Filename	: BHET_Colorimetric_37C_pH8_plate1_Commercials.lcb		
Vial #	: 3-9	Sample Type	: Unknown
Injection Volume	: 10 uL		
Date Acquired	: 8/25/2021 12:09:02 PM	Acquired by	: System Administrator
Date Processed	: 9/3/2021 9:02:42 AM	Processed by	: System Administrator

<Chromatogram>

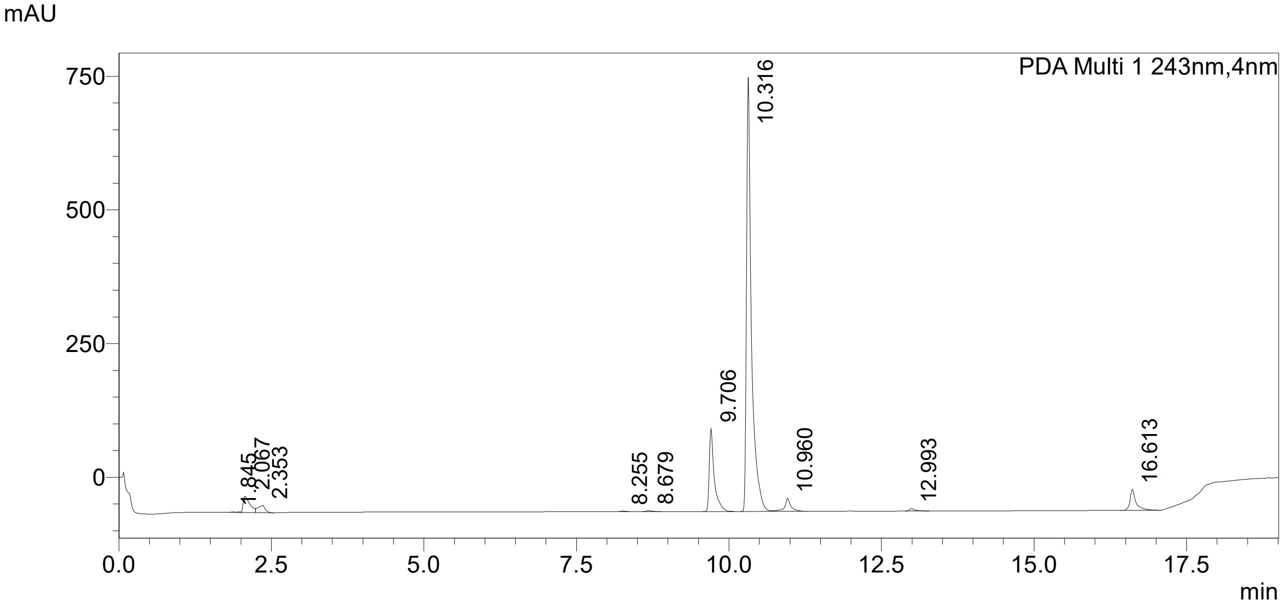

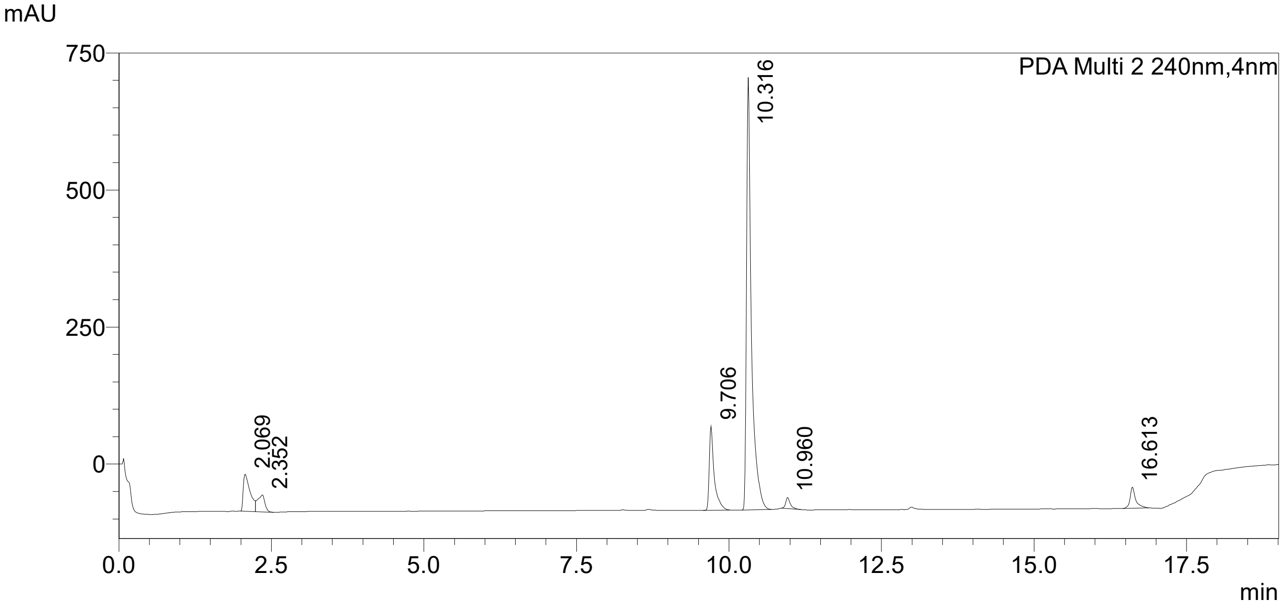

<Peak Table>

PDA Ch1 243nm

Peak#	Ret. Time	Area	Height	Conc.	Unit	Mark	Name
1	1.845	8122	623	0.000			
2	2.067	234653	30223	0.000		V	
3	2.353	110492	13660	0.000		V	
4	8.255	4743	1143	0.000			
5	8.679	10353	1840	0.000			
6	9.706	881757	154810	78.055	uM		MHET
7	10.316	4645533	811676	454.090	uM		BHET
8	10.960	178363	24274	0.000		V	
9	12.993	30926	4628	0.000			
10	16.613	271561	39003	0.000			
Total		6376503	1081881				

PDA Ch2 240nm

Peak#	Ret. Time	Area	Height	Conc.	Unit	Mark	Name
1	2.069	547343	67708	0.000			
2	2.352	250976	30727	0.000		V	
3	9.706	866643	153015	0.000			
4	10.316	4493305	788949	0.000			
5	10.960	111085	20116	0.000			
6	16.613	247441	38196	0.000			
Total		6516794	1098711				
