# Supplementary material for: A flexible kinetic assay efficiently sorts prospective biocatalysts for PET plastic subunit hydrolysis
Source: RSC Adv. 2022 Mar 14;12(13):8119–30. doi: 10.1039/d2ra00612j (PMC8982334; doi:10.1039/d2ra00612j)
Supplement: RA-012-D2RA00612J-s016 [file RA-012-D2RA00612J-s016.pdf]

SHIMADZU LabSolutions

Analysis Report

<Sample Information>

Sample Name	: E14		
Sample ID	:		
Data Filename	: E14_018.lcd		
Method Filename	: MHET_BHET_rpamide_060721.lcm		
Batch Filename	: BHET_Colorimetric_37C_pH8_plate1_Commercials.lcb		
Vial #	: 3-11	Sample Type	: Unknown
Injection Volume	: 10 uL		
Date Acquired	: 8/25/2021 12:47:43 PM	Acquired by	: System Administrator
Date Processed	: 9/3/2021 9:02:46 AM	Processed by	: System Administrator

<Chromatogram>

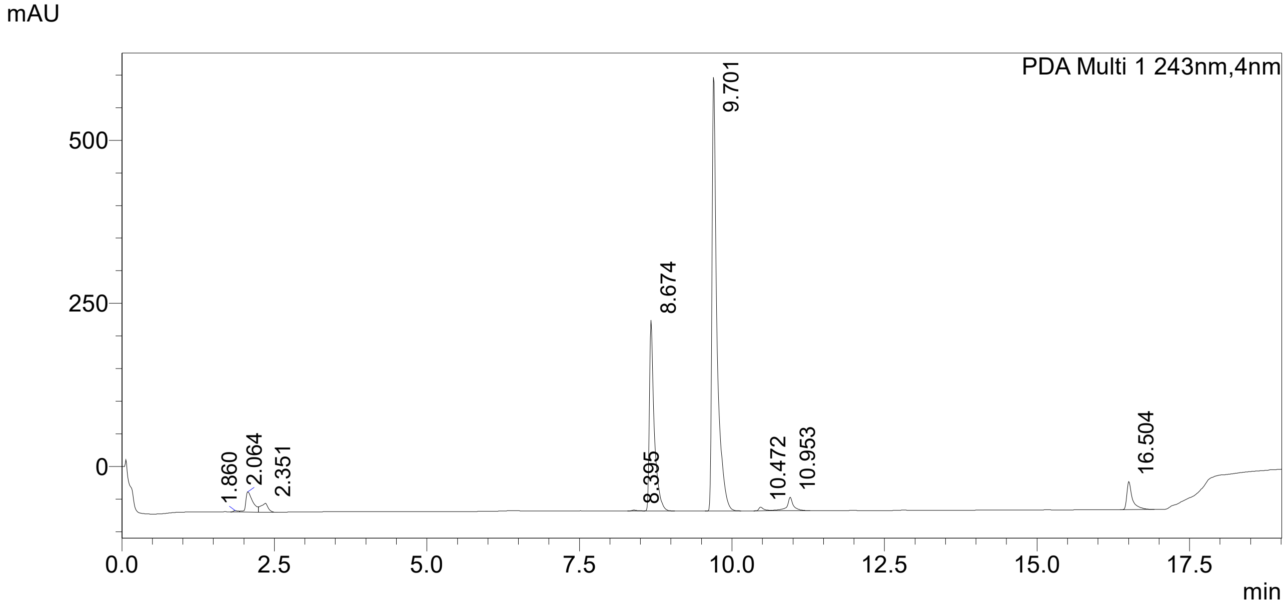

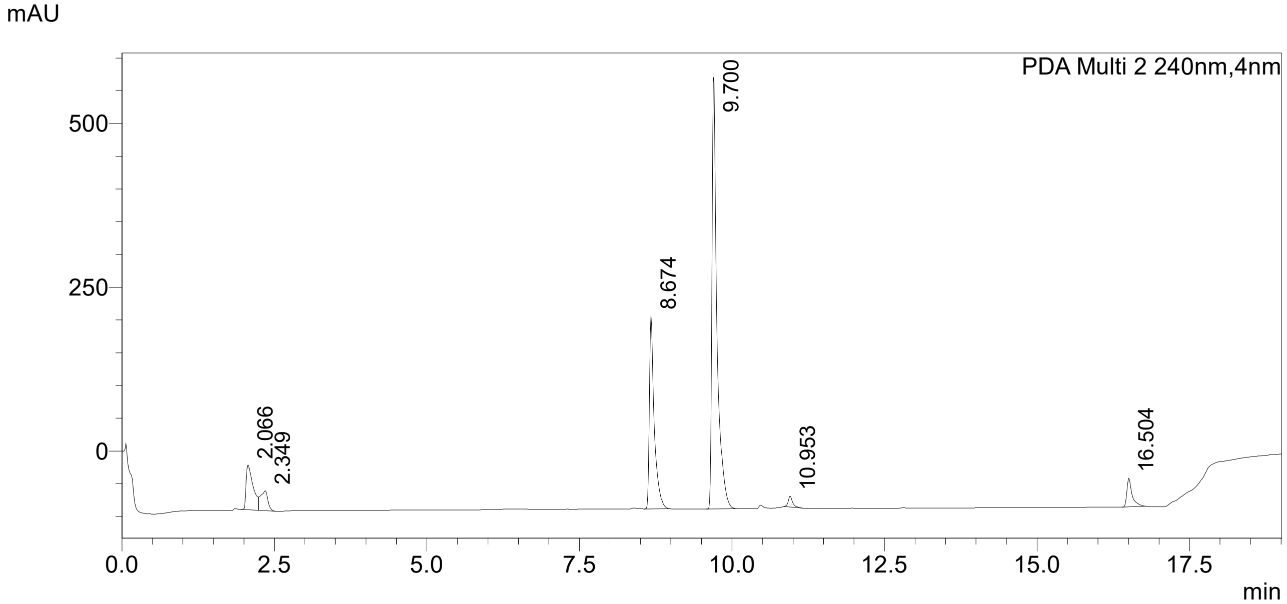

<Peak Table>

PDA Ch1 243nm

Peak#	Ret. Time	Area	Height	Conc.	Unit	Mark	Name
1	1.860	12257	2150	0.000			
2	2.064	256756	31040	0.000		V	
3	2.351	112882	13578	0.000		V	
4	8.395	8274	1286	0.000			
5	8.674	1643709	292321	0.000		V	
6	9.701	3849508	664515	364.631	uM		MHET
7	10.472	32686	5586	0.428	uM		BHET
8	10.953	153222	20781	0.000		V	
9	16.504	273325	43007	0.000			
Total		6342618	1074265				

PDA Ch2 240nm

Peak#	Ret. Time	Area	Height	Conc.	Unit	Mark	Name
1	2.066	563821	67870	0.000			
2	2.349	249159	30425	0.000		V	
3	8.674	1650984	294779	154.135	uM		TPA
4	9.700	3808221	658888	0.000			
5	10.953	85554	16633	0.000			
6	16.504	263937	43300	0.000			
Total		6621675	1111896				
