# Supplementary material for: A flexible kinetic assay efficiently sorts prospective biocatalysts for PET plastic subunit hydrolysis
Source: RSC Adv. 2022 Mar 14;12(13):8119–30. doi: 10.1039/d2ra00612j (PMC8982334; doi:10.1039/d2ra00612j)
Supplement: RA-012-D2RA00612J-s017 [file RA-012-D2RA00612J-s017.pdf]

SHIMADZU LabSolutions

Analysis Report

<Sample Information>

Sample Name	: E15		
Sample ID	:		
Data Filename	: E15_025.lcd		
Method Filename	: MHET_BHET_rpamide_060721.lcm		
Batch Filename	: BHET_Colorimetric_37C_pH8_plate1_Commercials.lcb		
Vial #	: 3-18	Sample Type	: Unknown
Injection Volume	: 10 uL		
Date Acquired	: 8/25/2021 3:03:08 PM	Acquired by	: System Administrator
Date Processed	: 9/3/2021 9:02:59 AM	Processed by	: System Administrator

<Chromatogram>

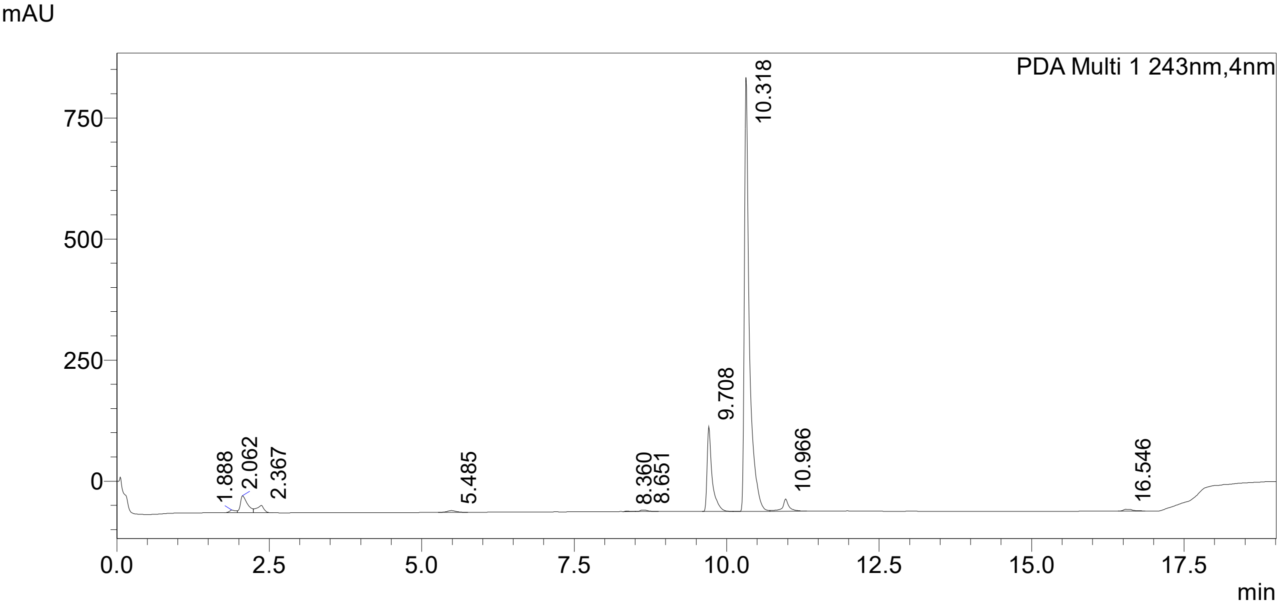

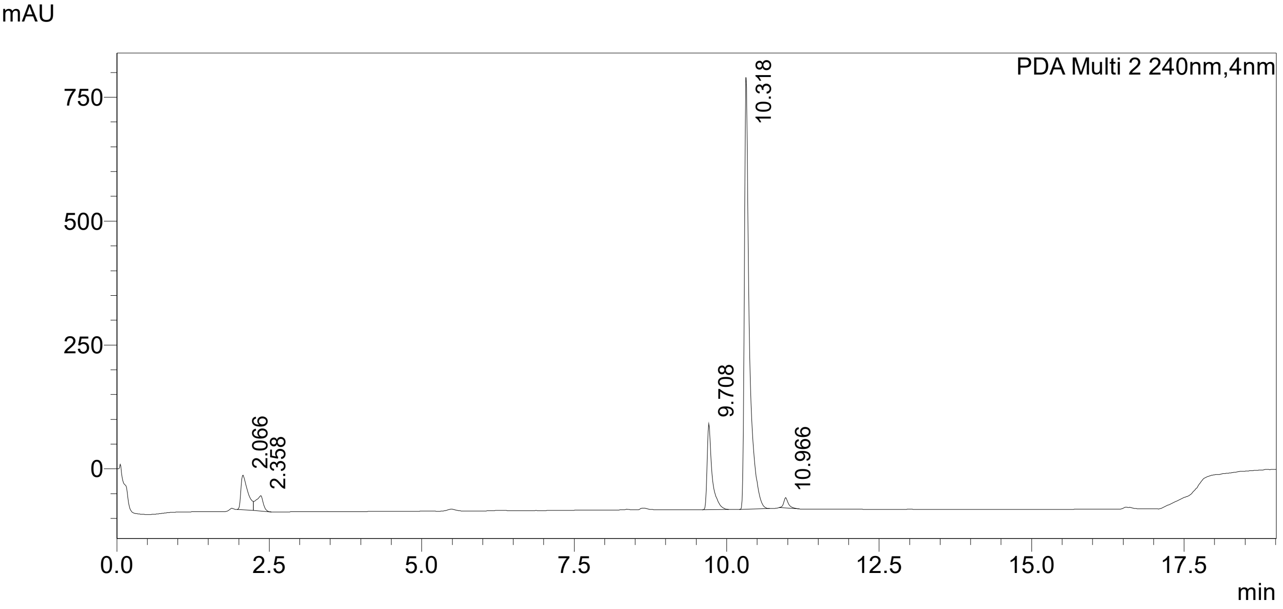

<Peak Table>

PDA Ch1 243nm

Peak#	Ret. Time	Area	Height	Conc.	Unit	Mark	Name
1	1.888	39689	5505	0.000			
2	2.062	288949	35147	0.000		V	
3	2.367	124531	15159	0.000		V	
4	5.485	39991	3806	0.000			
5	8.360	6483	1349	0.000			
6	8.651	25905	2966	0.000		V	
7	9.708	1002716	175311	89.186	uM		MHET
8	10.318	5185030	895873	506.691	uM	V	BHET
9	10.966	184074	25296	0.000		V	
10	16.546	37725	3655	0.000			
Total		6935095	1164067				

PDA Ch2 240nm

Peak#	Ret. Time	Area	Height	Conc.	Unit	Mark	Name
1	2.066	566557	69522	0.000			
2	2.358	254811	31138	0.000		V	
3	9.708	986411	173289	0.000			
4	10.318	5016353	871529	0.000			
5	10.966	111578	20840	0.000			
Total		6935709	1166318				
