# Supplementary material for: A flexible kinetic assay efficiently sorts prospective biocatalysts for PET plastic subunit hydrolysis
Source: RSC Adv. 2022 Mar 14;12(13):8119–30. doi: 10.1039/d2ra00612j (PMC8982334; doi:10.1039/d2ra00612j)
Supplement: RA-012-D2RA00612J-s018 [file RA-012-D2RA00612J-s018.pdf]

SHIMADZU LabSolutions

Analysis Report

<Sample Information>

Sample Name	: E16		
Sample ID	:		
Data Filename	: E16_031.lcd		
Method Filename	: MHET_BHET_rpamide_060721.lcm		
Batch Filename	: BHET_Colorimetric_37C_pH8_plate1_Commercials.lcb		
Vial #	: 3-24	Sample Type	: Unknown
Injection Volume	: 10 uL		
Date Acquired	: 8/25/2021 4:59:10 PM	Acquired by	: System Administrator
Date Processed	: 9/3/2021 9:03:10 AM	Processed by	: System Administrator

<Chromatogram>

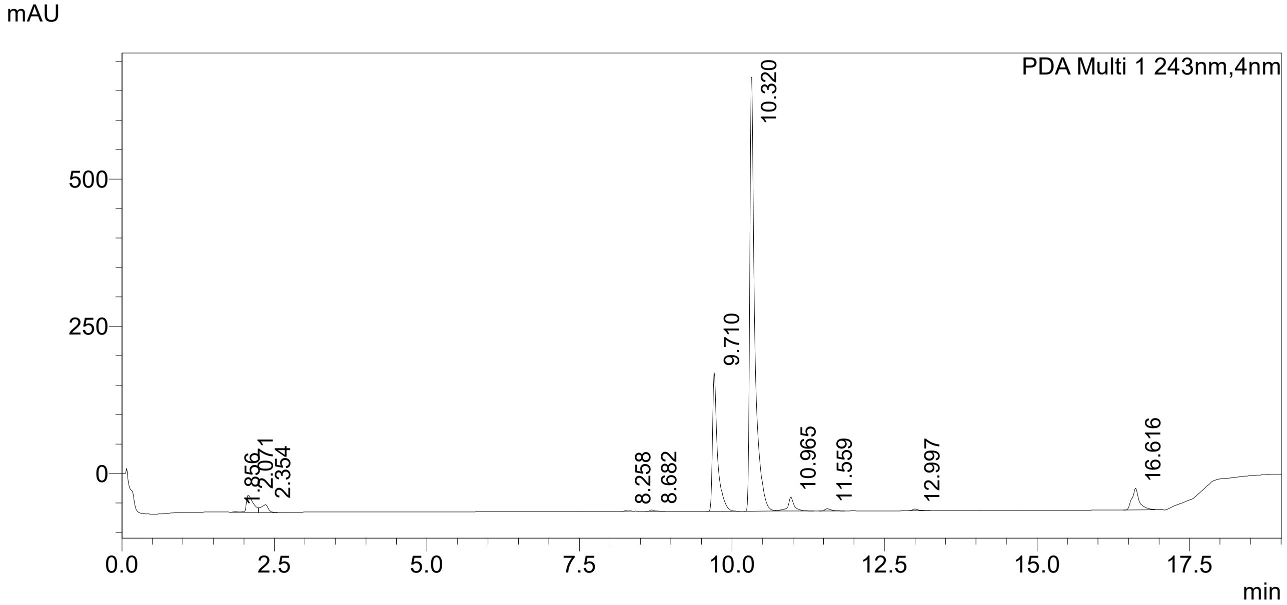

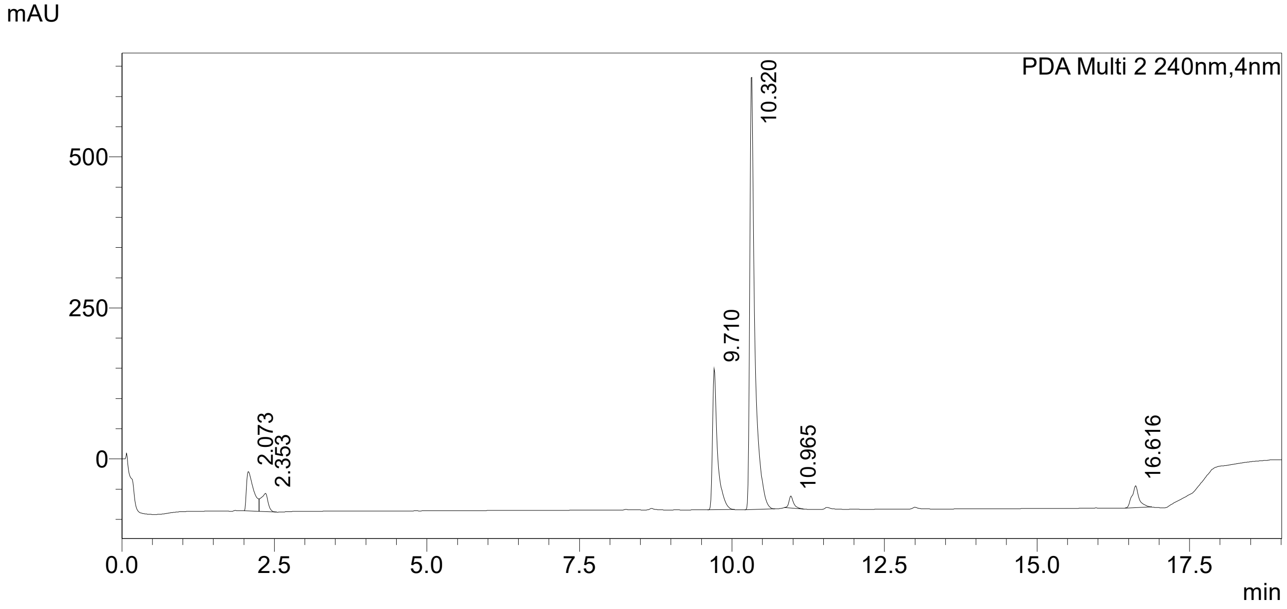

<Peak Table>

PDA Ch1 243nm

Peak#	Ret. Time	Area	Height	Conc.	Unit	Mark	Name
1	1.856	9323	971	0.000			
2	2.071	231639	29207	0.000		V	
3	2.354	111009	13483	0.000		V	
4	8.258	3605	962	0.000			
5	8.682	13327	2415	0.000			
6	9.710	1358657	236295	121.939	uM		MHET
7	10.320	4305962	736291	421.773	uM		BHET
8	10.965	175642	24088	0.000		V	
9	11.559	28161	3908	0.000			
10	12.997	19807	3035	0.000			
11	16.616	314900	37021	0.000			
Total		6572033	1087675				

PDA Ch2 240nm

Peak#	Ret. Time	Area	Height	Conc.	Unit	Mark	Name
1	2.073	553715	65403	0.000			
2	2.353	239504	30366	0.000		V	
3	9.710	1338443	233677	0.000			
4	10.320	4163018	715240	0.000			
5	10.965	107187	19830	0.000			
6	16.616	292550	36262	0.000			
Total		6694417	1100779				
