# Supplementary material for: A flexible kinetic assay efficiently sorts prospective biocatalysts for PET plastic subunit hydrolysis
Source: RSC Adv. 2022 Mar 14;12(13):8119–30. doi: 10.1039/d2ra00612j (PMC8982334; doi:10.1039/d2ra00612j)
Supplement: RA-012-D2RA00612J-s019 [file RA-012-D2RA00612J-s019.pdf]

Analysis Report

SHIMADZU LabSolutions

<Sample Information>

Sample Name	: E17		
Sample ID	:		
Data Filename	: E17_034.lcd		
Method Filename	: MHET_BHET_rpamide_060721.lcm		
Batch Filename	: BHET_Colorimetric_37C_pH8_plate1_Commercials.lcb		
Vial #	: 3-27	Sample Type	: Unknown
Injection Volume	: 10 uL		
Date Acquired	: 8/25/2021 5:57:12 PM	Acquired by	: System Administrator
Date Processed	: 9/3/2021 9:03:15 AM	Processed by	: System Administrator

<Chromatogram>

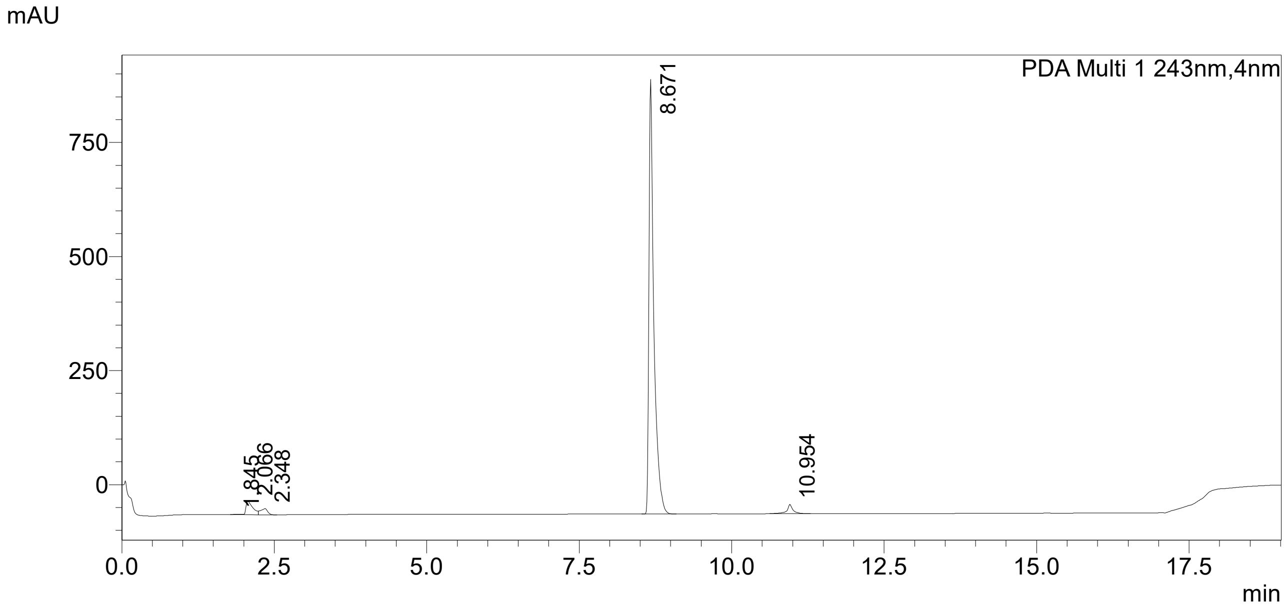

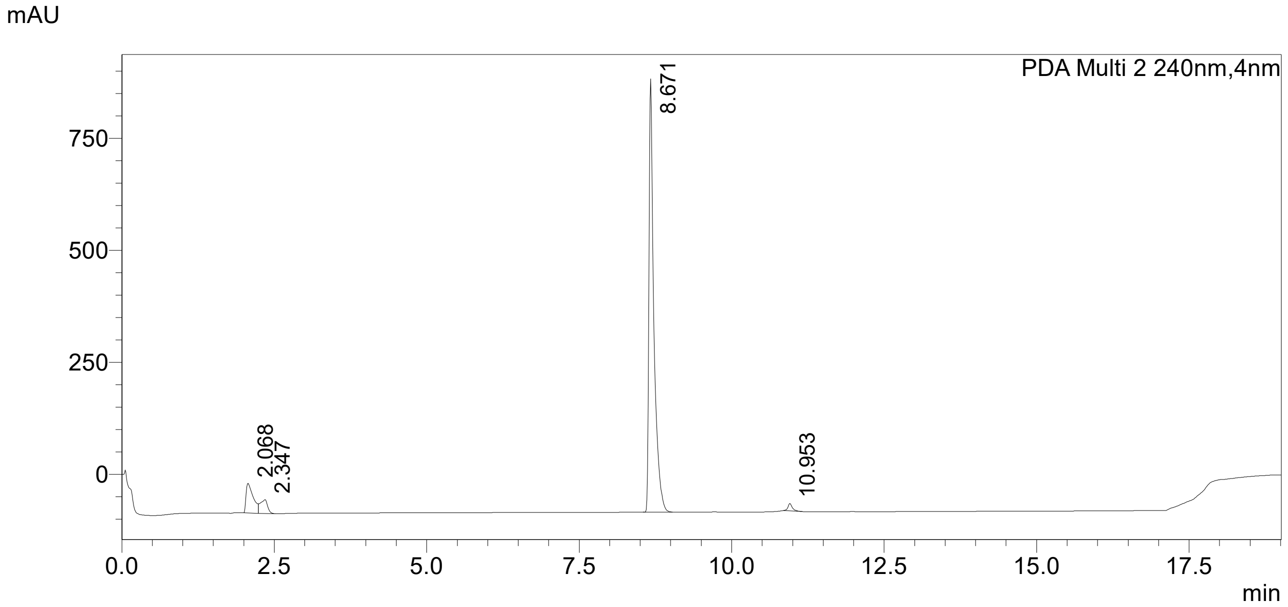

<Peak Table>

PDA Ch1 243nm

Peak#	Ret. Time	Area	Height	Conc.	Unit	Mark	Name
1	1.845	8822	794	0.000			
2	2.066	238025	29370	0.000		V	
3	2.348	110668	13798	0.000		V	
4	8.671	5431477	952331	0.000			
5	10.954	144209	20218	0.000			
Total		5933201	1016512				

PDA Ch2 240nm

Peak#	Ret. Time	Area	Height	Conc.	Unit	Mark	Name
1	2.068	554609	66020	0.000			
2	2.347	251214	31138	0.000		V	
3	8.671	5496723	967049	537.532	uM		TPA
4	10.953	84108	16308	0.000			
Total		6386655	1080515				
