# Supplementary material for: A flexible kinetic assay efficiently sorts prospective biocatalysts for PET plastic subunit hydrolysis
Source: RSC Adv. 2022 Mar 14;12(13):8119–30. doi: 10.1039/d2ra00612j (PMC8982334; doi:10.1039/d2ra00612j)
Supplement: RA-012-D2RA00612J-s020 [file RA-012-D2RA00612J-s020.pdf]

SHIMADZU LabSolutions

Analysis Report

<Sample Information>

Sample Name	: E18		
Sample ID	:		
Data Filename	: E18_037.lcd		
Method Filename	: MHET_BHET_rpamide_060721.lcm		
Batch Filename	: BHET_Colorimetric_37C_pH8_plate1_Commercials.lcb		
Vial #	: 3-30	Sample Type	: Unknown
Injection Volume	: 10 uL		
Date Acquired	: 8/25/2021 6:55:14 PM	Acquired by	: System Administrator
Date Processed	: 9/3/2021 9:03:21 AM	Processed by	: System Administrator

<Chromatogram>

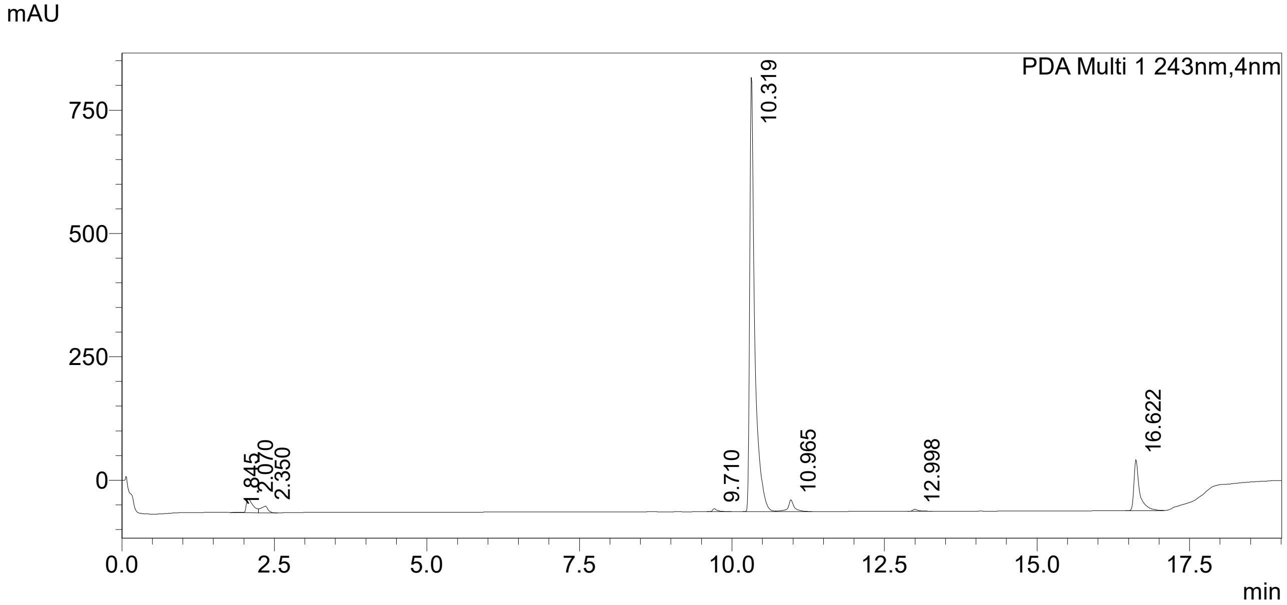

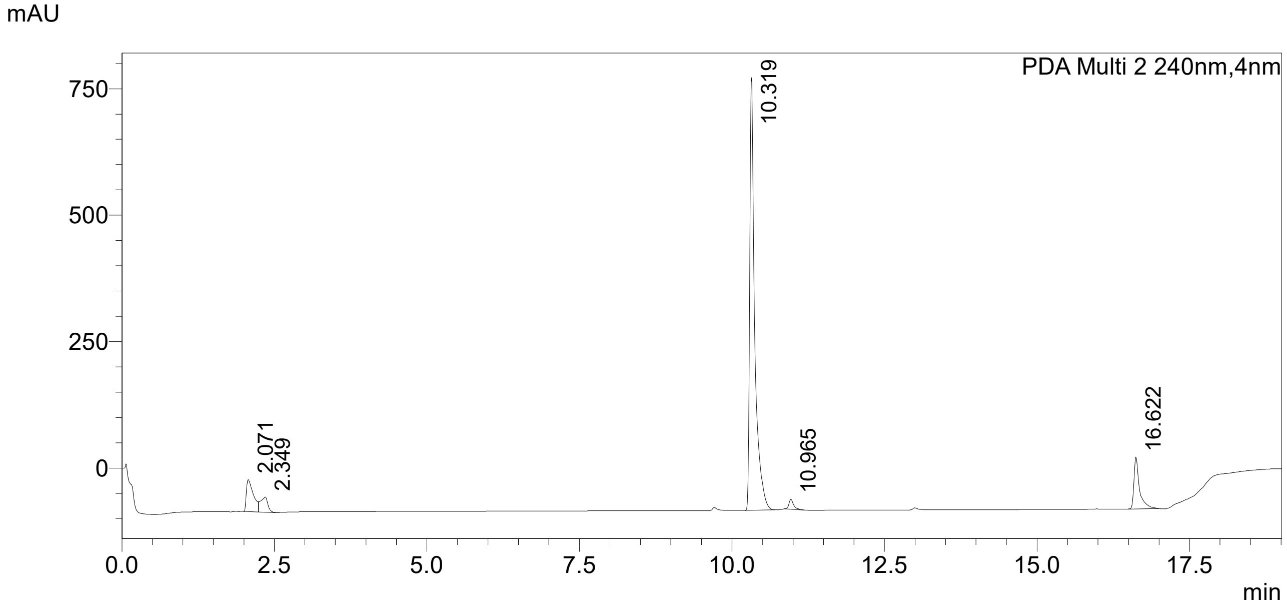

<Peak Table>

PDA Ch1 243nm

Peak#	Ret. Time	Area	Height	Conc.	Unit	Mark	Name
1	1.845	7700	611	0.000			
2	2.070	222776	28026	0.000		V	
3	2.350	107111	13322	0.000		V	
4	9.710	35887	6200	0.220	uM		MHET
5	10.319	5128896	880225	500.112	uM		BHET
6	10.965	172186	23824	0.000		V	
7	12.998	28001	4121	0.000			
8	16.622	668203	103152	0.000			
Total		6370760	1059481				

PDA Ch2 240nm

Peak#	Ret. Time	Area	Height	Conc.	Unit	Mark	Name
1	2.071	521014	62955	0.000			
2	2.349	243715	30100	0.000		V	
3	10.319	4963165	856162	0.000			
4	10.965	106675	19686	0.000			
5	16.622	651020	102152	0.000			
Total		6485589	1071056				
