# Supplementary material for: A flexible kinetic assay efficiently sorts prospective biocatalysts for PET plastic subunit hydrolysis
Source: RSC Adv. 2022 Mar 14;12(13):8119–30. doi: 10.1039/d2ra00612j (PMC8982334; doi:10.1039/d2ra00612j)
Supplement: RA-012-D2RA00612J-s021 [file RA-012-D2RA00612J-s021.pdf]

SHIMADZU LabSolutions

Analysis Report

<Sample Information>

Sample Name	: E2		
Sample ID	:		
Data Filename	: E2_009.lcd		
Method Filename	: MHET_BHET_rpamide_060721.lcm		
Batch Filename	: BHET_Colorimetric_37C_pH8_plate1_RECALC.lcb		
Vial #	: 4-22	Sample Type	: Unknown
Injection Volume	: 10 uL		
Date Acquired	: 8/24/2021 10:37:44 AM	Acquired by	: System Administrator
Date Processed	: 9/3/2021 9:16:14 AM	Processed by	: System Administrator

<Chromatogram>

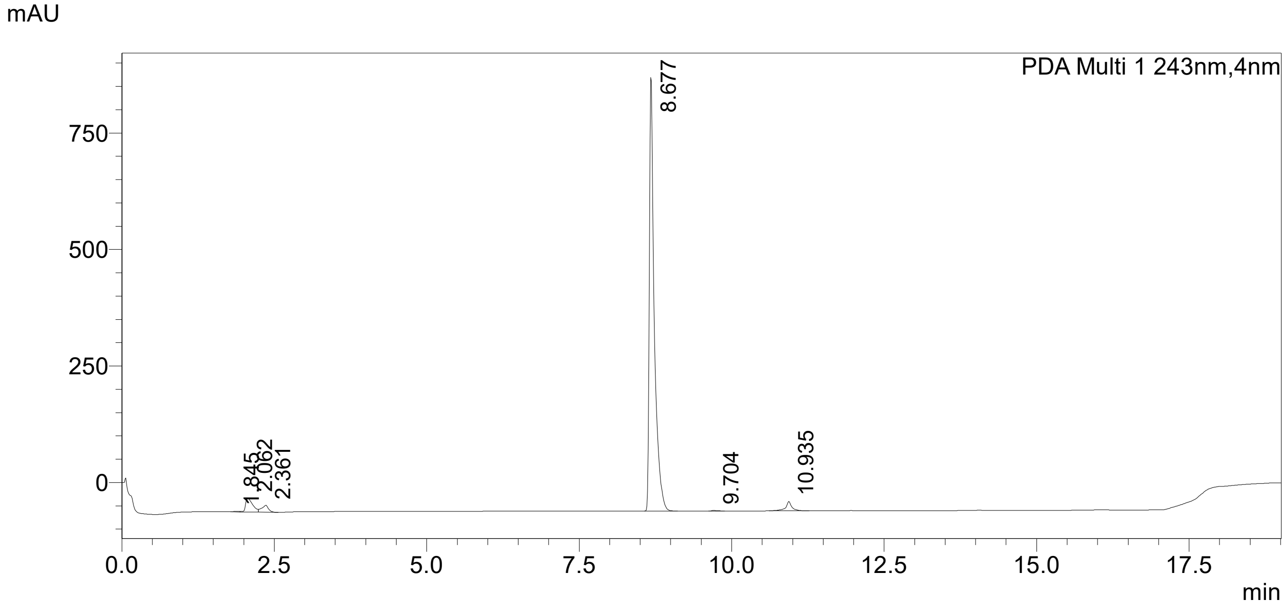

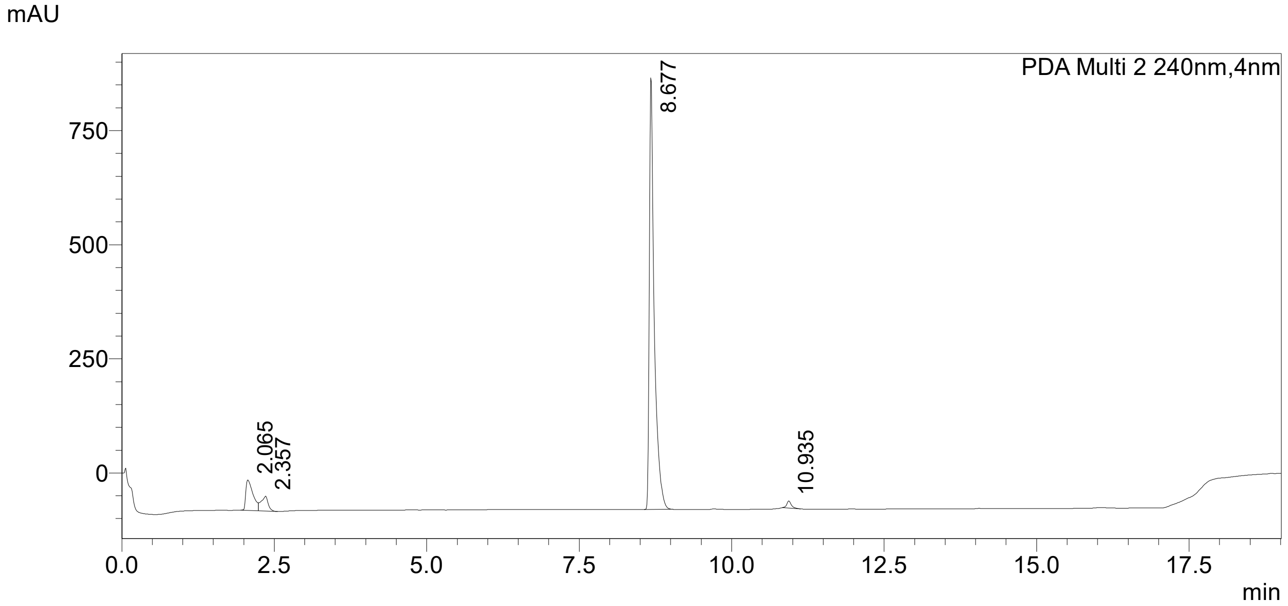

<Peak Table>

PDA Ch1 243nm

Peak#	Ret. Time	Area	Height	Conc.	Unit	Mark	Name
1	1.845	5732	691	0.000			
2	2.062	246468	30008	0.000		V	
3	2.361	115166	14915	0.000		V	
4	8.677	5324395	930305	0.000			
5	9.704	7256	1390	-2.414	uM		MHET
6	10.935	146489	19944	0.000			
Total		5845505	997253				

PDA Ch2 240nm

Peak#	Ret. Time	Area	Height	Conc.	Unit	Mark	Name
1	2.065	560383	66318	0.000			
2	2.357	256714	32598	0.000		V	
3	8.677	5389424	945277	524.870	uM		TPA
4	10.935	83874	15983	0.000			
Total		6290395	1060175				
