# Supplementary material for: A flexible kinetic assay efficiently sorts prospective biocatalysts for PET plastic subunit hydrolysis
Source: RSC Adv. 2022 Mar 14;12(13):8119–30. doi: 10.1039/d2ra00612j (PMC8982334; doi:10.1039/d2ra00612j)
Supplement: RA-012-D2RA00612J-s022 [file RA-012-D2RA00612J-s022.pdf]

SHIMADZU LabSolutions

Analysis Report

<Sample Information>

Sample Name	: E3		
Sample ID	:		
Data Filename	:E3 _012.lcd		
Method Filename	: MHET_BHET_rpamide_060721.lcm		
Batch Filename	: BHET_Colorimetric_37C_pH8_plate1_RECALC.lcb		
Vial #	: 4-25	Sample Type	: Unknown
Injection Volume	: 10 uL		
Date Acquired	: 8/24/2021 11:35:50 AM	Acquired by	: System Administrator
Date Processed	: 9/3/2021 9:16:09 AM	Processed by	: System Administrator

<Chromatogram>

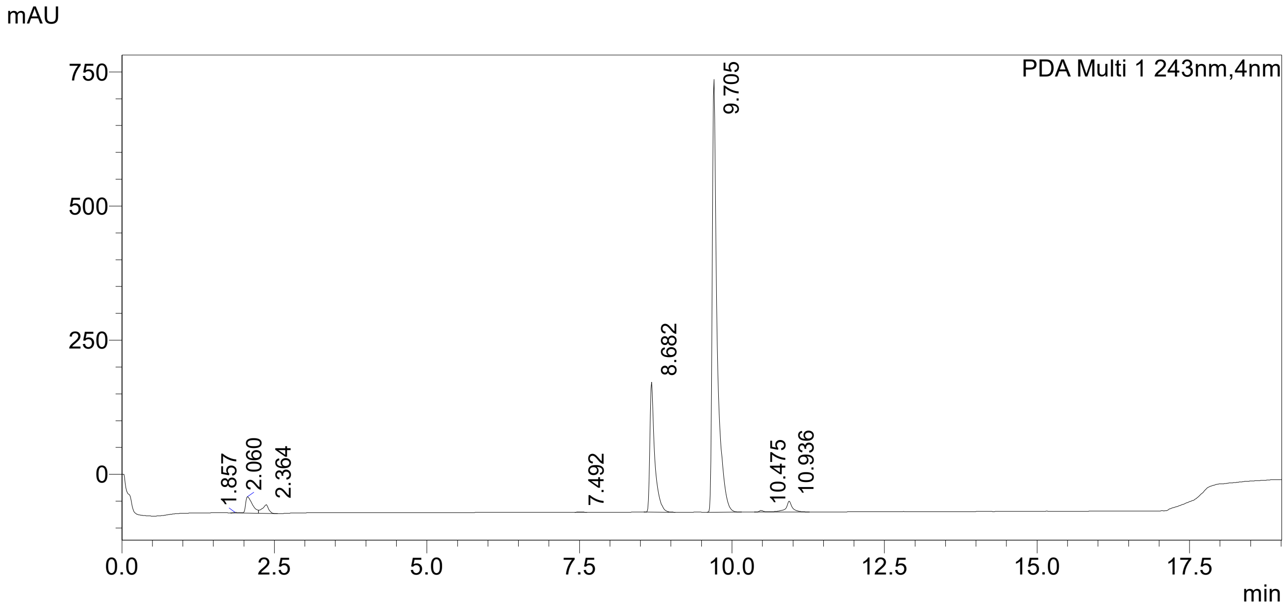

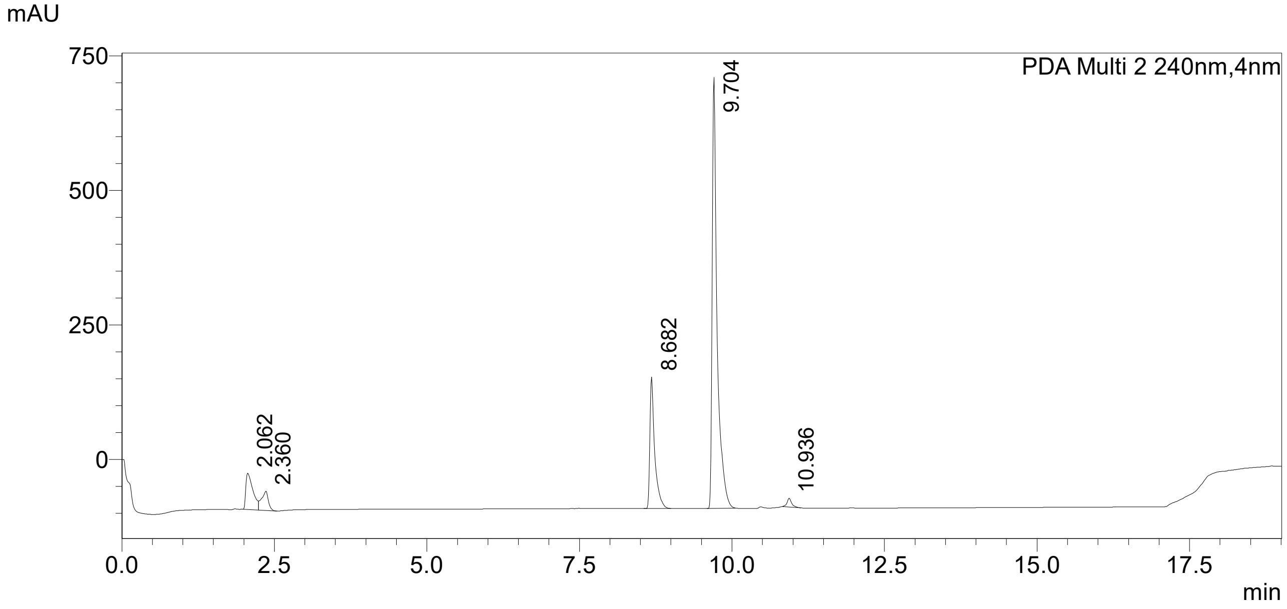

<Peak Table>

PDA Ch1 243nm

Peak#	Ret. Time	Area	Height	Conc.	Unit	Mark	Name
1	1.857	7278	1232	0.000			
2	2.060	247648	30664	0.000		V	
3	2.364	122311	16387	0.000		V	
4	7.492	2976	669	0.000			
5	8.682	1349455	242321	0.000			
6	9.705	4605635	806494	434.672	uM		MHET
7	10.475	16063	2840	-1.163	uM		BHET
8	10.936	155958	20548	0.000		V	
Total		6507325	1121155				

PDA Ch2 240nm

Peak#	Ret. Time	Area	Height	Conc.	Unit	Mark	Name
1	2.062	561641	67371	0.000			
2	2.360	273512	35820	0.000		V	
3	8.682	1358359	244529	124.167	uM		TPA
4	9.704	4561369	800775	0.000			
5	10.936	86210	16402	0.000			
Total		6841090	1164898				
