# Supplementary material for: A flexible kinetic assay efficiently sorts prospective biocatalysts for PET plastic subunit hydrolysis
Source: RSC Adv. 2022 Mar 14;12(13):8119–30. doi: 10.1039/d2ra00612j (PMC8982334; doi:10.1039/d2ra00612j)
Supplement: RA-012-D2RA00612J-s023 [file RA-012-D2RA00612J-s023.pdf]

SHIMADZU LabSolutions

Analysis Report

<Sample Information>

Sample Name	:E4		
Sample ID	:		
Data Filename	: E4 _024.lcd		
Method Filename	: MHET_BHET_rpamide_060721.lcm		
Batch Filename	: BHET_Colorimetric_37C_pH8_plate2.lcb		
Vial #	: 4-16	Sample Type	: Unknown
Injection Volume	: 10 uL		
Date Acquired	: 8/26/2021 3:34:37 PM	Acquired by	: System Administrator
Date Processed	: 9/3/2021 8:54:39 AM	Processed by	: System Administrator

<Chromatogram>

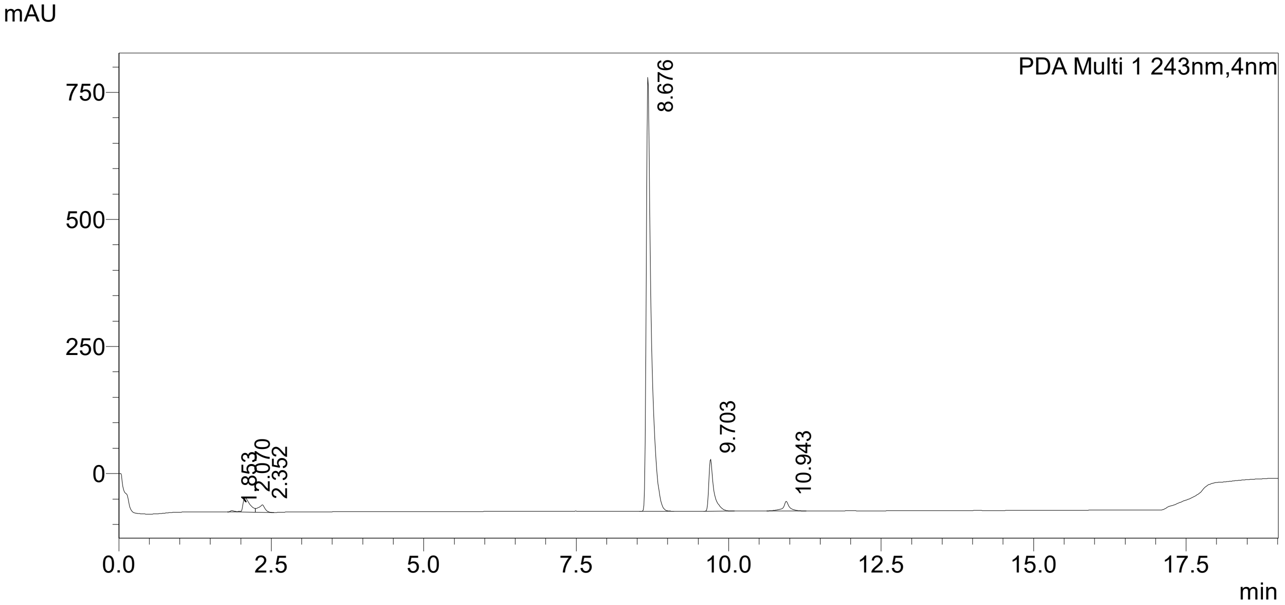

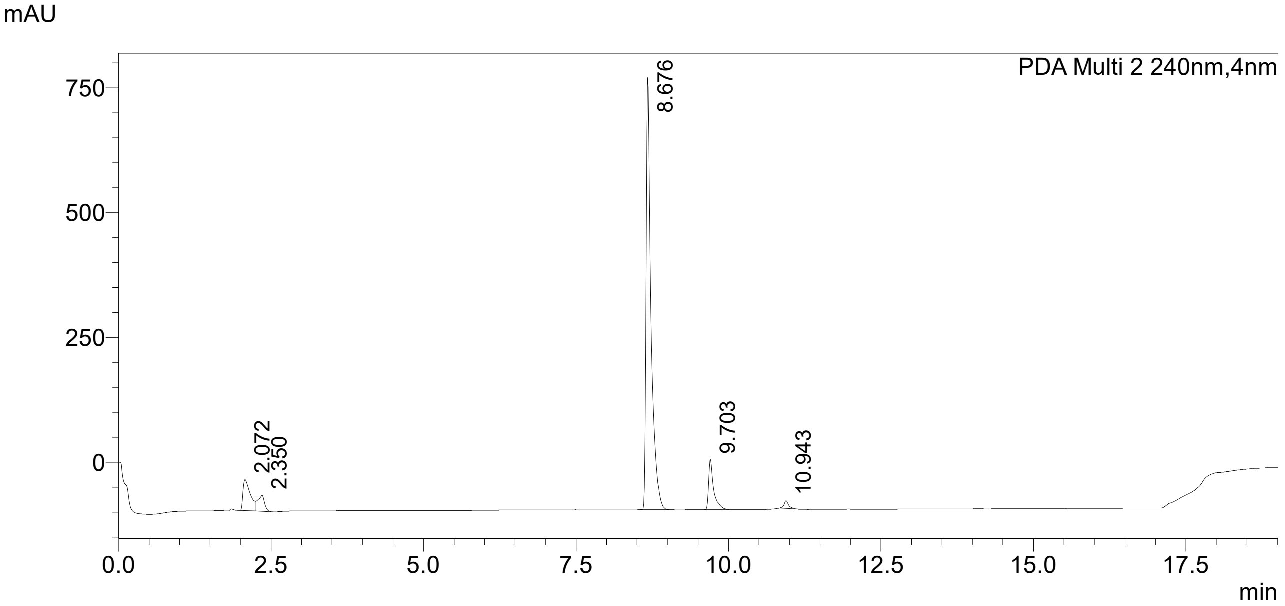

<Peak Table>

PDA Ch1 243nm

Peak#	Ret. Time	Area	Height	Conc.	Unit	Mark	Name
1	1.853	14812	2624	0.000			
2	2.070	234530	27952	0.000		V	
3	2.352	112530	14503	0.000		V	
4	8.676	4914744	853429	0.000			
5	9.703	590330	101470	51.239	uM		MHET
6	10.943	140028	19138	0.000			
Total		6006974	1019116				

PDA Ch2 240nm

Peak#	Ret. Time	Area	Height	Conc.	Unit	Mark	Name
1	2.072	528513	62125	0.000			
2	2.350	252942	32252	0.000		V	
3	8.676	4969003	865747	479.793	uM		TPA
4	9.703	578617	100246	0.000			
5	10.943	79934	15291	0.000			
Total		6409010	1075660				
