# Supplementary material for: A flexible kinetic assay efficiently sorts prospective biocatalysts for PET plastic subunit hydrolysis
Source: RSC Adv. 2022 Mar 14;12(13):8119–30. doi: 10.1039/d2ra00612j (PMC8982334; doi:10.1039/d2ra00612j)
Supplement: RA-012-D2RA00612J-s024 [file RA-012-D2RA00612J-s024.pdf]

SHIMADZU LabSolutions

Analysis Report

<Sample Information>

Sample Name	: E5		
Sample ID	:		
Data Filename	: E5 _024.lcd		
Method Filename	: MHET_BHET_rpamide_060721.lcm		
Batch Filename	: BHET_Colorimetric_37C_pH8_plate1_RECALC.lcb		
Vial #	: 4-37	Sample Type	: Unknown
Injection Volume	: 10 uL		
Date Acquired	: 8/24/2021 2:32:58 PM	Acquired by	: System Administrator
Date Processed	: 9/3/2021 9:15:54 AM	Processed by	: System Administrator

<Chromatogram>

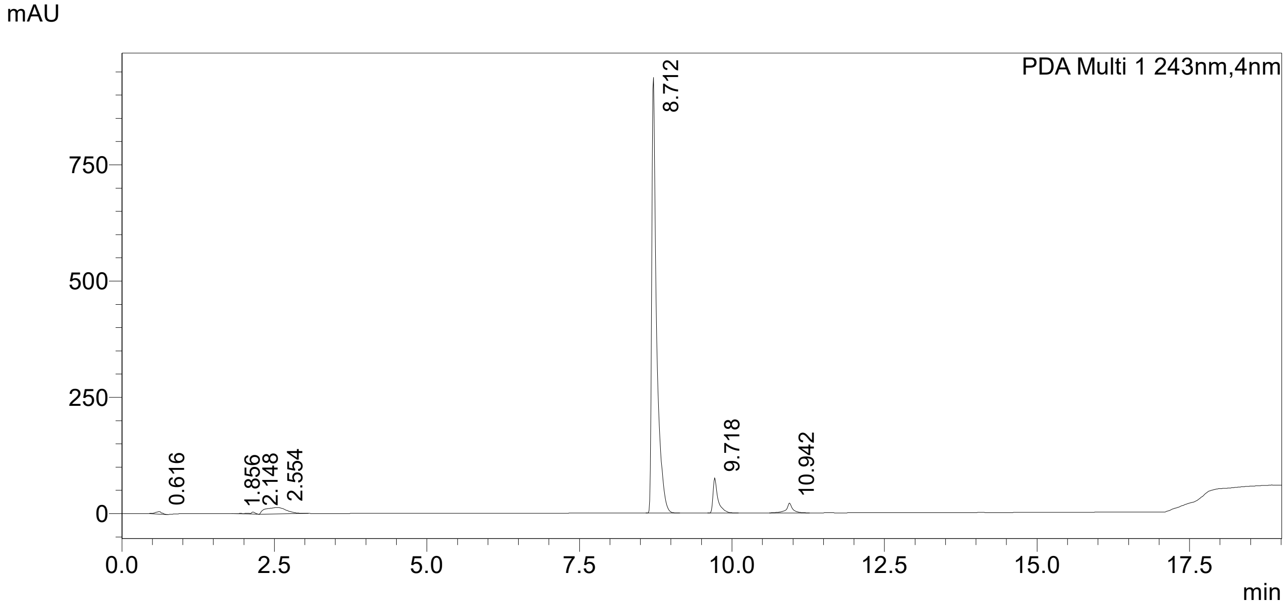

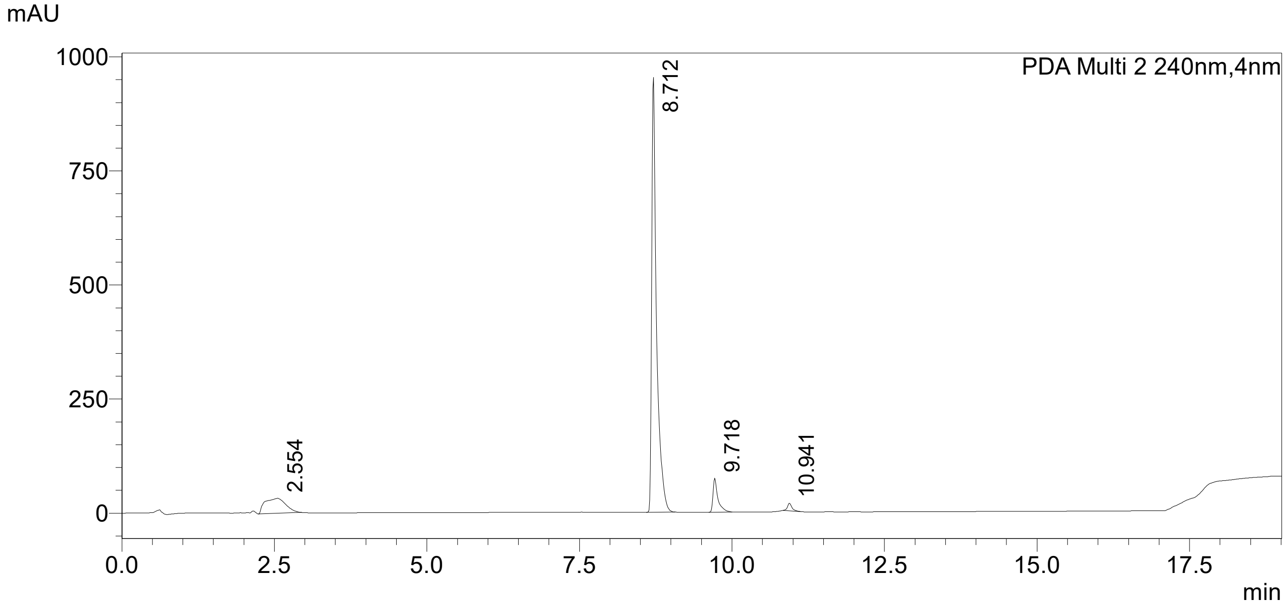

<Peak Table>

PDA Ch1 243nm

Peak#	Ret. Time	Area	Height	Conc.	Unit	Mark	Name
1	0.616	34934	5337	0.000			
2	1.856	17134	702	0.000			
3	2.148	20185	4346	0.000		V	
4	2.554	342236	14047	0.000			
5	8.712	5245291	936619	0.000			
6	9.718	423667	75137	35.903	uM		MHET
7	10.942	152766	20898	0.000			
Total		6236212	1057086				

PDA Ch2 240nm

Peak#	Ret. Time	Area	Height	Conc.	Unit	Mark	Name
1	2.554	788772	32851	0.000			
2	8.712	5314957	952696	516.082	uM		TPA
3	9.718	412231	74202	0.000			
4	10.941	86096	16627	0.000			
Total		6602055	1076376				
