# Supplementary material for: A flexible kinetic assay efficiently sorts prospective biocatalysts for PET plastic subunit hydrolysis
Source: RSC Adv. 2022 Mar 14;12(13):8119–30. doi: 10.1039/d2ra00612j (PMC8982334; doi:10.1039/d2ra00612j)
Supplement: RA-012-D2RA00612J-s025 [file RA-012-D2RA00612J-s025.pdf]

SHIMADZU LabSolutions

Analysis Report

<Sample Information>

Sample Name	: E6		
Sample ID	:		
Data Filename	: E6 _027.lcd		
Method Filename	: MHET_BHET_rpamide_060721.lcm		
Batch Filename	: BHET_Colorimetric_37C_pH8_plate1.lcb		
Vial #	: 4-40	Sample Type	: Unknown
Injection Volume	: 10 uL		
Date Acquired	: 8/24/2021 3:31:01 PM	Acquired by	: System Administrator
Date Processed	: 9/3/2021 9:06:21 AM	Processed by	: System Administrator

<Chromatogram>

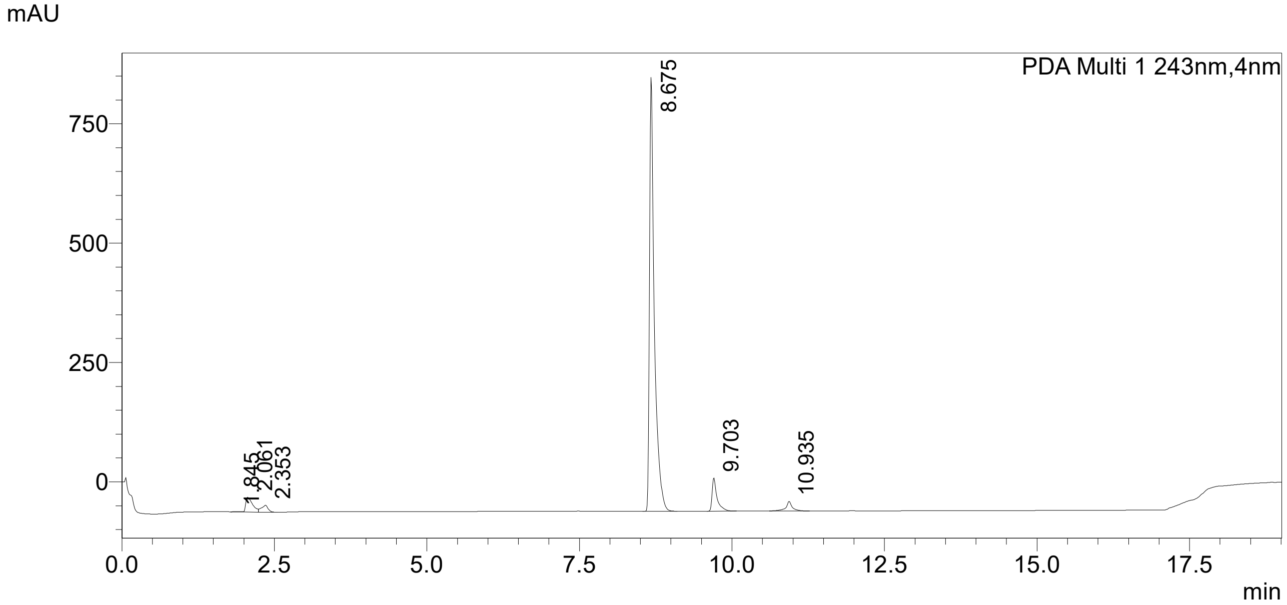

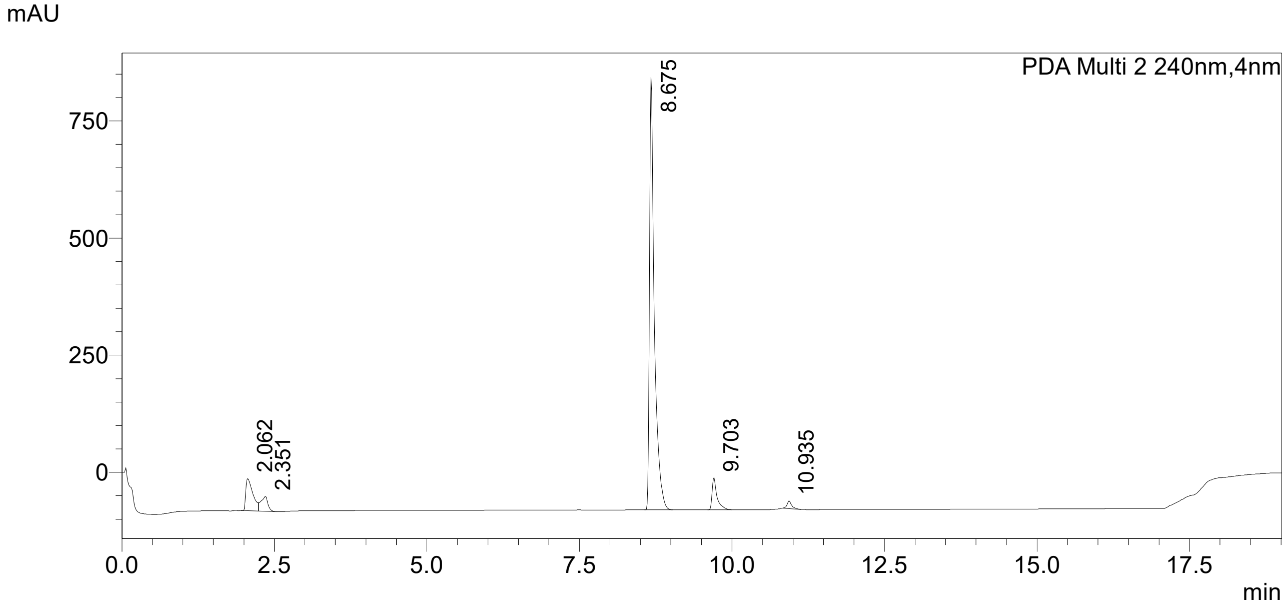

<Peak Table>

PDA Ch1 243nm

Peak#	Ret. Time	Area	Height	Conc.	Unit	Mark	Name
1	1.845	8404	698	0.000			
2	2.061	245585	30889	0.000		V	
3	2.353	111238	14704	0.000		V	
4	8.675	5134327	908478	0.000			
5	9.703	395170	69230	33.281	uM		MHET
6	10.935	147709	20191	0.000			
Total		6042433	1044190				

PDA Ch2 240nm

Peak#	Ret. Time	Area	Height	Conc.	Unit	Mark	Name
1	2.062	570643	68544	0.000			
2	2.351	250098	32288	0.000		V	
3	8.675	5195439	922665	501.977	uM		TPA
4	9.703	385667	68375	0.000			
5	10.935	84728	16196	0.000			
Total		6486574	1108068				
