# Supplementary material for: A flexible kinetic assay efficiently sorts prospective biocatalysts for PET plastic subunit hydrolysis
Source: RSC Adv. 2022 Mar 14;12(13):8119–30. doi: 10.1039/d2ra00612j (PMC8982334; doi:10.1039/d2ra00612j)
Supplement: RA-012-D2RA00612J-s026 [file RA-012-D2RA00612J-s026.pdf]

SHIMADZU LabSolutions

Analysis Report

<Sample Information>

Sample Name	: E7		
Sample ID	:		
Data Filename	: E7 _006.lcd		
Method Filename	: MHET_BHET_rpamide_060721.lcm		
Batch Filename	: BHET_Colorimetric_37C_pH8_plate1.lcb		
Vial #	: 4-48	Sample Type	: Unknown
Injection Volume	: 10 uL		
Date Acquired	: 8/24/2021 6:05:58 PM	Acquired by	: System Administrator
Date Processed	: 9/3/2021 9:06:35 AM	Processed by	: System Administrator

<Chromatogram>

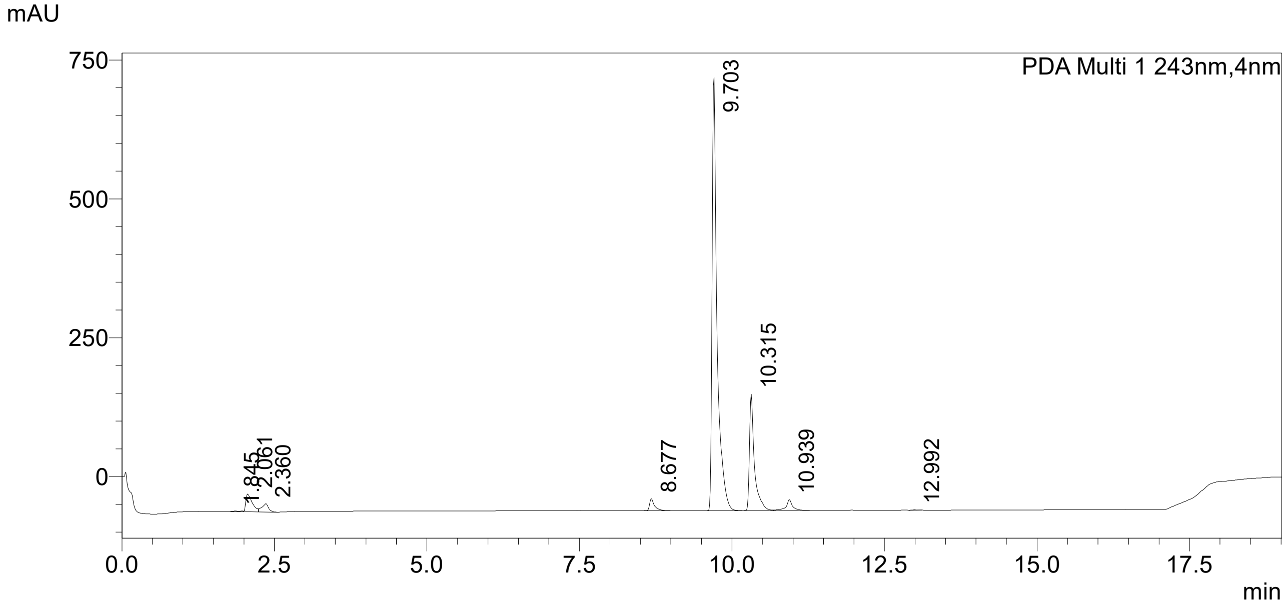

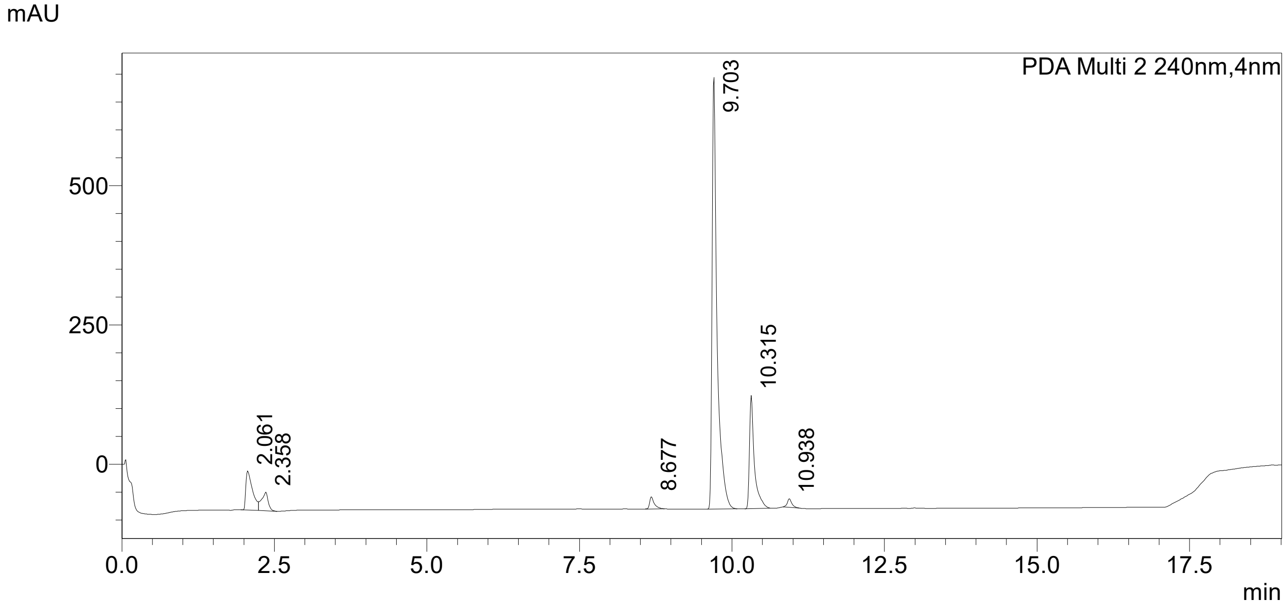

<Peak Table>

PDA Ch1 243nm

Peak#	Ret. Time	Area	Height	Conc.	Unit	Mark	Name
1	1.845	8290	686	0.000			
2	2.061	236108	31585	0.000		V	
3	2.360	110873	15066	0.000		V	
4	8.677	120180	21360	0.000			
5	9.703	4467328	779790	421.860	uM		MHET
6	10.315	1201463	209699	112.287	uM		BHET
7	10.939	148427	19492	0.000		V	
8	12.992	3249	649	0.000			
Total		6295918	1078327				

PDA Ch2 240nm

Peak#	Ret. Time	Area	Height	Conc.	Unit	Mark	Name
1	2.061	547036	69786	0.000			
2	2.358	249939	33265	0.000		V	
3	8.677	116230	21384	7.982	uM		TPA
4	9.703	4424033	774038	0.000			
5	10.315	1150471	203219	0.000			
6	10.938	80593	15328	0.000			
Total		6568302	1117021				
