# Supplementary material for: A flexible kinetic assay efficiently sorts prospective biocatalysts for PET plastic subunit hydrolysis
Source: RSC Adv. 2022 Mar 14;12(13):8119–30. doi: 10.1039/d2ra00612j (PMC8982334; doi:10.1039/d2ra00612j)
Supplement: RA-012-D2RA00612J-s027 [file RA-012-D2RA00612J-s027.pdf]

SHIMADZU LabSolutions

Analysis Report

<Sample Information>

Sample Name	: E8		
Sample ID	:		
Data Filename	: E8 _009.lcd		
Method Filename	: MHET_BHET_rpamide_060721.lcm		
Batch Filename	: BHET_Colorimetric_37C_pH8_plate1.lcb		
Vial #	: 4-51	Sample Type	: Unknown
Injection Volume	: 10 uL		
Date Acquired	: 8/24/2021 7:04:03 PM	Acquired by	: System Administrator
Date Processed	: 9/3/2021 9:06:41 AM	Processed by	: System Administrator

<Chromatogram>

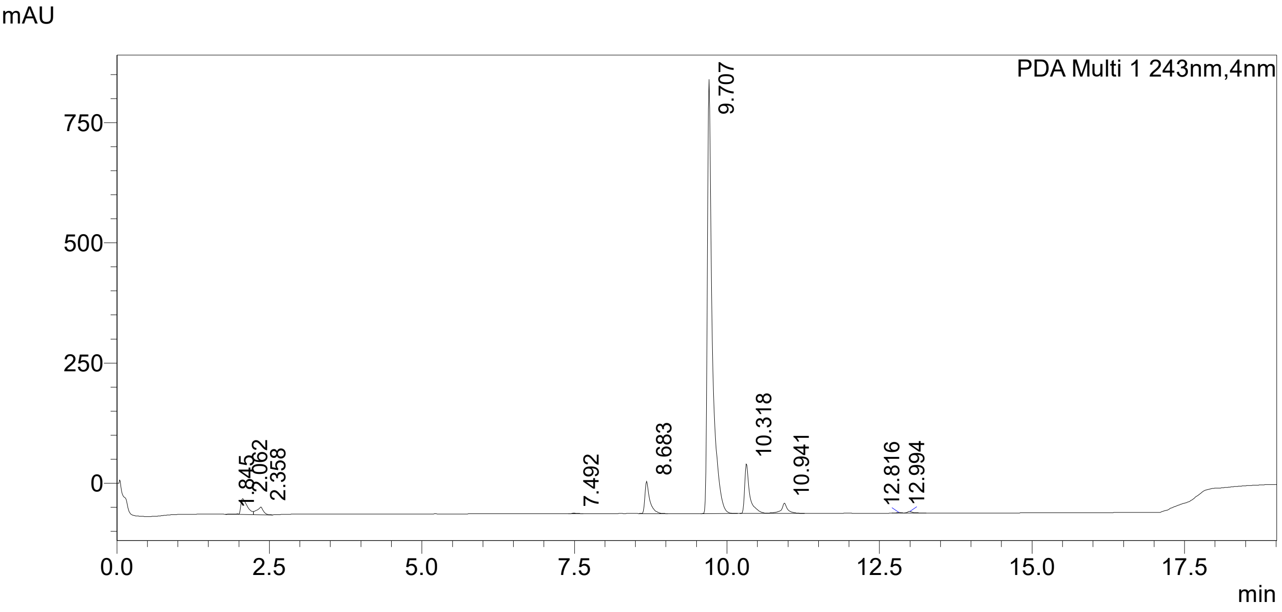

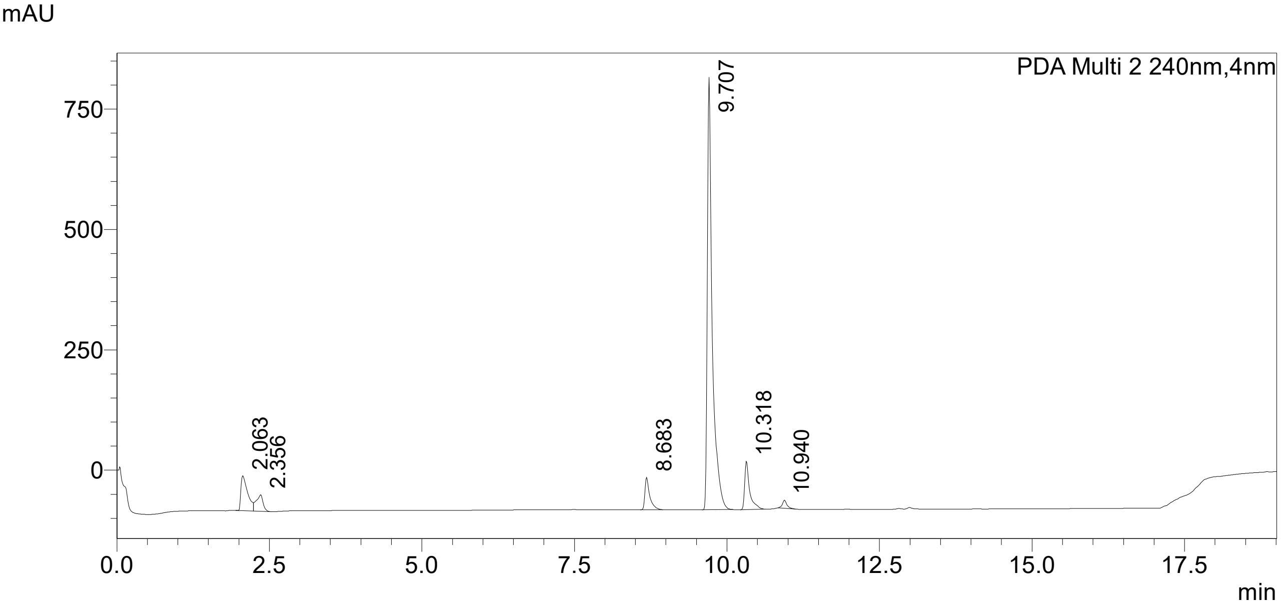

<Peak Table>

PDA Ch1 243nm

Peak#	Ret. Time	Area	Height	Conc.	Unit	Mark	Name
1	1.845	9392	826	0.000			
2	2.062	248453	32755	0.000		V	
3	2.358	117583	15741	0.000		V	
4	7.492	4533	964	0.000			
5	8.683	370485	66867	0.000			
6	9.707	5131915	903134	483.422	uM		MHET
7	10.318	600295	102984	54.752	uM		BHET
8	10.941	158757	21086	0.000		V	
9	12.816	8357	1416	0.000			
10	12.994	23268	3468	0.000		V	
Total		6673038	1149241				

PDA Ch2 240nm

Peak#	Ret. Time	Area	Height	Conc.	Unit	Mark	Name
1	2.063	575354	72405	0.000			
2	2.356	265290	34701	0.000		V	
3	8.683	369172	67336	31.641	uM		TPA
4	9.707	5086891	897801	0.000			
5	10.318	569472	99684	0.000			
6	10.940	86524	16632	0.000			
Total		6952703	1188558				
