# Supplementary material for: A flexible kinetic assay efficiently sorts prospective biocatalysts for PET plastic subunit hydrolysis
Source: RSC Adv. 2022 Mar 14;12(13):8119–30. doi: 10.1039/d2ra00612j (PMC8982334; doi:10.1039/d2ra00612j)
Supplement: RA-012-D2RA00612J-s028 [file RA-012-D2RA00612J-s028.pdf]

SHIMADZU LabSolutions

Analysis Report

<Sample Information>

Sample Name	:E9		
Sample ID	:		
Data Filename	: E9_009.lcd		
Method Filename	: MHET_BHET_rpamide_060721.lcm		
Batch Filename	: BHET_Colorimetric_37C_pH8_plate1.lcb		
Vial #	: 3-3	Sample Type	: Unknown
Injection Volume	: 10 uL		
Date Acquired	: 8/24/2021 9:37:21 PM	Acquired by	: System Administrator
Date Processed	: 9/3/2021 9:06:52 AM	Processed by	: System Administrator

<Chromatogram>

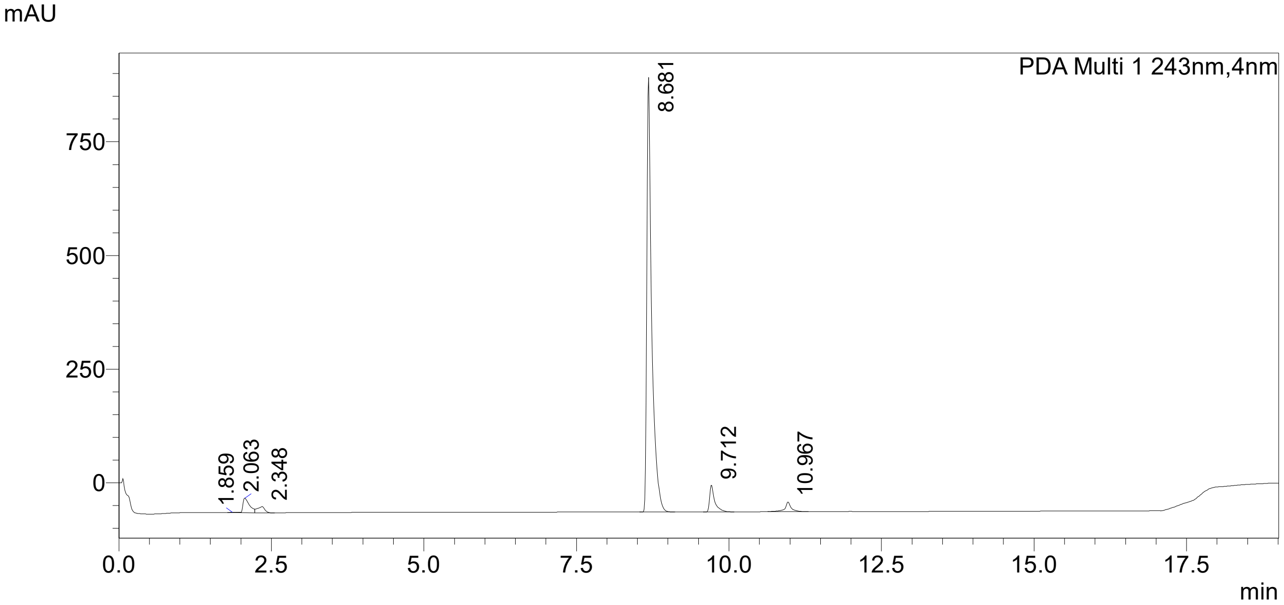

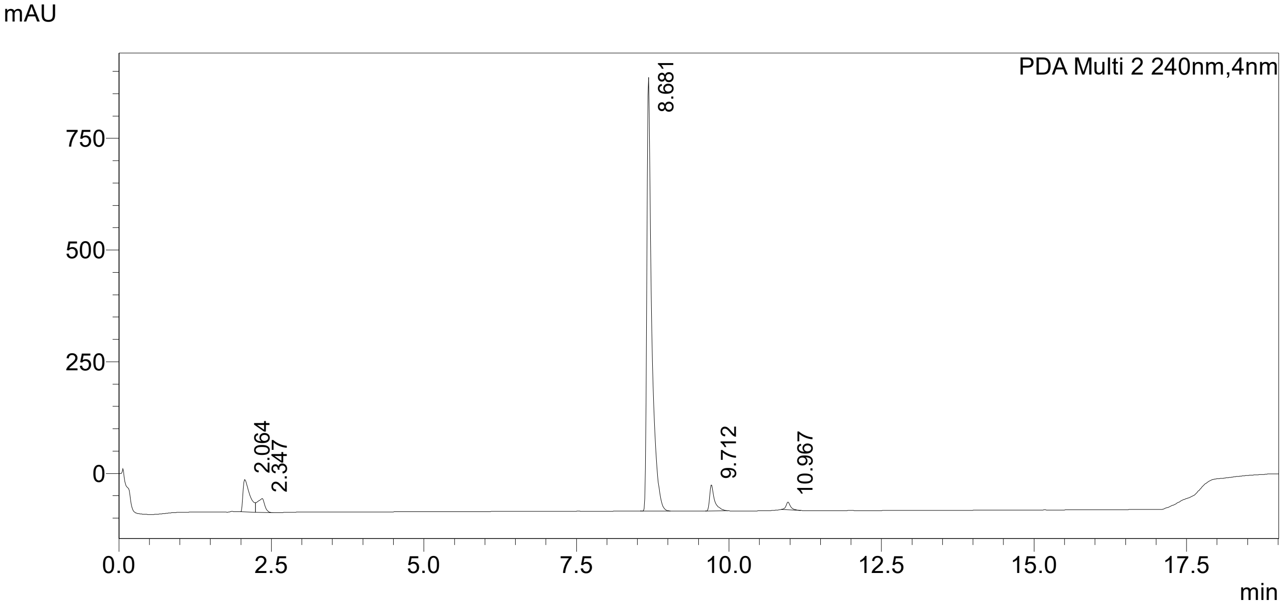

<Peak Table>

PDA Ch1 243nm

Peak#	Ret. Time	Area	Height	Conc.	Unit	Mark	Name
1	1.859	6515	1130	0.000			
2	2.063	249117	32264	0.000		V	
3	2.348	117235	13781	0.000		V	
4	8.681	5415011	955628	0.000			
5	9.712	339927	58624	28.197	uM		MHET
6	10.967	152946	21542	0.000			
Total		6280751	1082968				

PDA Ch2 240nm

Peak#	Ret. Time	Area	Height	Conc.	Unit	Mark	Name
1	2.064	585386	71889	0.000			
2	2.347	254146	31111	0.000		V	
3	8.681	5480464	970557	535.613	uM		TPA
4	9.712	330027	57840	0.000			
5	10.967	90199	17465	0.000			
Total		6740222	1148863				
