# Supplementary material for: A flexible kinetic assay efficiently sorts prospective biocatalysts for PET plastic subunit hydrolysis
Source: RSC Adv. 2022 Mar 14;12(13):8119–30. doi: 10.1039/d2ra00612j (PMC8982334; doi:10.1039/d2ra00612j)
Supplement: RA-012-D2RA00612J-s029 [file RA-012-D2RA00612J-s029.pdf]

SHIMADZU LabSolutions

Analysis Report

<Sample Information>

Sample Name	: t=0 control Plate 1		
Sample ID	:		
Data Filename	: t=0 control Plate 1_040.lcd		
Method Filename	: MHET_BHET_rpamide_060721.lcm		
Batch Filename	: BHET_Colorimetric_37C_pH8_plate1_Commercials.lcb		
Vial #	: 3-33	Sample Type	: Unknown
Injection Volume	: 10 uL		
Date Acquired	: 8/25/2021 7:53:14 PM	Acquired by	: System Administrator
Date Processed	: 9/3/2021 9:03:26 AM	Processed by	: System Administrator

<Chromatogram>

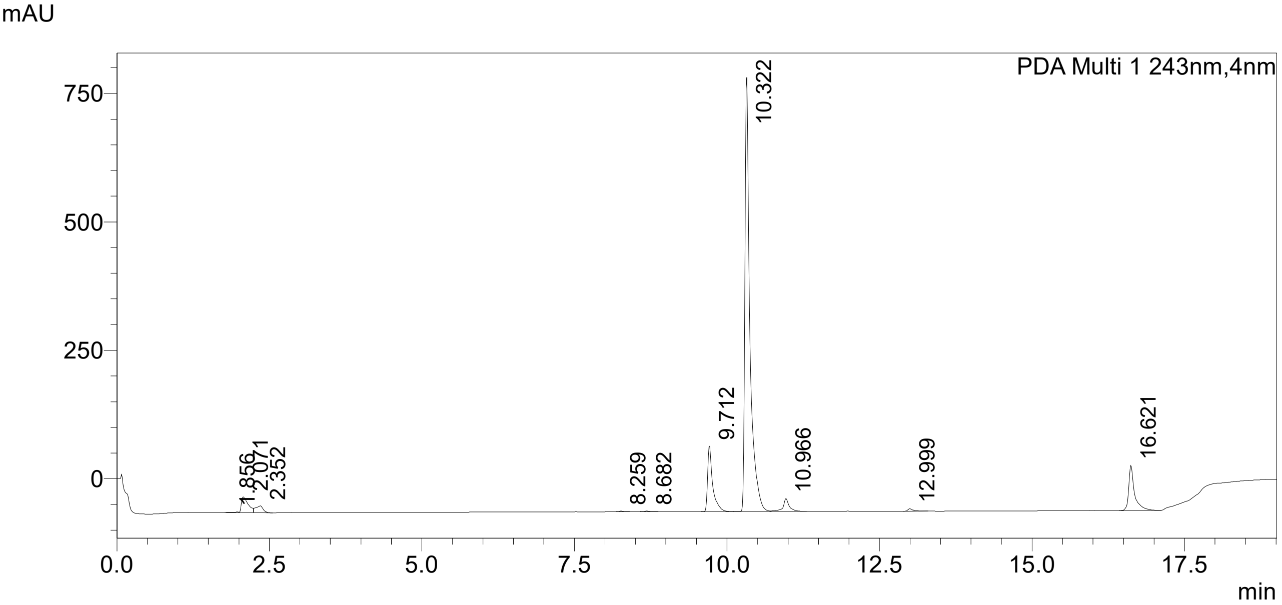

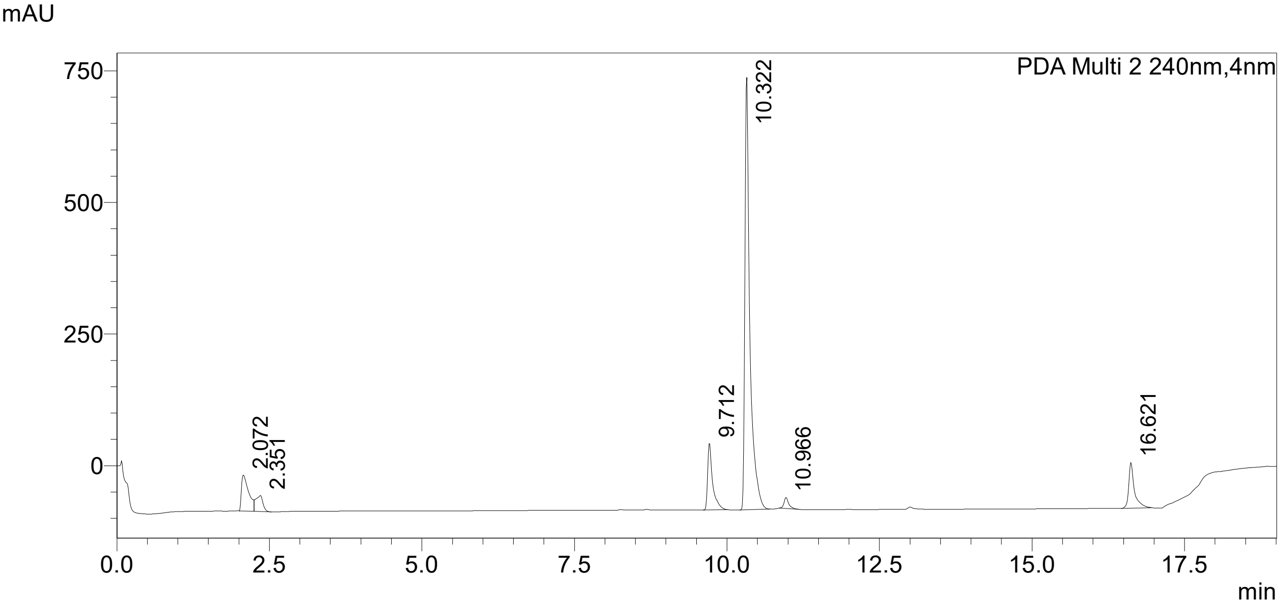

<Peak Table>

PDA Ch1 243nm

Peak#	Ret. Time	Area	Height	Conc.	Unit	Mark	Name
1	1.856	8296	740	0.000			
2	2.071	243165	30541	0.000		V	
3	2.352	113130	13449	0.000		V	
4	8.259	5605	1238	0.000			
5	8.682	7148	1312	0.000			
6	9.712	741431	127567	65.143	uM		MHET
7	10.322	4896151	844957	477.941	uM	V	BHET
8	10.966	184399	25410	0.000		V	
9	12.999	30876	4567	0.000			
10	16.621	588370	87655	0.000			
Total		6818571	1137436				

PDA Ch2 240nm

Peak#	Ret. Time	Area	Height	Conc.	Unit	Mark	Name
1	2.072	580697	68443	0.000			
2	2.351	242978	30367	0.000		V	
3	9.712	727606	126052	0.000			
4	10.322	4737225	821416	0.000			
5	10.966	113676	20977	0.000			
6	16.621	567709	86712	0.000			
Total		6969890	1153968				
