# Supplementary material for: A flexible kinetic assay efficiently sorts prospective biocatalysts for PET plastic subunit hydrolysis
Source: RSC Adv. 2022 Mar 14;12(13):8119–30. doi: 10.1039/d2ra00612j (PMC8982334; doi:10.1039/d2ra00612j)
Supplement: RA-012-D2RA00612J-s030 [file RA-012-D2RA00612J-s030.pdf]

SHIMADZU LabSolutions

Analysis Report

<Sample Information>

Sample Name	: t=24hr control Plate 1		
Sample ID	:		
Data Filename	: t=24hr control Plate 1_043.lcd		
Method Filename	: MHET_BHET_rpamide_060721.lcm		
Batch Filename	: BHET_Colorimetric_37C_pH8_plate1_Commercials.lcb		
Vial #	: 3-36	Sample Type	: Unknown
Injection Volume	: 10 uL		
Date Acquired	: 8/25/2021 8:51:16 PM	Acquired by	: System Administrator
Date Processed	: 9/3/2021 9:03:32 AM	Processed by	: System Administrator

<Chromatogram>

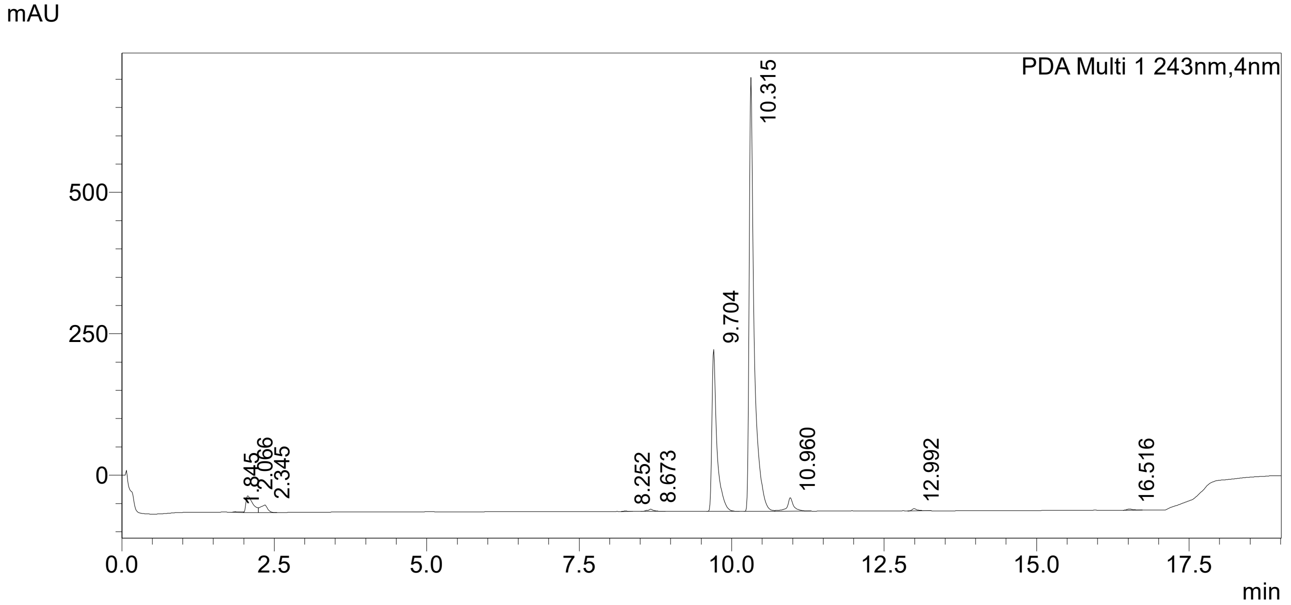

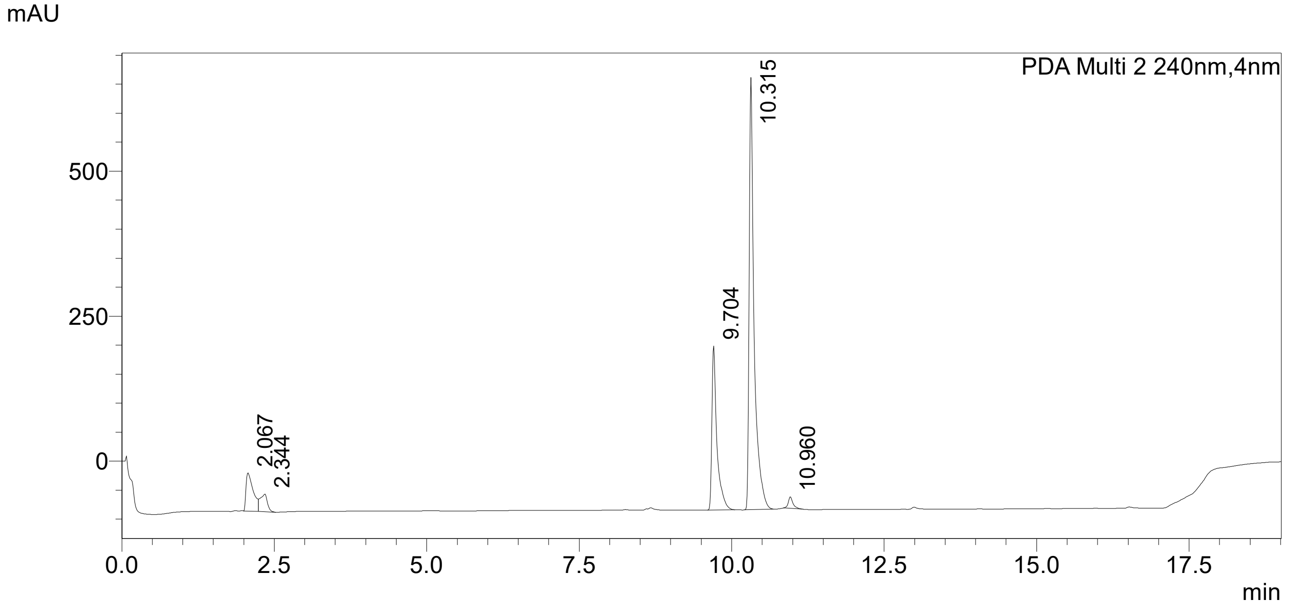

<Peak Table>

PDA Ch1 243nm

Peak#	Ret. Time	Area	Height	Conc.	Unit	Mark	Name
1	1.845	9899	994	0.000			
2	2.066	239593	29150	0.000		V	
3	2.345	107769	13479	0.000		V	
4	8.252	4114	968	0.000			
5	8.673	29213	3946	0.000			
6	9.704	1647514	286161	151.186	uM		MHET
7	10.315	4423166	767049	432.927	uM		BHET
8	10.960	175461	23838	0.000		V	
9	12.992	25111	3816	0.000			
10	16.516	13941	2169	0.000			
Total		6675782	1131570				

PDA Ch2 240nm

Peak#	Ret. Time	Area	Height	Conc.	Unit	Mark	Name
1	2.067	557233	65581	0.000			
2	2.344	243376	30331	0.000		V	
3	9.704	1625422	283083	0.000			
4	10.315	4275713	745283	0.000			
5	10.960	109237	19687	0.000			
Total		6810980	1143965				
