# Supplementary material for: A flexible kinetic assay efficiently sorts prospective biocatalysts for PET plastic subunit hydrolysis
Source: RSC Adv. 2022 Mar 14;12(13):8119–30. doi: 10.1039/d2ra00612j (PMC8982334; doi:10.1039/d2ra00612j)
Supplement: RA-012-D2RA00612J-s031 [file RA-012-D2RA00612J-s031.pdf]

SHIMADZU LabSolutions

Analysis Report

<Sample Information>

Sample Name	: 0 hr Control ER1 50C		
Sample ID	:		
Data Filename	: 0 hr Control ER1 50C _032.lcd		
Method Filename	: MHET_BHET_rpamide_060721.lcm		
Batch Filename	: BHET_Colorimetric_50C_pH8_plate1.lcb		
Vial #	: 4-22	Sample Type	: Unknown
Injection Volume	: 10 uL		
Date Acquired	: 8/30/2021 10:34:57 PM	Acquired by	: System Administrator
Date Processed	: 9/3/2021 8:52:23 AM	Processed by	: System Administrator

<Chromatogram>

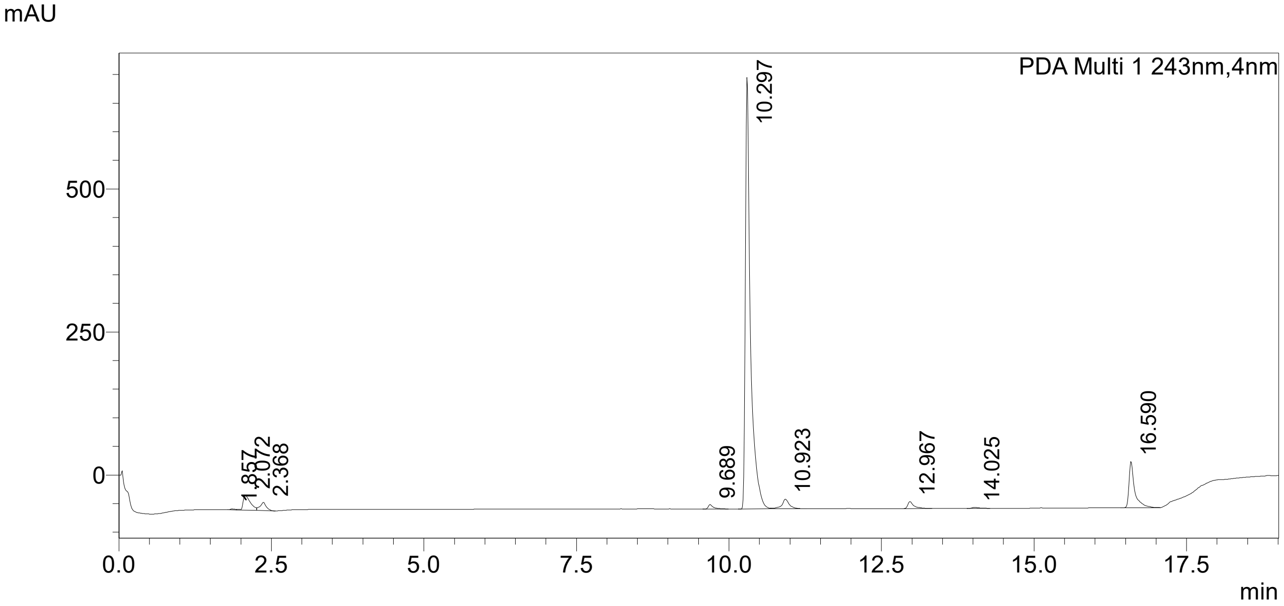

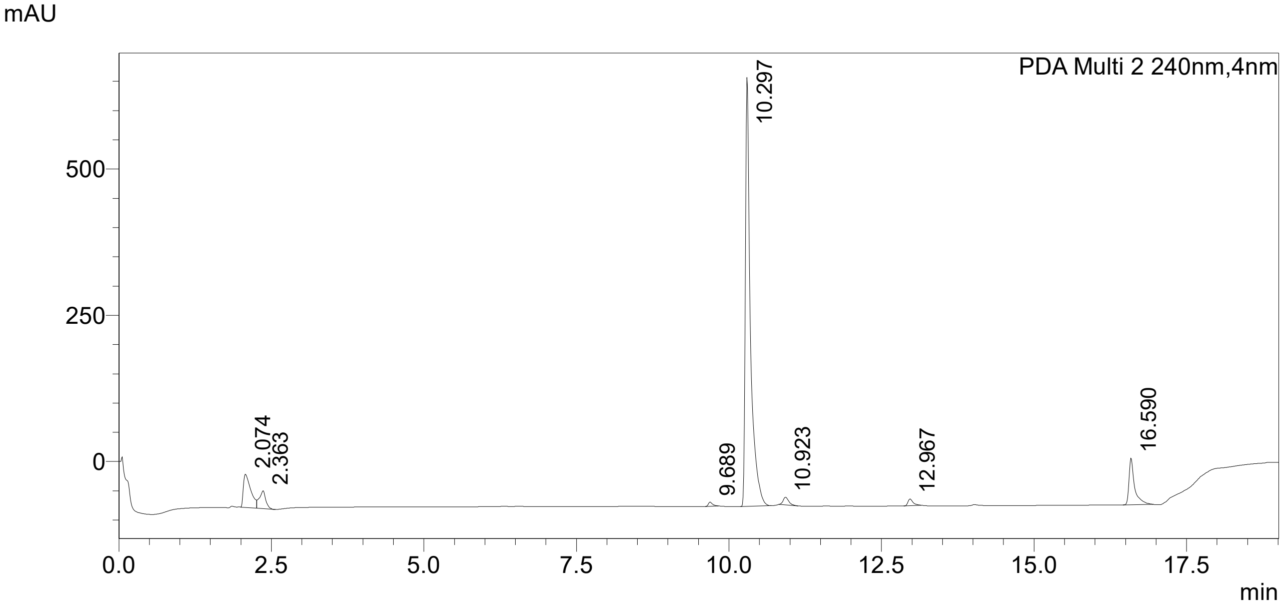

<Peak Table>

PDA Ch1 243nm

Peak#	Ret. Time	Area	Height	Conc.	Unit	Mark	Name
1	1.857	10188	1813	0.000			
2	2.072	223289	26295	0.000		V	
3	2.368	101759	14743	0.000		V	
4	9.689	45593	7699	1.113	uM		MHET
5	10.297	4446971	754231	435.193	uM		BHET
6	10.923	149715	16955	0.000		V	
7	12.967	85706	12261	0.000			
8	14.025	14394	1899	0.000			
9	16.590	529497	80360	0.000			
Total		5607112	916256				

PDA Ch2 240nm

Peak#	Ret. Time	Area	Height	Conc.	Unit	Mark	Name
1	2.074	492842	56665	0.000			
2	2.363	218944	30593	0.000		V	
3	9.689	35495	7219	0.000			
4	10.297	4303365	733176	0.000			
5	10.923	86313	13274	0.000			
6	12.967	68904	11561	0.000			
7	16.590	512411	79524	0.000			
Total		5718275	932013				
