# Supplementary material for: A flexible kinetic assay efficiently sorts prospective biocatalysts for PET plastic subunit hydrolysis
Source: RSC Adv. 2022 Mar 14;12(13):8119–30. doi: 10.1039/d2ra00612j (PMC8982334; doi:10.1039/d2ra00612j)
Supplement: RA-012-D2RA00612J-s032 [file RA-012-D2RA00612J-s032.pdf]

Analysis Report

<Sample Information>

Sample Name	: 24 hr Control ER1 50C		
Sample ID	:		
Data Filename	: 24 hr Control ER1 50C _029.lcd		
Method Filename	: MHET_BHET_rpamide_060721.lcm		
Batch Filename	: BHET_Colorimetric_50C_pH8_plate1.lcb		
Vial #	: 4-19	Sample Type	: Unknown
Injection Volume	: 10 uL		
Date Acquired	: 8/30/2021 9:36:53 PM	Acquired by	: System Administrator
Date Processed	: 9/3/2021 8:52:17 AM	Processed by	: System Administrator

<Chromatogram>

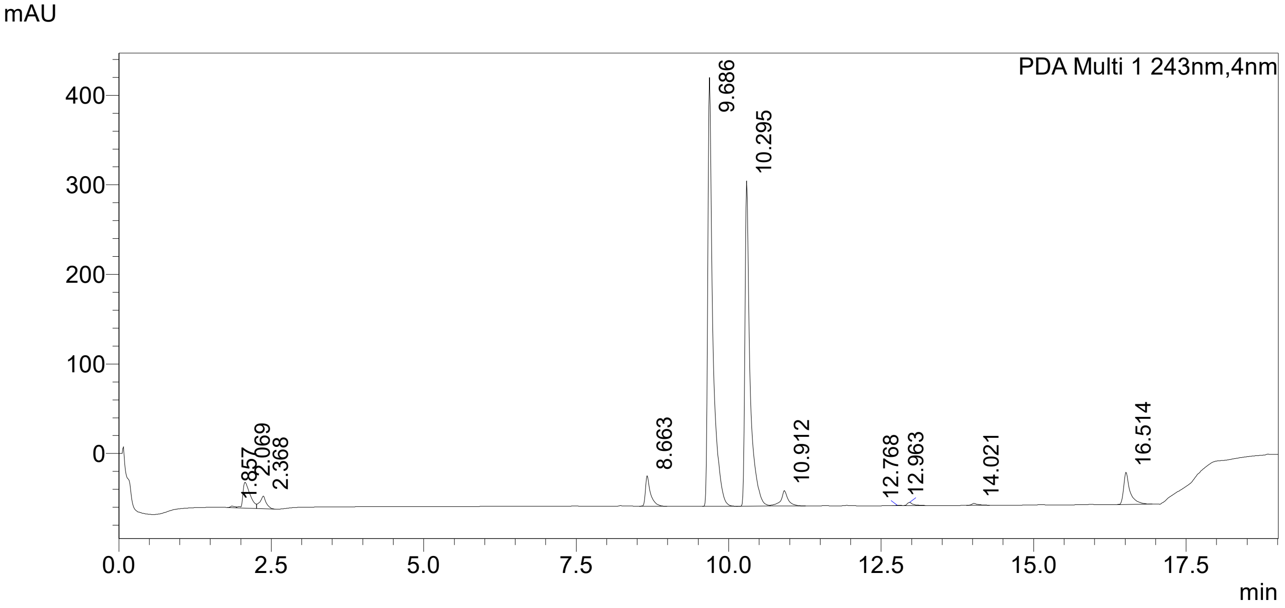

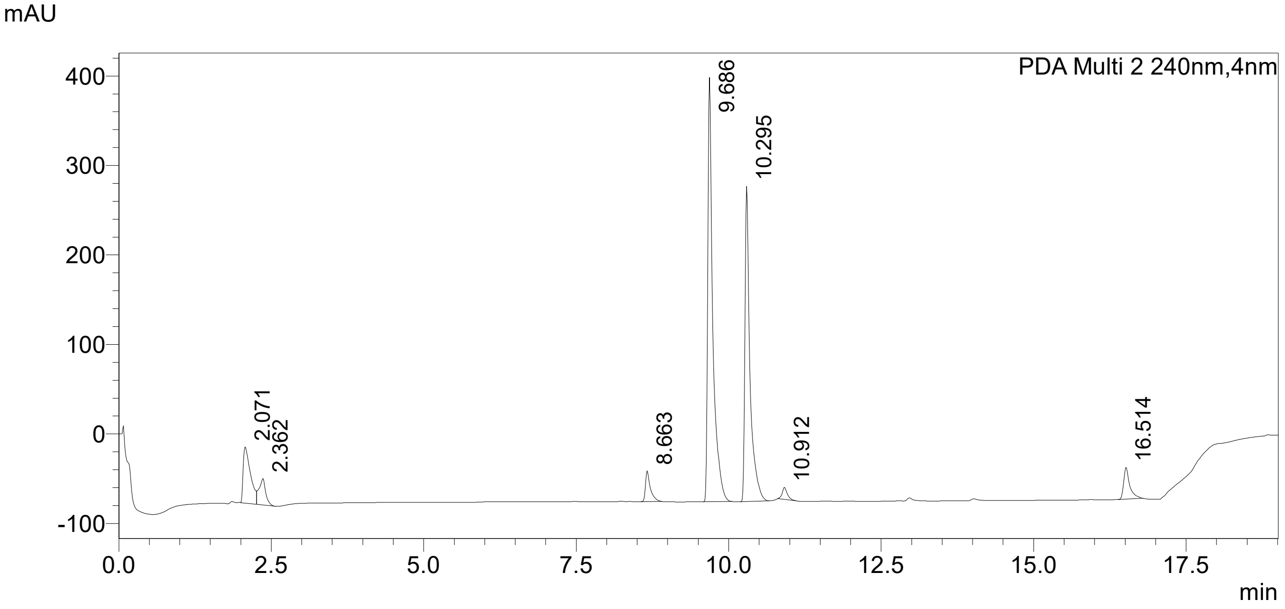

<Peak Table>

PDA Ch1 243nm

Peak#	Ret. Time	Area	Height	Conc.	Unit	Mark	Name
1	1.857	10162	1806	0.000			
2	2.069	243078	29013	0.000		V	
3	2.368	101415	14263	0.000		V	
4	8.663	194752	33896	0.000			
5	9.686	2790186	478676	266.505	uM		MHET
6	10.295	2126288	363040	209.850	uM	V	BHET
7	10.912	143272	17191	0.000		V	
8	12.768	3136	477	0.000			
9	12.963	23687	3576	0.000		V	
10	14.021	15462	2030	0.000			
11	16.514	263938	35788	0.000			
Total		5915375	979755				

PDA Ch2 240nm

Peak#	Ret. Time	Area	Height	Conc.	Unit	Mark	Name
1	2.071	540237	62890	0.000			
2	2.362	218772	29465	0.000		V	
3	8.663	191817	34078	15.052	uM		TPA
4	9.686	2756869	474156	0.000			
5	10.295	2048640	352200	0.000			
6	10.912	80153	13413	0.000			
7	16.514	249390	35612	0.000			
Total		6085878	1001813				
