# Supplementary material for: A flexible kinetic assay efficiently sorts prospective biocatalysts for PET plastic subunit hydrolysis
Source: RSC Adv. 2022 Mar 14;12(13):8119–30. doi: 10.1039/d2ra00612j (PMC8982334; doi:10.1039/d2ra00612j)
Supplement: RA-012-D2RA00612J-s033 [file RA-012-D2RA00612J-s033.pdf]

SHIMADZU LabSolutions

Analysis Report

<Sample Information>

Sample Name	: E1 50C		
Sample ID	:		
Data Filename	E1 50C _017.lcd		
Method Filename	: MHET_BHET_rpamide_060721.lcm		
Batch Filename	: BHET_Colorimetric_50C_pH8_plate1.lcb		
Vial #	: 4-7	Sample Type	: Unknown
Injection Volume	: 10 uL		
Date Acquired	: 8/30/2021 5:44:29 PM	Acquired by	: System Administrator
Date Processed	: 9/3/2021 8:51:55 AM	Processed by	: System Administrator

<Chromatogram>

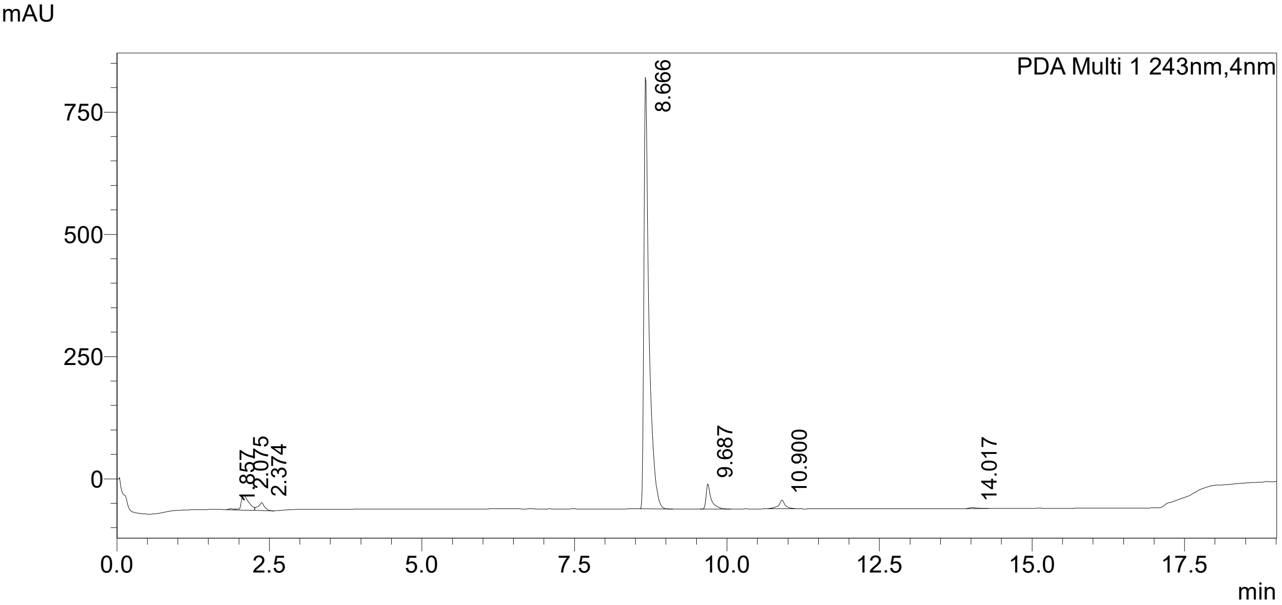

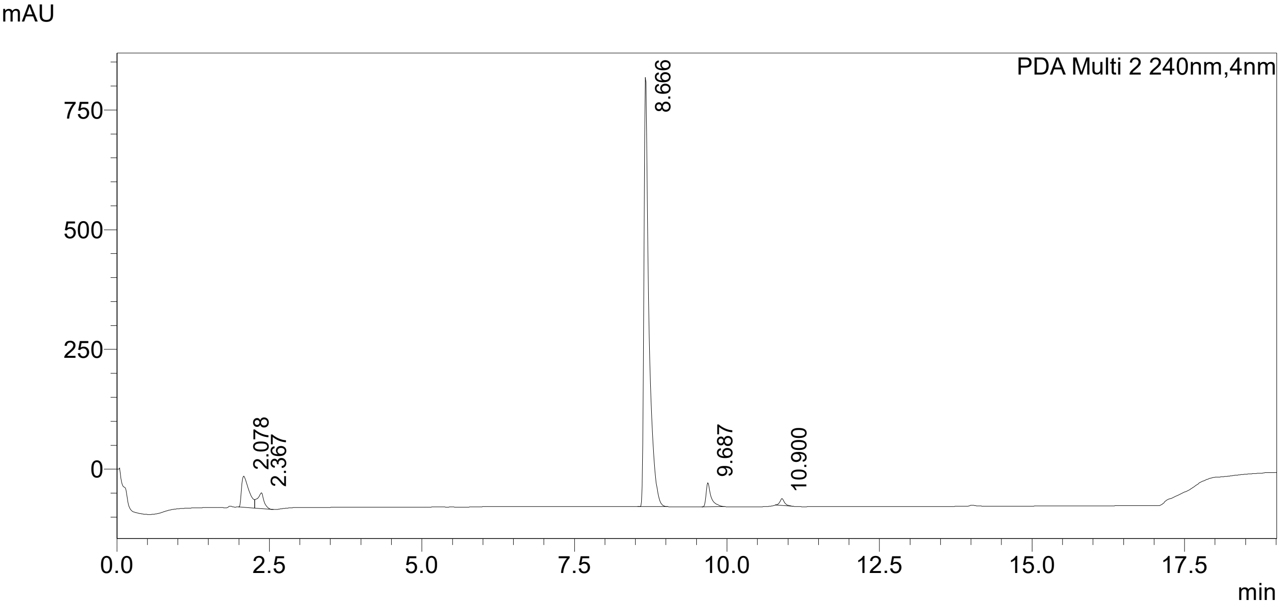

<Peak Table>

PDA Ch1 243nm

Peak#	Ret. Time	Area	Height	Conc.	Unit	Mark	Name
1	1.857	15172	2520	0.000			
2	2.075	266339	30187	0.000		V	
3	2.374	117023	16474	0.000		V	
4	8.666	5148691	882369	0.000			
5	9.687	296793	50979	24.228	uM		MHET
6	10.900	138798	18203	0.000			
7	14.017	18494	2406	0.000			
Total		6001308	1003138				

PDA Ch2 240nm

Peak#	Ret. Time	Area	Height	Conc.	Unit	Mark	Name
1	2.078	577192	65103	0.000			
2	2.367	249655	33444	0.000		V	
3	8.666	5210057	896168	503.702	uM		TPA
4	9.687	286485	50275	0.000			
5	10.900	77334	14308	0.000			
Total		6400722	1059299				
