# Supplementary material for: A flexible kinetic assay efficiently sorts prospective biocatalysts for PET plastic subunit hydrolysis
Source: RSC Adv. 2022 Mar 14;12(13):8119–30. doi: 10.1039/d2ra00612j (PMC8982334; doi:10.1039/d2ra00612j)
Supplement: RA-012-D2RA00612J-s034 [file RA-012-D2RA00612J-s034.pdf]

SHIMADZU LabSolutions

Analysis Report

<Sample Information>

Sample Name	: E10 50C		
Sample ID	:		
Data Filename	: E10 50C _023.lcd		
Method Filename	: MHET_BHET_rpamide_060721.lcm		
Batch Filename	: BHET_Colorimetric_50C_pH8_plate1.lcb		
Vial #	: 4-13	Sample Type	: Unknown
Injection Volume	: 10 uL		
Date Acquired	: 8/30/2021 7:40:40 PM	Acquired by	: System Administrator
Date Processed	: 9/3/2021 8:52:06 AM	Processed by	: System Administrator

<Chromatogram>

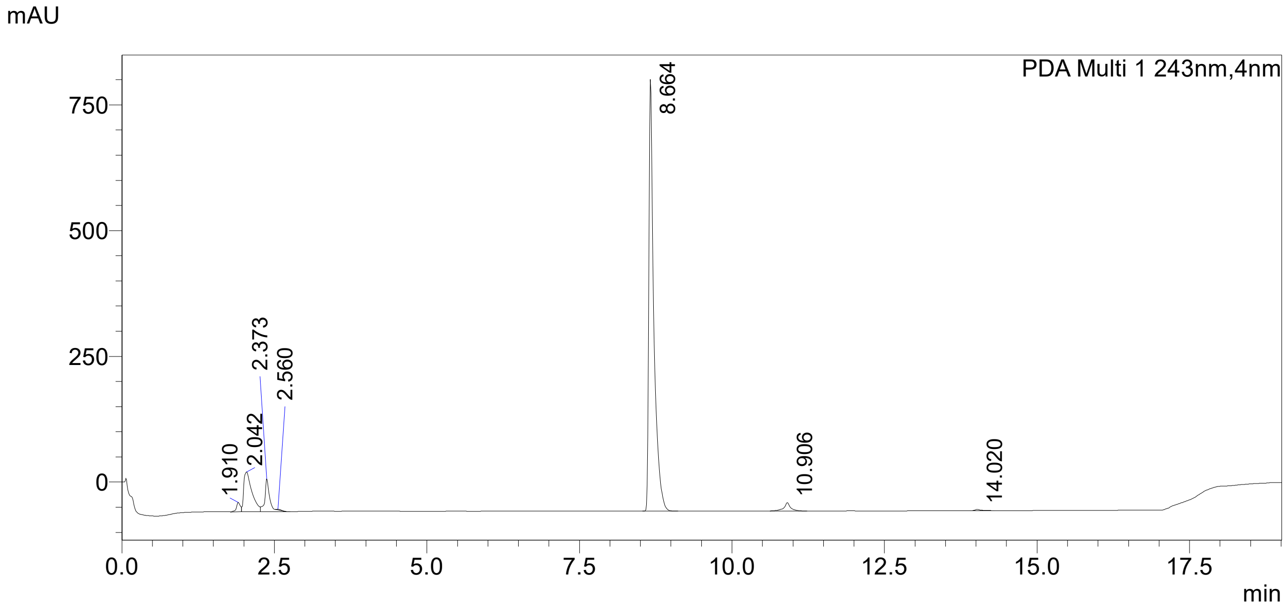

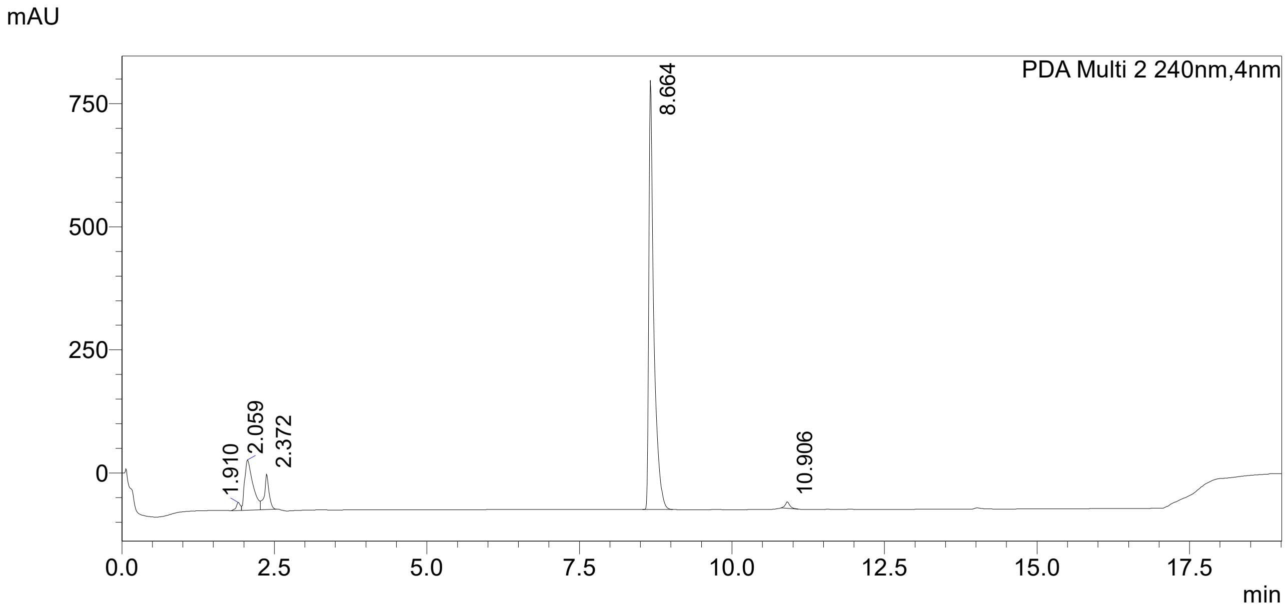

<Peak Table>

PDA Ch1 243nm

Peak#	Ret. Time	Area	Height	Conc.	Unit	Mark	Name
1	1.910	86983	18624	0.000			
2	2.042	747310	79044	0.000		V	
3	2.373	372008	65150	0.000		SV	
4	2.560	6628	1544	0.000		T	
5	8.664	4973833	858277	0.000			
6	10.906	126332	16601	0.000			
7	14.020	19113	2455	0.000			
Total		6332207	1041695				

PDA Ch2 240nm

Peak#	Ret. Time	Area	Height	Conc.	Unit	Mark	Name
1	1.910	79848	16357	0.000			
2	2.059	977905	102118	0.000		V	
3	2.372	385443	71554	0.000		V	
4	8.664	5031486	871142	485.814	uM		TPA
5	10.906	68566	12919	0.000			
Total		6543249	1074090				
