# Supplementary material for: A flexible kinetic assay efficiently sorts prospective biocatalysts for PET plastic subunit hydrolysis
Source: RSC Adv. 2022 Mar 14;12(13):8119–30. doi: 10.1039/d2ra00612j (PMC8982334; doi:10.1039/d2ra00612j)
Supplement: RA-012-D2RA00612J-s035 [file RA-012-D2RA00612J-s035.pdf]

SHIMADZU LabSolutions

Analysis Report

<Sample Information>

Sample Name	: E11 50C		
Sample ID	:		
Data Filename	: E11 50C _026.lcd		
Method Filename	: MHET_BHET_rpamide_060721.lcm		
Batch Filename	: BHET_Colorimetric_50C_pH8_plate1.lcb		
Vial #	: 4-16	Sample Type	: Unknown
Injection Volume	: 10 uL		
Date Acquired	: 8/30/2021 8:38:47 PM	Acquired by	: System Administrator
Date Processed	: 9/3/2021 8:52:12 AM	Processed by	: System Administrator

<Chromatogram>

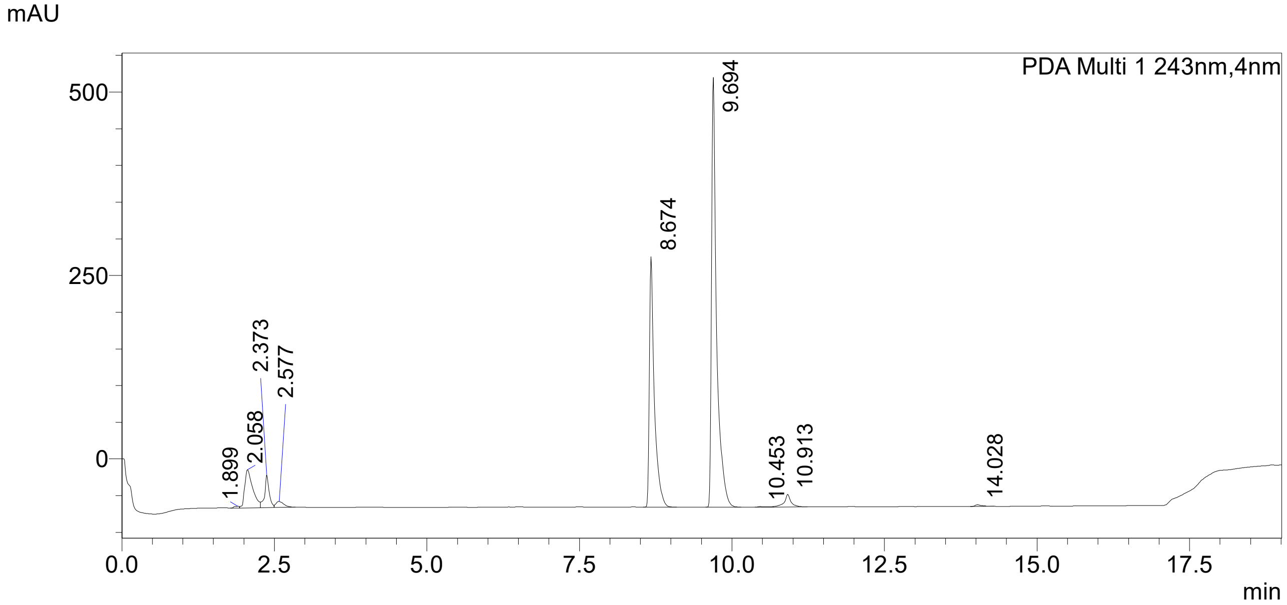

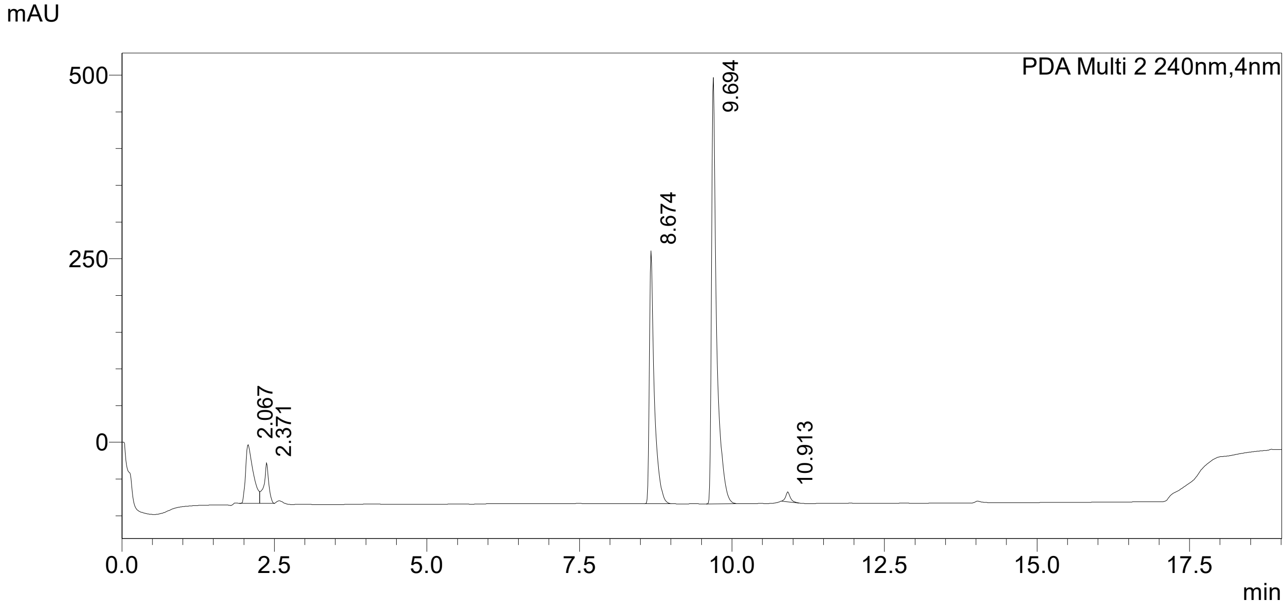

<Peak Table>

PDA Ch1 243nm

Peak#	Ret. Time	Area	Height	Conc.	Unit	Mark	Name
1	1.899	15404	2411	0.000			
2	2.058	487021	52238	0.000		V	
3	2.373	228065	43721	0.000		V	
4	2.577	71826	8204	0.000		V	
5	8.674	1957407	341293	0.000			
6	9.694	3426832	585637	325.478	uM		MHET
7	10.453	5372	560	-2.186	uM		BHET
8	10.913	132546	17279	0.000		V	
9	14.028	17966	2331	0.000			
Total		6342440	1053674				

PDA Ch2 240nm

Peak#	Ret. Time	Area	Height	Conc.	Unit	Mark	Name
1	2.067	719636	79902	0.000			
2	2.371	308639	54799	0.000		V	
3	8.674	1972137	344687	187.113	uM		TPA
4	9.694	3390125	580604	0.000			
5	10.913	73217	13583	0.000			
Total		6463753	1073575				
