# Supplementary material for: A flexible kinetic assay efficiently sorts prospective biocatalysts for PET plastic subunit hydrolysis
Source: RSC Adv. 2022 Mar 14;12(13):8119–30. doi: 10.1039/d2ra00612j (PMC8982334; doi:10.1039/d2ra00612j)
Supplement: RA-012-D2RA00612J-s036 [file RA-012-D2RA00612J-s036.pdf]

SHIMADZU LabSolutions

Analysis Report

<Sample Information>

Sample Name	: E2 50C		
Sample ID	:		
Data Filename	: E2 50C _020.lcd		
Method Filename	: MHET_BHET_rpamide_060721.lcm		
Batch Filename	: BHET_Colorimetric_50C_pH8_plate1.lcb		
Vial #	: 4-10	Sample Type	: Unknown
Injection Volume	: 10 uL		
Date Acquired	: 8/30/2021 6:42:36 PM	Acquired by	: System Administrator
Date Processed	: 9/3/2021 8:52:01 AM	Processed by	: System Administrator

<Chromatogram>

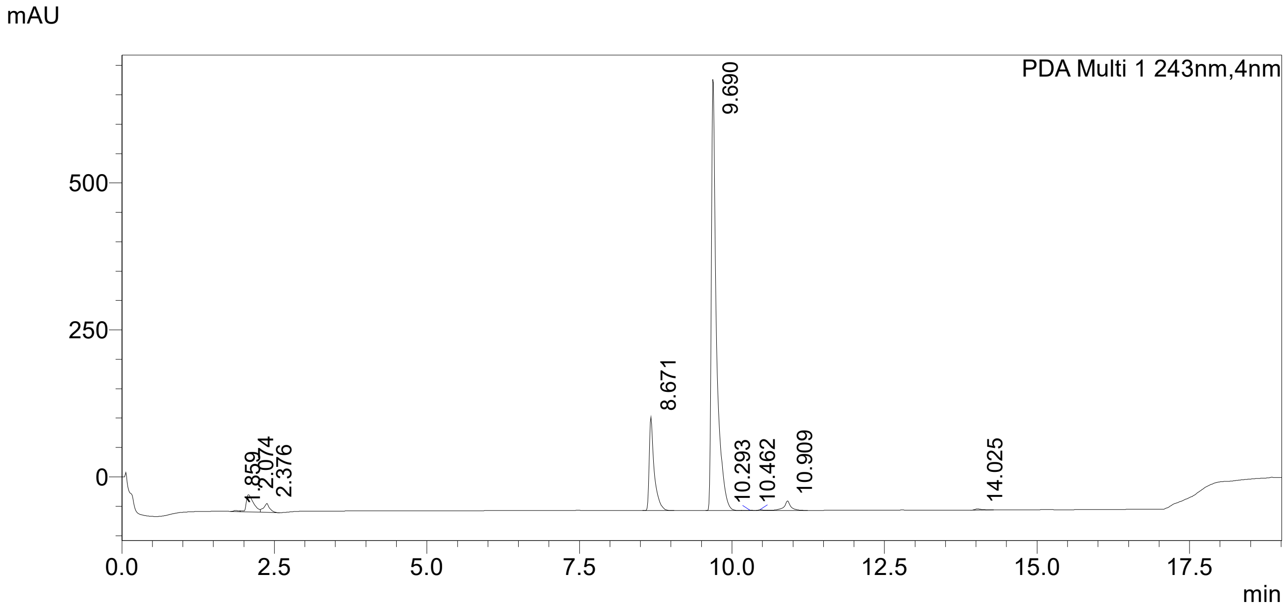

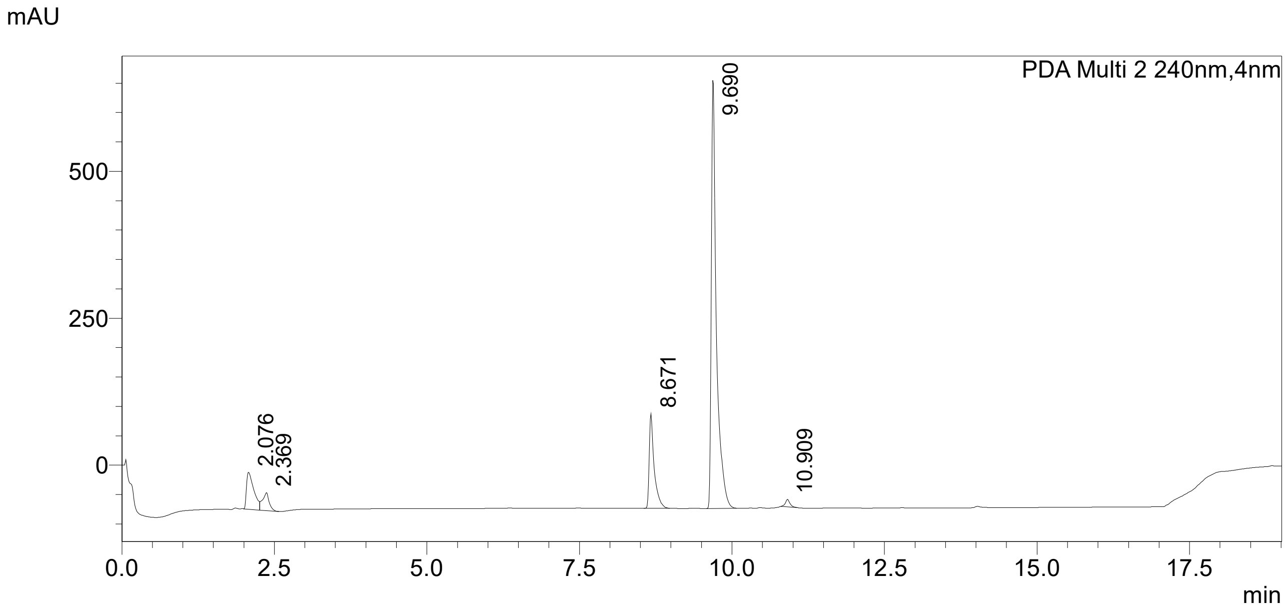

<Peak Table>

PDA Ch1 243nm

Peak#	Ret. Time	Area	Height	Conc.	Unit	Mark	Name
1	1.859	11648	1922	0.000			
2	2.074	247786	29187	0.000		V	
3	2.376	102414	15081	0.000		V	
4	8.671	910509	159148	0.000			
5	9.690	4332931	733481	409.411	uM		MHET
6	10.293	3430	613	-2.372	uM	V	BHET
7	10.462	8170	1479	0.000		V	
8	10.909	129820	16397	0.000		V	
9	14.025	16418	2138	0.000			
Total		5763127	959444				

PDA Ch2 240nm

Peak#	Ret. Time	Area	Height	Conc.	Unit	Mark	Name
1	2.076	538554	62973	0.000			
2	2.369	227537	30721	0.000		V	
3	8.671	915382	160578	82.732	uM		TPA
4	9.690	4289580	728154	0.000			
5	10.909	68044	12703	0.000			
Total		6039096	995128				
