# Supplementary material for: A flexible kinetic assay efficiently sorts prospective biocatalysts for PET plastic subunit hydrolysis
Source: RSC Adv. 2022 Mar 14;12(13):8119–30. doi: 10.1039/d2ra00612j (PMC8982334; doi:10.1039/d2ra00612j)
Supplement: RA-012-D2RA00612J-s038 [file RA-012-D2RA00612J-s038.pdf]

Electronic Supplementary Material (ESI) for RSC Advances.
This journal is © The Royal Society of Chemistry 2022

Supplementary Data for:
A flexible kinetic assay efficiently sorts prospective biocatalysts for polyester subunit hydrolysis

Jessica L. Beech, Rita Clare, William M. Kincannon, Erika Erickson, John E. McGeehan, Gregg T. Beckham, Jennifer L. DuBois

Contents:

Figures

Figure S1. Phthalein and related indicators with potential assay applicability

Figure S2. SDS-PAGE and densitometry illustrating partial purification of PET esterases 1-9

Figure S3. SDS-PAGE illustrating approximate molecular weights of commercially acquired esterases 10-18

Figure S4. Temperature stability of phenol red

Figure S5. Qualitative colorimetric assay using freeze dried cells

Figure S6. HPLC traces illustrating retention times of standards and analysis of final reaction products.

Figure S7. The H+ sensing assay is appropriate for monitoring hydrolysis of solid phase amorphous PET film.

Figure S8. UV/vis spectra and determination of extinction coefficients for PR

Figure S9. UV/vis spectra and determination of extinction coefficients for BTB

Figure S10. Progress of reaction curves monitored for the full set of 18 enzymes over 24 hours

Figure S11. Sequence alignment of *B. subtilis* esterase and *I. sakaiensis* PETase

Figure S12. Sequence alignment of *G. stearothermophilus* esterase and *I. sakaiensis* PETase

Tables

Table S1. Esterases with previously established activity against solid phase PET used in this study

Table S2. Esterases obtained commercially (Sigma)

Table S3. Specific activities for esterases 1-18 measured colorimetrically within the first hour of reaction with BHET as substrate

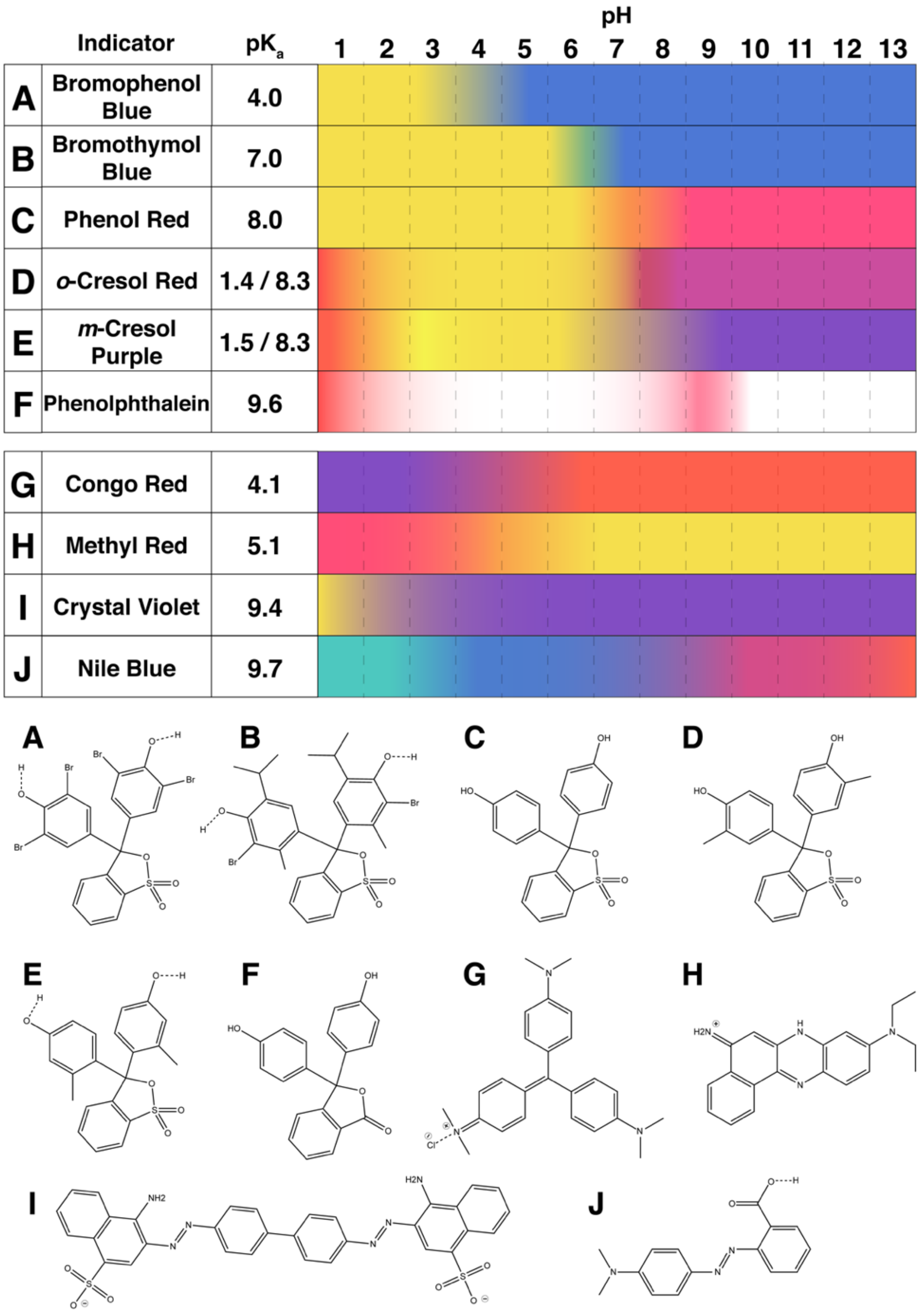

Figure S1. Phthalein (A-F) and other (G-J) water soluble pH indicator dyes, their pK_as, and detection ranges. Many of the indicators shown here are known for their chemical stability and high ε values, making them favorable for use as indicators in sensitive assay measurements. When used with a buffer of equivalent pK_a, these dyes could potentially be used in place of bromothymol blue (**B**) and phenol red (**C**), tailoring the pH detection range of the assay described in this work for other applications. For a more extensive list of these dyes, see supplemental reference 1.[1, 2]

Figure S2

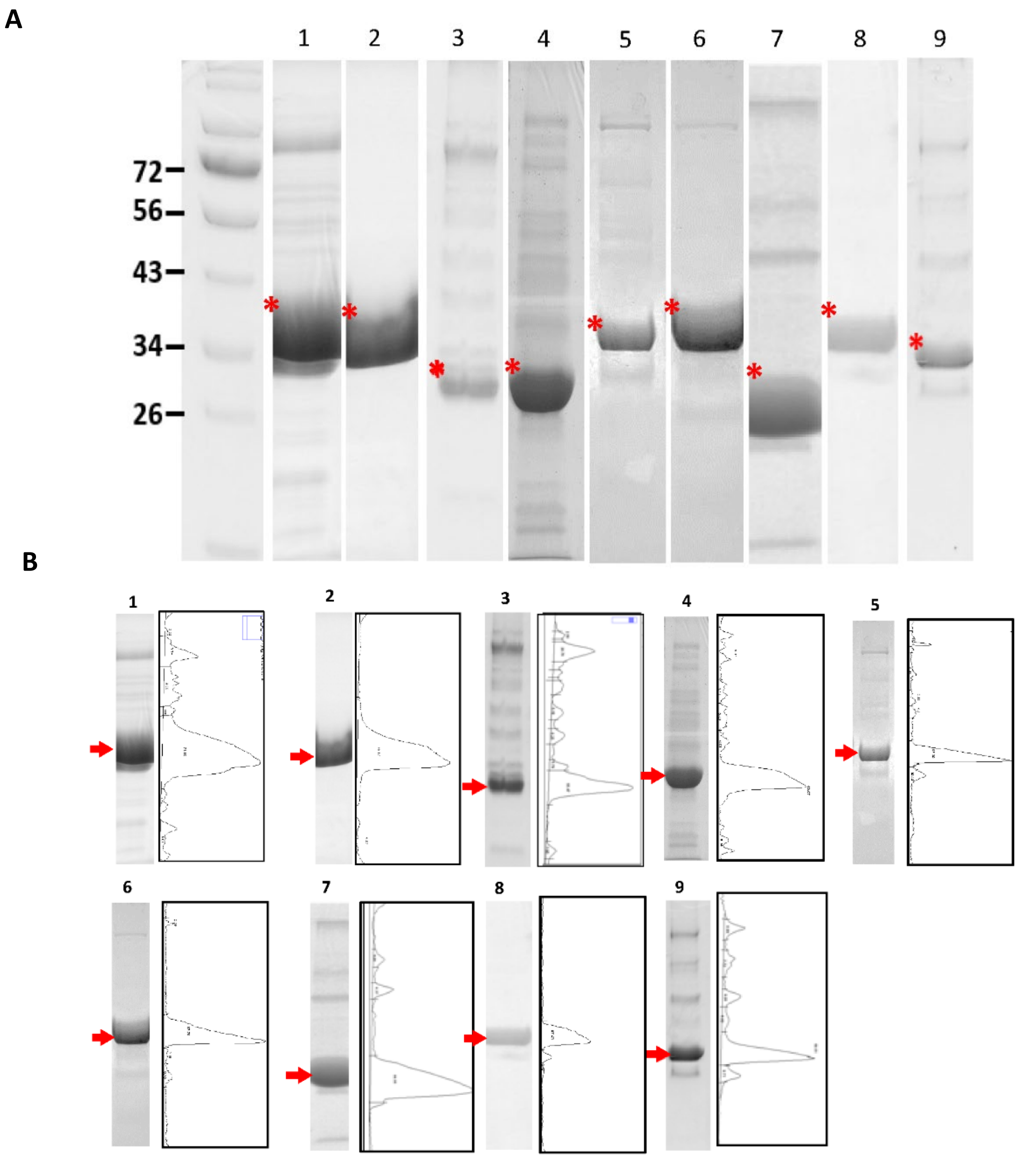

Figure S2. A. Partially purified His6-tagged PET hydrolase enzymes were separated and visualized on a 12% acrylamide SDS PAGE gel. (A) A molecular weight marker (M, Fisher BioReagents, EZ-Run Pre-Stained Rec Protein ladder (Cat# BP3603-1) is shown in the first lane, with 10 μg of purified PET hydrolase protein samples in subsequent lanes. Protein bands were observed after staining with Coomassie blue staining dye. Images were acquired using a flatbed scanner (Cannon LiDE 210). (B) Densitometry was used to quantify the relative amounts of protein in each band. Proteins corresponding to the expected correct molecular weight for the respective PET esterase are indicated. The band density was analyzed using ImageJ (NIH), a public domain program from the National Institutes of Health that allows image processing. Band densities relative to the overall density of proteins in each lane were used to adjust protein stock concentrations (measured by Bradford assay) to reflect the fraction of target protein in each.

Figure S3

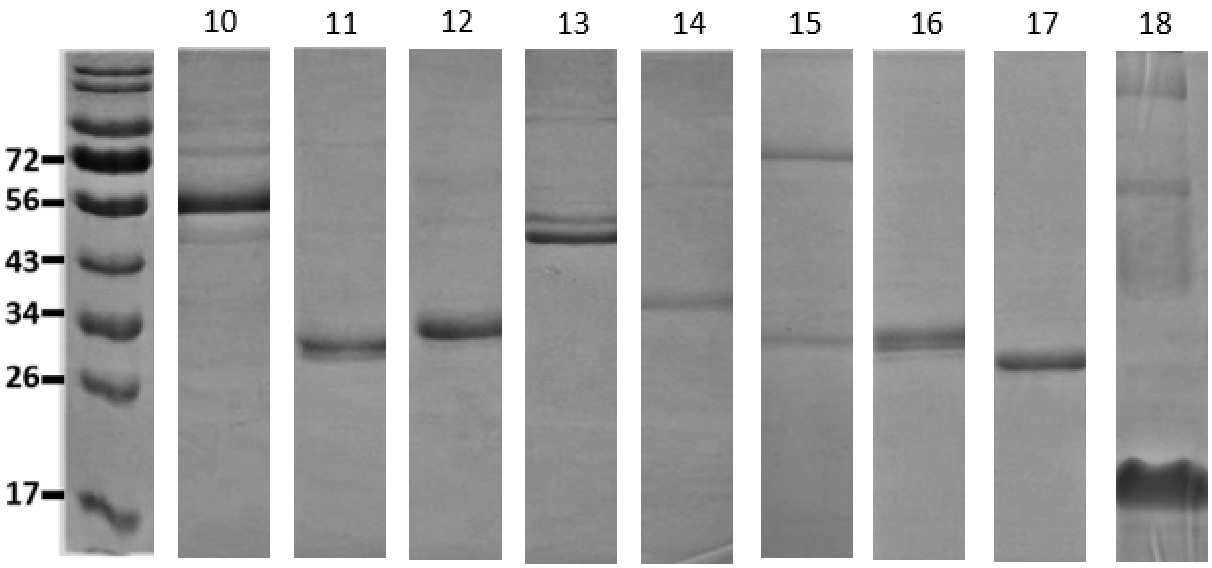

Figure S3. The commercially obtained esterases were separated and visualized on a 12% acrylamide SDS PAGE gel. The first lane shows the protein molecular weight size marker (Fisher BioReagents, EZ-Run Pre-Stained Rec Protein ladder, Cat# BP3603-1). Protein bands were observed after staining with PageBlue Protein Staining Solution (Thermo Scientific Cat# 24620). Enzymes are marked as follows: 10) 1 μg of *B. stearothermophilus* esterase, 11) 0.5 μg of *B. subtillis* carboxylesterase, 12) 0.5 μg of *A. orzyae* lipase, 13) 10 μg of *C. antarctica* lipase A, 14) 10 μg of *C. antarctica* lipase B, 15) 10 μg of *R. oryzae* lipase, 16) 10 μg of *A. oryzae* phospholipase A1, 17) 1 μg of *Pseudomonas sp*. lipoprotein lipase, 18) 10 μg of bovine pancreas phospholipase A2.

Figure S4.

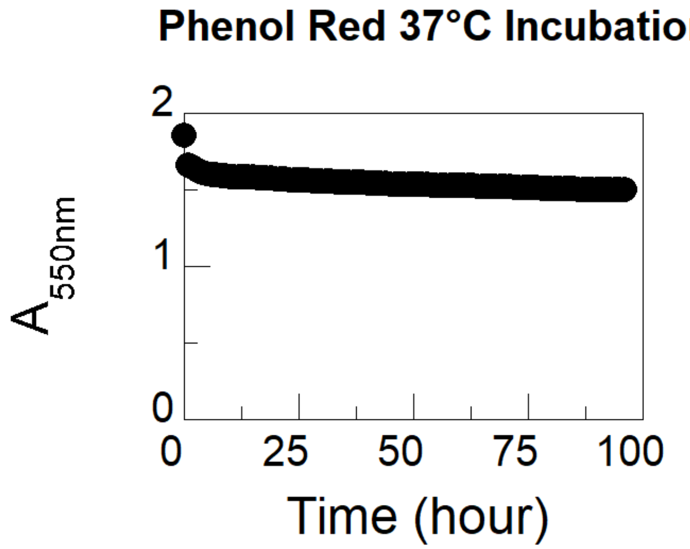

Figure S4. Phenol red was evaluated for temperature stability. Three wells of a 96 well plate were filled to 300 μL with a 0.1 mM phenol red, 5 mM HEPPS buffer (pH 8.0) solution. The plate was sealed with a Bio-Rad B-seal to prevent evaporation and placed in a Thermo Scientific VarioLux plate reader incubating at 37°C. UV/visible measurements at 550 nm were made every hour for 97 hours. The resulting absorbance change was plotted as a function of time. An approximately 10% absorbance drop occurred in the first hour of incubation as the solution re-equilibrated to 37°C after cooling during the preparation of the plate. Subsequent absorbance changes were small and linear over the >90 h duration of the measurement. Similar assessments of the stability of BB (not shown) suggested that both indicators stably maintain their expected absorbances over time.

Figure S5

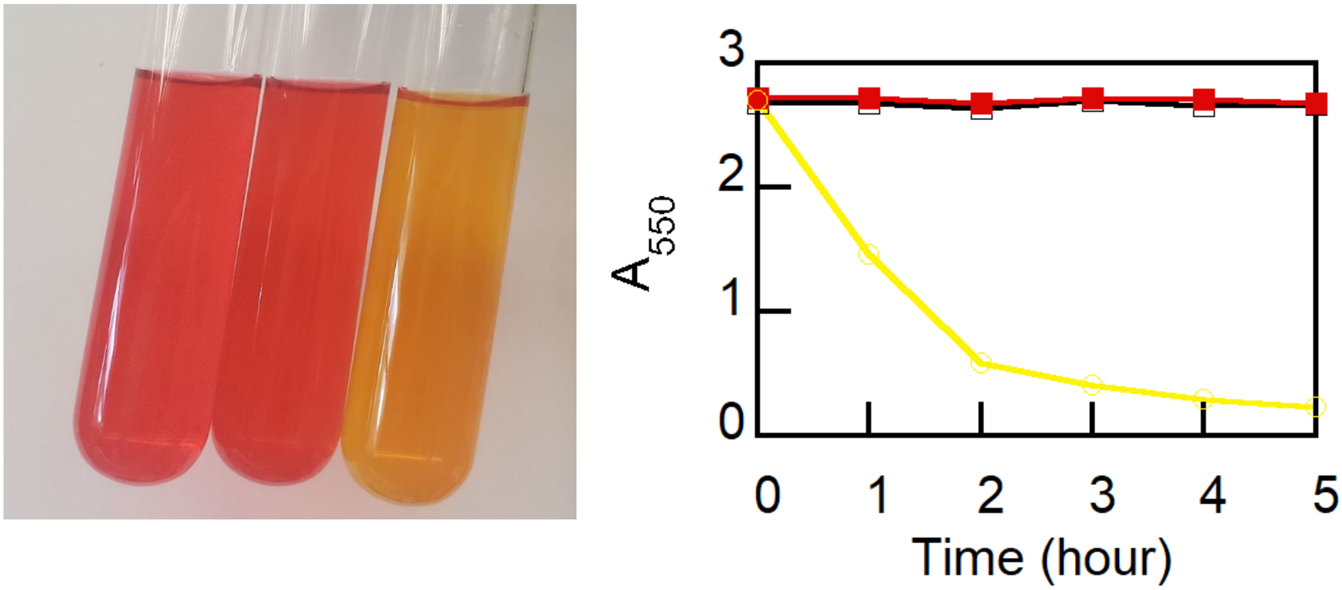

Figure S5. Enzymatic activity was detected in whole, lyophilized cells containing expressed esterases. *E. coli* Lemo (DE3) cells were grown in LB media overnight at 20°C. The same cell line harboring a pET-vector for expression of enzyme 1 (see main text methods) were induced with 1 mM IPTG and grown overnight in LB media at 20°C. 1 mL of each culture was harvested and immediately lyophilized. Lyophilized cells were resuspended in 1 mL 20 mM Tris, 150 mM NaCl buffer, pH 7.0 in culture tubes. Cells were then colorimetrically monitored for ester hydrolysis following addition of 2 mM tributyrin (a triester) and 0.1 mM phenol red in 5 mM HEPPS buffer, pH 8.0 (final culture volume: 10 mL). Reactions were incubated at room temperature, with A_{550} measured every hour for five hours (left). Image of culture tubes taken three hours after induction, showing left to right: no-enzyme control, untransformed control, and cells with induced expression of enzyme 1. The change from reddish orange to a gold-orange huge corresponds to acidification of the PR indicator. (right) Absorbance plotted over time for no-enzyme control (black), untransformed control (red), and cells with induced expression of enzyme 1. These results demonstrate that hydrolysis activity against this model ester is clearly discernable by the naked eye and via simple UV/visible absorbance measurements in a short time frame.

Figure S6

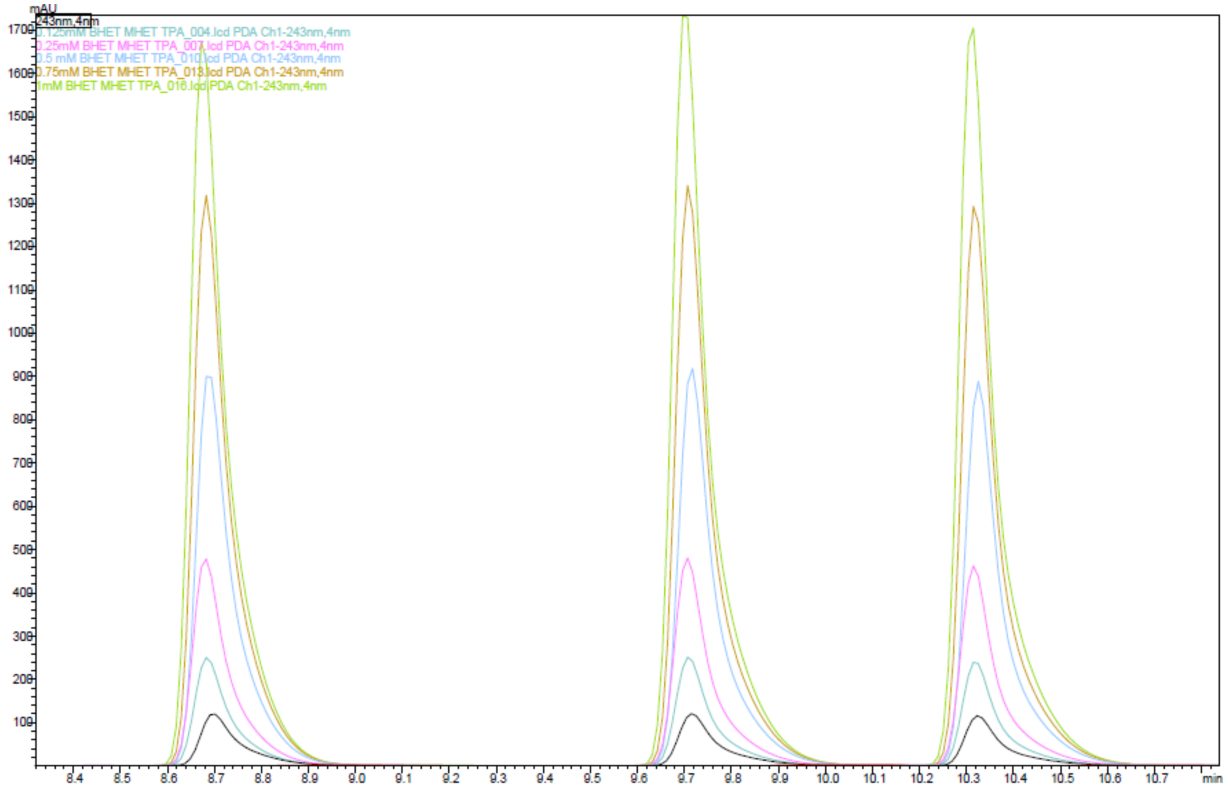

Figure S6. HPLC traces illustrating peak shapes and retention times of standards and analysis of final reaction products. The mobile phase used to separate the analytes of interest was water (A)/acetonitrile (B), each with 0.1% trifluoroacetic acid Reaction components were separated at a 1.5 mL min-1: column wash for 3 min with 5%B, 5% to 17% B gradient from 3 to 6 min, 17% to 25% B from 6 to 9 min, 25% to 40% B from 9 to 15 minutes, a wash of 100% B for 2 min. The column was re-equilibrated between samples with 5% B, 2 min. A DAD wavelength of either 243 or 240nm was used to monitor analytes eluding at retention times of 8.7 minutes for TPA, 9.7 minutes for MHET, and 10.3 minutes for BHET. Shown is a standard curve with concentrations of 60μM (black), 125μM (teal), 250μM (pink), 500μM (blue), 750μM (brown), and 1mM (green).

Figure S7

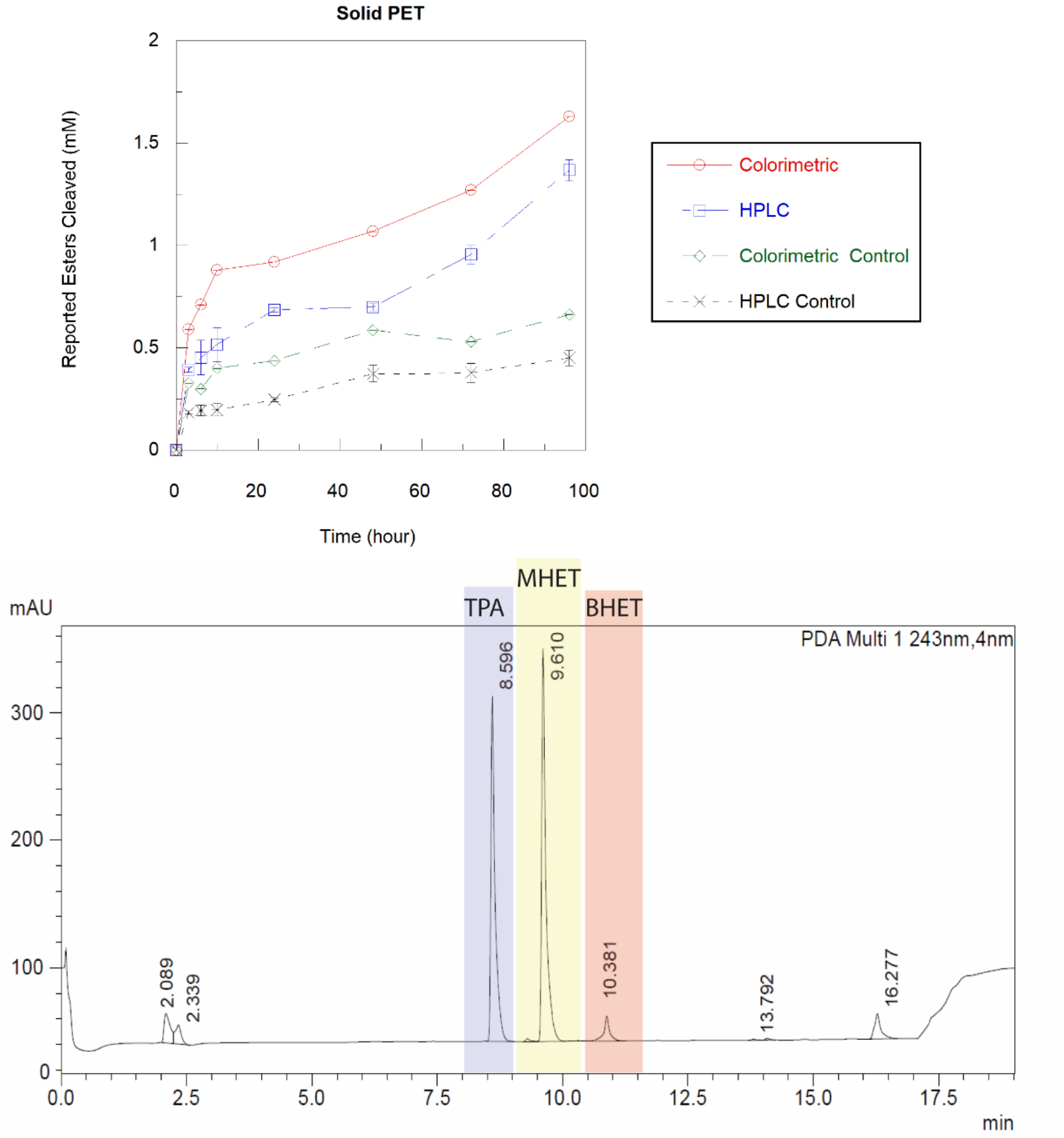

Figure S7. The H+ sensing assay is appropriate for monitoring hydrolysis of solid phase amorphous PET film and consistent with HPLC analyses. Top: *I. sakaiensis* PETase (enzyme 2) was allowed to react with solid amorphous PET film (Goodfellows, 252-144-75). Each well of a 96 well plate contained 0.06 g solid amorphous PET film in a solution of 5 mM HEPPS, pH 8.0, 0.1 mM phenol red, 10 mM $CaCl_2$, and 10% glycerol. Reactions were initiated by the addition of 0.01 mM *I. sakaiensis* PETase and incubated with light shaking at 37 °C. Absorbance at 550 nm was continuously monitored for the reaction over time. Reactions were incubated in additional wells for discontinuous monitoring by HPLC. These were quenched with 1 volume ice cold methanol at 0, 3, 6, 10, 24, 48, 72, and 96 hours, quenched samples were

thawed, centrifuged to remove protein, and analyzed by HPLC following the method used for the BHET reactions. Results show remarkable qualitative and quantitative agreement between the colorimetric and HPLC based methods. Bottom: HPLC chromatogram after 96 hours solid PET incubation with *I. sakaiensis* PETase, labeled. Retention times at 8.5 minutes, 9.6 minutes, and 10.3 minutes correspond to TPA, MHET, and BHET respectively. Peaks at 2.1 and 2.3 minutes are solvent contaminants eluted by a 95% water column wash. These peaks are consistently seen in all HPLC chromatograms presented here. The peak at 16.3 minutes is an unidentified peak unique to the PET substrate sample.

Figure S8

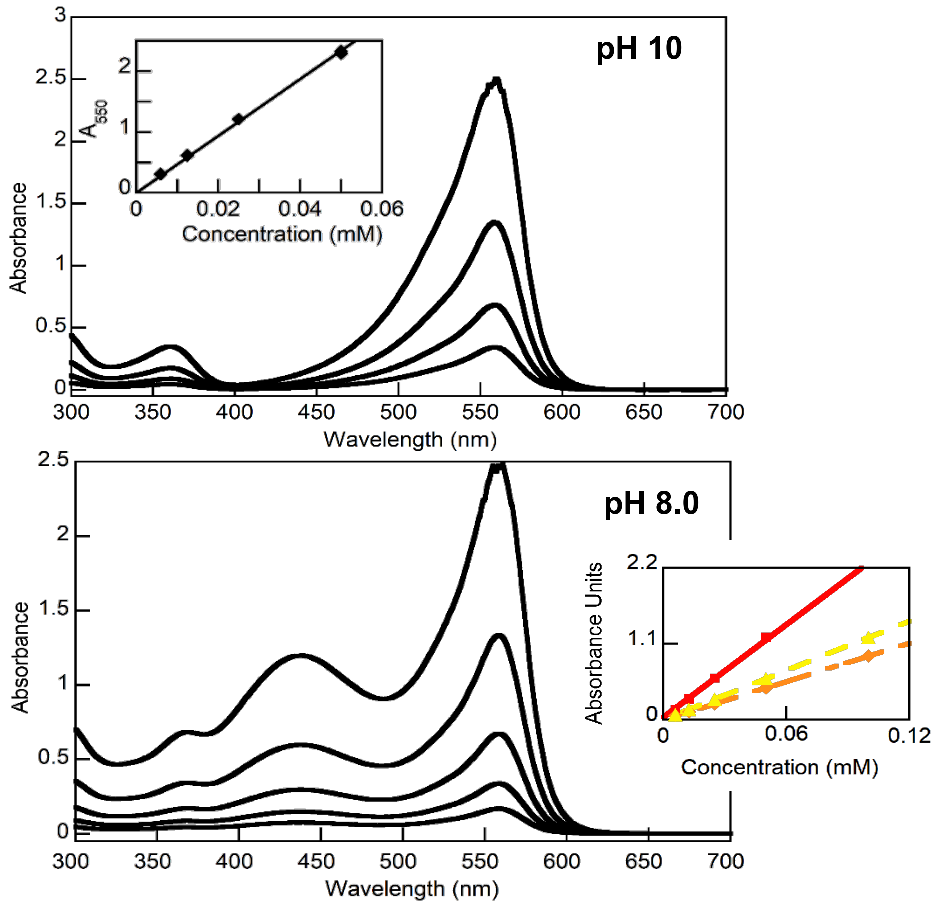

Figure S8. Extinction coefficients of the acidic and alkaline forms of phenol red were determined from UV/visible absorbance spectra. (top) Spectra were measured for 50, 25, 12.5, and 6.3 μM phenol red solutions diluted from a 20 mM stock into 5 mM HEPPS buffer at pH 10.0 (Top), and for 100, 50, 25, 12.5, and 6.3 μM solutions at pH 8.0, the pKa (bottom). An extinction coefficient for the alkaline form was determined by fitting a line to the triplicate measurements of the concentration dependence of the absorbance at 550 nm for the alkaline species at pH 10 (inset), where slope = $\varepsilon_{alkaline}$ = 48,000 M^{-1} cm^{-1}. (bottom) The concentration dependence was measured in the same way at pH = pKa = 8.0 for both the alkaline (550 nm, red line) and acidic species ($\lambda_{max\ acid}$ 434 nm, yellow), and at the isosbestic point ($\lambda_{isobestic}$ = 480 nm, orange). The alkaline and acidic forms are present at equal concentrations at the pKa, the apparent epsilon values measured from the slope of the graphed points (inset) were doubled: ε_{acidic} = 23,000 M^{-1} cm^{-1}, $\varepsilon_{isobestic}$ = 18,300 M^{-1} cm^{-1}, $\varepsilon_{alkaline}$ = 47,000 M^{-1} cm^{-1}. The = $\varepsilon_{alkaline}$ = 48,000 M^{-1} cm^{-1} was used in this study.

Figure S9.

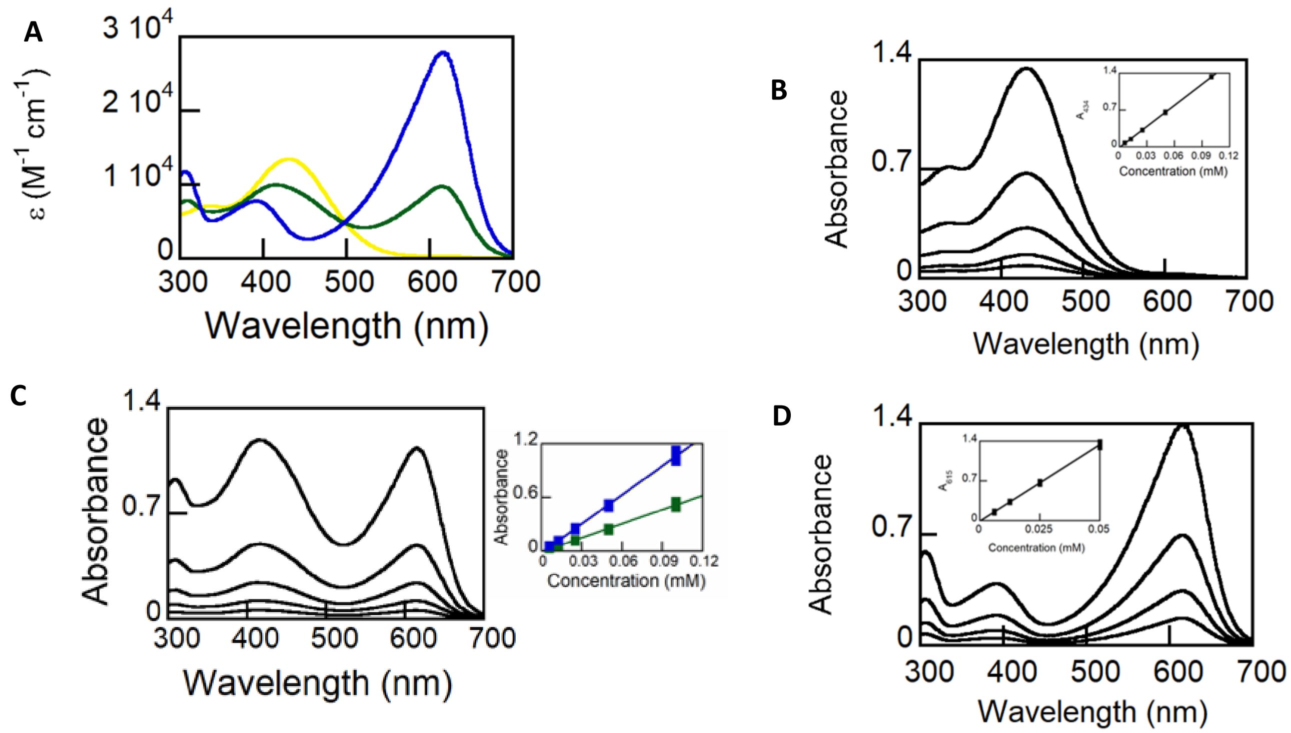

Figure S9. Extinction coefficients of the acidic and alkaline forms of bromothymol blue (BB) were determined from UV/visible absorbance spectra. (A) Spectra for acidic (yellow) and alkaline (blue) forms of BB as well as a 50:50 mixture of the two (green) were excerpted from the data measured in the following panels. Spectra were measured for 100, 50, 25, 12.5, and 6.3 μM bromothymol blue (BTB) solutions diluted from a 20 mM stock into 5 mM BES buffer at (B) pH 5.1 (D) 9.1, and (C) pH 7.1, the pK_a. An extinction coefficient for the alkaline form was determined by fitting a line to the concentration versus the absorbance at 615 nm for the alkaline species at pH 9.1 (inset, plot D), where slope = $\varepsilon_{alkaline}$ = 24,000 M^{-1} cm^{-1}. The concentration dependence was measured in the same way at pH = pK_a = 7.1 for both the alkaline (615 nm, blue line) and acidic species ($\lambda_{max\ acid}$ 434 nm, yellow), and at the isosbestic point ($\lambda_{isobestic}$ = 500 nm, green) (inset, plot C). Because the alkaline and acidic forms are present at equal concentrations at the pKa, ε_{acidic} = 21,000 M^{-1} cm^{-1}, $\varepsilon_{isobestic}$ = 5,200 M^{-1} cm^{-1}, $\varepsilon_{alkaline}$ = 21,000 M^{-1} cm^{-1}. The value for $\varepsilon_{alkaline}$ = 24,000 M^{-1} cm^{-1} measured in plot D was used in this study.

Figure S10

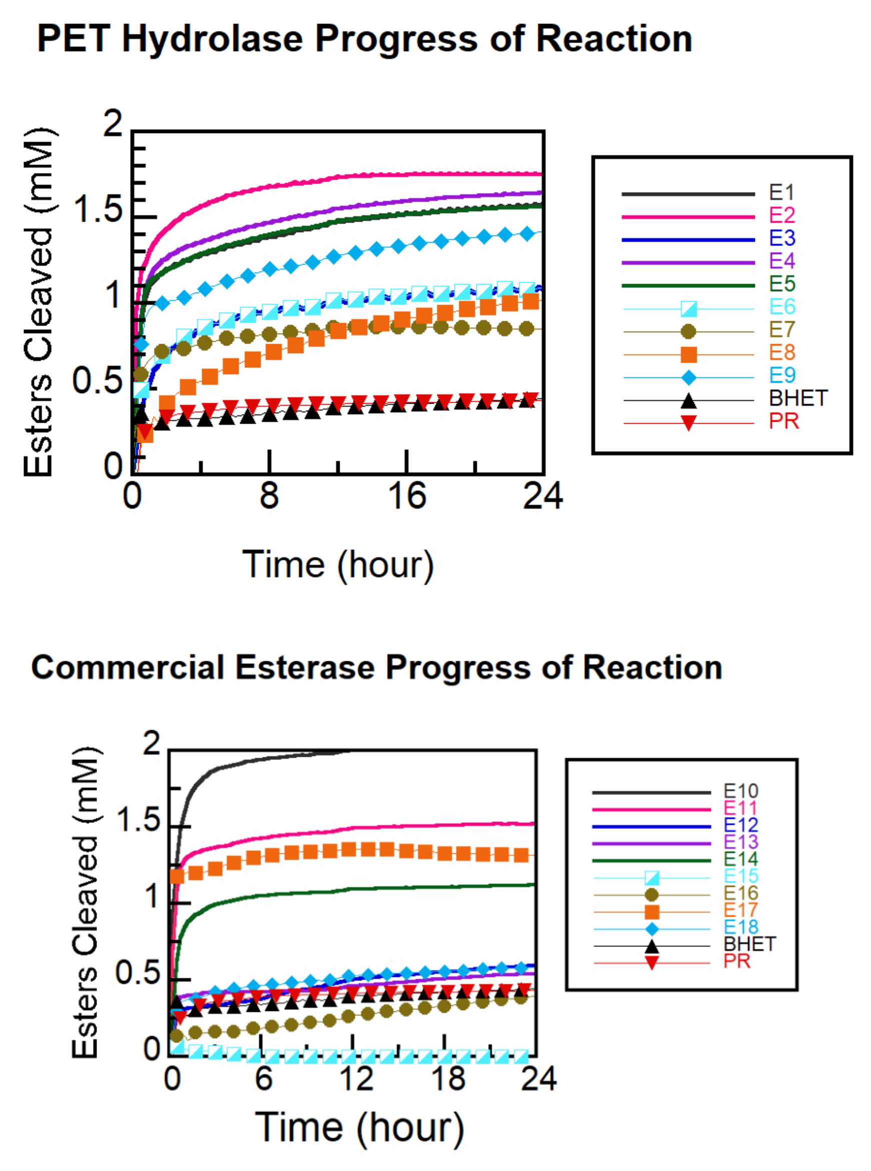

Figure S10. Enzymatic reactions catalyzed by the esterase set at 37 ºC and pH 8 (see Figures 3-4) were monitored colorimetrically over time. Absorbance at 550 nm (absorbance maximum for the alkaline form of PR) was recorded every 15 min. In both plots the solid black line indicates a control reaction where no enzyme was added to the well (“BHET”). The phenol red indicator is also shown (red triangles, “PR”) in the absence of BHET or enzyme.

Figure S11

```
#=======================================
#
# Aligned_sequences: 2
# 1: AAA81915.1
# 2: 6EQDA
# Matrix: EBLOSUM62
# Gap_penalty: 10.0
# Extend_penalty: 0.5
#
# Length: 547
# Identity:      59/547 (10.8%)
# Similarity:    97/547 (17.7%)
# Gaps:         307/547 (56.1%)
# Score: 18.5
#
#
#=======================================

pdb|6EQD|A      ------------------------------------------------------------     0
AAA81915.1      MTHQIVTTQYGKVKGTTENGVHKWKGIPYAKPPVGQWRFKAPEPPEVWEDVLDATAYGPI     60


pdb|6EQD|A      ------------MNFPRASRL-----------------MQAAVLGGLMAVSAAATAQTNP     31
AAA81915.1      CPQPSDLLSLSYTELPRQSEDCLYVNVFAPDTPSQNLPVMVWIHGGAFYLGAG----SEP     116
                             ::** *.                  : . : ** : :.*.    ::*

pdb|6EQD|A      YARGPN------PTAASLEASAGPFTVRSFTVSRPSGYGAGTVYYPTNAGGTVGAIAIVP     85
AAA81915.1      LYDGSKLAAQGEVIVVTLNYRLGPFGFLHLSS-----FDE---AYSD-----------NL     157
                   * :        ..:*:   *** .  ::      :.     *

pdb|6EQD|A      GYTARQSSIKWWGPRLASHGFVVITIDTNSTLDQPSSRSSQQMAALRQVASLNGTSSSPI     145
AAA81915.1      GLLDQAAALKWVRENISAFGGDPDNVT---VFG--ESAGGMSIAALLAMPAAKGLFQKAI     212
                *   : :::**   .:::.*    .:    .:.  .* .. .:***  : : :*  .. *

pdb|6EQD|A      YG-------------KVDTARMGVMGWSMGGGGSLISAANNPSLKAAAPQAPWDSSTNFS     192
AAA81915.1      MESGASRTMTKEQAASTAAAFLQVLGINESQLDRLHTVAAEDLLKAADQLRIAEKENIFQ     272
                               .. :* : *:* . .  . * :.* :  ****      :... *.

pdb|6EQD|A      SV----TVPTLIFACENDSIAPVNSSALPIYDSMSRNAK-QFLEINGGSHSCANSGNSNQ     247
AAA81915.1      LFFQPALDPKTLPEEPEKSIAEGAASGIPLLIGTTRDEGYLFFTPDSDVHSQETLDAALE     332
                 .      *. :    :.***   :*.:*:  . :*:    *:  :.. **  . . : :

pdb|6EQD|A      ALIG---------------------------------------KKGVAWMKRFMDNDTR-     267
AAA81915.1      YLLGKPLAEKAADLYPRSLESQIHMMTDLLFWRPAVAYASAQSHYAPVWMYRFDWHPEKP     392
                 *:*                                       : . .** **  :  :

pdb|6EQD|A      -Y------------------------------------------STFACENPNSTRVSDF     284
AAA81915.1      PYNKAFHALELPFVFGNLDGLERMAKAEITDEVKQLSHTIQSAWITFAKTGNPSTEAVNW     452
                 *                                           ***  .  **.. ::

pdb|6EQD|A      RTANCSLEHHHHH-----------------H------     298
AAA81915.1      PAYHEETRETVILDSEITIENDPESEKRQKLFPSKGE     489
                 : : . ..
```

Figure S11 Sequence alignment of *B. subtilis* carboxylesterase (Accession AAA81915.1) (enzyme 11) and *I. sakaiensis* PETase (6EQD) (enzyme 2) using Needle align.[3] The two sequences share 11% identity and 18% similarity.

Figure S12

```
#=======================================
#
# Aligned_sequences: 2
# 1: EMBOSS_001
# 2: EMBOSS_002
# Matrix: EBLOSUM62
# Gap_penalty: 10.0
# Extend_penalty: 0.5
#
# Length: 386
# Identity:      41/386 (10.6%)
# Similarity:    71/386 (18.4%)
# Gaps:         227/386 (58.8%)
# Score: 18.5
#
#
#=======================================
```

```
OAO77298.1         -----------------MMKI-----VPPKPFFFEAGERAVLLLHGFTGNSADVRMLGRF     38
pdb|6EQD|A         MNFPRASRLMQAAVLGGLMAVSAAATAQTNPYARGPNPTAAS-LEASAG-PFTVRSF-TV     57
                                    :* :     .  :*:    .  *.  *.. :*    ** :  .

OAO77298.1         LESKGYTCHAPIYK---G-----HGVPPEELVHTGPDDWWQDVMNGYEFLKNKGYEKIAV     90
pdb|6EQD|A         SRPSGYGAGTVYYPTNAGGTVGAIAIVPGYTARQSSIKWWGPRLASHGFVVITIDTNSTL     117
                    . .** . :  *    *      .: *   .: .  .**   : .: *:  .   : ::

OAO77298.1         AG---------LSLGGVFSLKLGYTVPIEGIVTMCAPMYIKSEETMYEGVLEYAREYKKR     141
pdb|6EQD|A         DQPSSRSSQQMAALRQVASLNGTSS----------SPIYGKVD-TARMGVMGWSMG----     162
                               :*  * **:   :          :*:* * : *   **: ::

OAO77298.1         EGKSEEQIEQEMEKFKQTPMKTLKAM-QELIADVRDHLDLIYAPTFVVQARHDEMINPD-     199
pdb|6EQD|A         GGGS--LISA-----ANNP--SLKAAAPQAPWDSSTNFSSVTVPTLIFACENDSIAPVNS     213
                    * *   *.       :.*  :***   :   *   ::. : .**::. ..:*.:   :

OAO77298.1         SANIIYNEIESPVKQIKWYEQSGHVITLDQEKDQLHEDIYAFLESLDW------------     247
pdb|6EQD|A         SALPIYDSMSRNAKQFLEINGGSHSCANSGNSN----QALIGKKGVAWMKRFMDNDTRYS     269
                   **  **:.:.  .**:   :  ..*  : . :.:    :     :.: *

OAO77298.1         -----------------------------     247
pdb|6EQD|A         TFACENPNSTRVSDFRTANCSLEHHHHHH     298
```

Figure S12 Sequence alignment of *G. stearothermophilus* carboxylesterase (Accession OAO77298.1) (enzyme 10) and *I. sakaiensis* PETase (6EQD) (enzyme 2) using Needle align.[3] The two sequences share 11% identity and 18% similarity.

Table S1. Esterases with previously established activity against solid phase PET used in this study

	Enzyme names	Accession number	PBD ID	Species of origin	MW (kDa)	References
1	TfH; TfCut2	WP_011291330.1	4CG1	*Thermobifida fusca* DSM4379 3	28.3	Kleeberg et al. (2005) [4]
2	PETase	GAP38373.1	*6EQE*	*Ideonella sakaiensis* 201-F6	30.2	Yoshida et al. (2016).[5]
3	Leaf-branch compost cutinase	AEV21261.1	4EB0	Uncultured bacterium	28	Sulaiman et al. (2012)[6]
4	TfAXE	ADM47605.1		*Thermobifida fusca* NTU22	29	Huang et al. (2010).[7]
5	Thf42_Cut1	ADV92528.1		*Thermobifida fusca* DSM4434 2	28.5	Herrero Acero et al. (2011)[8] Yang et al. (2007). [9]
6	Thc_cut2; Tfu_0882; cut_1	ADV92527.1	*5LUJ*	*Thermobifida cellulosilytica* DSM4453 5	29	Herrero Acero et al. (2011). Yang et al. (2007).[9]
7	Tha_Cut1	ADV92525.1		*Thermobifida alba* DSM4318 5	28.5	Ribitsch et al.(2012). [10]
8	est1	BAI99230.2		*Thermobifida alba*	29	Thumarat et al. (2015). [11] Hu et al. (2010).[12]
9	Thc_cut1; Tfu_0883; BTA1	ALF04778.1	*5ZOA*	*Thermobifida fusca*	28.5	Herrero Acero et al. (2011). [8] Yang et al. (2007).[9]

Table S2. Esterases obtained commercially (Sigma) and used in this study

Enzyme number	Enzyme name	E.C. number	Catalog number	Species of origin	MW (kDa)
10	esterase	3.1.1.1	79302-10MG	*Geobacillus stearothermophilus*	54
11	carboxylesterase	3.1.1.1	96667-10MG	*Bacillus subtilis*	32
12	lipase	3.1.1.3	62285-100MG-F	*Aspergillus oryzae*	41
13	lipase A	3.1.1.3	62287-50MG-F	*Candida antarctica*	45
14	lipase B	3.1.1.3	62288-50MG-F	*Candida antarctica*	56
15	lipase	3.1.1.3	79208-100mg-F	*Rhizopus oryzae*	125
16	phospholipase A1	3.1.1.32	L3295-50ML	*Aspergillus oryzae*	32
17	lipoprotein lipase	3.1.1.34	62335-10MG	*Pseudomonas sp.*	30
18	phospholipase A2	3.1.1.4	P8913-5MG	*bovine pancreas*	16

Table S3. Specific activity measured in units of micromoles esters hydrolyzed per minute per micromole enzyme for the first hour of reactions while the reaction rates exhibited a linear trend.

Enzyme Number	Enzyme Name	Specific Activity (µmol *p*NP ester cleaved min^{-1} µmol $enzyme^{-1}$)	Specific Activity (µmol BHET ester cleaved min^{-1} µmol $enzyme^{-1}$)
1	TfH; TfCut2	5910 ±0.10	2.12 ±0.0001
2	PETase	4710 ±0.0001	0.86 ±0.0001
3	LCC	3780 ±0.058	1.59 ±0.0001
4	TfAXE	7170 ±0.15	2.17 ±0.0001
5	Thf42_Cut1	8950 ±0.058	2.18 ±0.0001
6	Thc_cut2; Tfu_0882; cut_1	7640 ±0.059	0.92 ±0.0001
7	Tha_Cut1	26 ±0.012	0.81 ±0.0003
8	est1	6030 ±0.01	0.8 ±0.0030
9	Thc_cut1; Tfu_0883; BTA1	10500 ±0.059	2.46 ±0.0010
10	*Bacillus stearothermophilis* esterase	564 ±0.058	1.77 ±0.0004
11	*Bacillus subtilis* carboxylesterase	804 ±0.059	1.77 ±0.0003
12	*Aspergilis oryzae* lipase	3380 ±0.20	0.54 ±0.0002
13	*Candida antarctica* lipase A	866 ±0.059	0.51 ±0.00004
14	*Candida antarctica* lipase B	2740 ±0.058	1.64 ±0.00008
15	*Rhizopus oryzae* lipase	4540 ±0.059	0 ±0.0002
16	*Aspergilis oryzae* phospholipase A1	1110 ±0.057	1.66 ±0.0009
17	*Pseudomonas sp.* lipoprotein lipase	6720 ±0.87	1.66 ±0.0002
18	Bovine pancreas phospholipase A2	1890 ±0.012	0.81 ±0.0002

Supplementary Material References

1. Lundblad, R. L.; MacDonald, F. M., *Handbook of Biochemistry and Molecular Biology, Fourth Edition*. CRC Press: Boca Raton, FL, 2010; Vol. 4, p 1086.
2. R.W., S., *Handbook of Acid-Base Indicators* CRC Press: Boca Raton, FL, 2007.
3. Needleman, S. B.; Wunsch, C. D., A General Method Applicable to the Search for Similarities in the Amino Acid Sequence of Two Proteins. *Journal of Molecular Biology* **1970,** *48*, 443-453.
4. Kleeberg, I.; Welzel, K.; VandenHeuvel, J.; ller, R.-J. M.; Deckwer, W.-D., Characterization of a New Extracellular Hydrolase from Thermobifida fusca Degrading Aliphatic-Aromatic Copolyesters. *Biomacromolecules* **2005,** *6*, 262-270.
5. Yoshida, S.; Hiraga, K.; Takehana, T.; Taniguchi, I.; Yamaji, H.; Maeda, Y.; Toyohara, K.; Miyamoto, K.; Kimura, Y.; Oda, K., A bacterium that degrades and assimilates poly(ethylene terephthalate). *Science* **2016,** *351* (6278), 1196-1199.
6. Sulaiman, S.; Yamato, S.; Kanaya, E.; Kim, J. J.; Koga, Y.; Takano, K.; Kanaya, S., Isolation of a novel cutinase homolog with polyethylene terephthalate-degrading activity from leaf-branch compost by using a metagenomic approach. *Applied Environmental Microbiology* **2012,** *78* (5), 1556-62.
7. Huang, Y. C.; Chen, G. H.; Chen, Y. F.; Chen, W. L.; Yang, C. H., Heterologous expression of thermostable acetylxylan esterase gene from Thermobifida fusca and its synergistic action with xylanase for the production of xylooligosaccharides. *Biochemical and Biophysical Research Communications* **2010,** *400* (4), 718-23.
8. Herrero Acero, E.; Ribitsch, D.; Steinkellner, G.; Gruber, K.; Greimel, K.; Eiteljoerg, I.; Trotscha, E.; Wei, R.; Zimmermann, W.; Zinn, M.; Cavaco-Paulo, A.; Freddi, G.; Schwab, H.; Guebitz, G., Enzymatic Surface Hydrolysis of PET: Effect of Structural Diversity on Kinetic Properties of Cutinases from Thermobifida. *Macromolecules* **2011,** *44* (12), 4632-4640.
9. Yang, Y.; Malten, M.; Grote, A.; Jahn, D.; Deckwer, W. D., Codon optimized Thermobifida fusca hydrolase secreted by Bacillus megaterium. *Biotechnology Bioengineering* **2007,** *96* (4), 780-94.
10. Ribitsch, D.; Heumann, S.; Trotscha, E.; Herrero Acero, E.; Greimel, K.; Leber, R.; Birner-Gruenberger, R.; Deller, S.; Eiteljoerg, I.; Remler, P.; Weber, T.; Siegert, P.; Maurer, K. H.; Donelli, I.; Freddi, G.; Schwab, H.; Guebitz, G. M., Hydrolysis of polyethyleneterephthalate by p-nitrobenzylesterase from Bacillus subtilis. *Biotechnology Progress* **2011,** *27* (4), 951-60.
11. Thumarat, U.; Kawabata, T.; Nakajima, M.; Nakajima, H.; Sugiyama, A.; Yazaki, K.; Tada, T.; Waku, T.; Tanaka, N.; Kawai, F., Comparison of genetic structures and biochemical properties of tandem cutinase-type polyesterases from Thermobifida alba AHK119. *Journal of Bioscience Bioengineering* **2015,** *120* (5), 491-7.
12. Hu, X.; Thumarat, U.; Zhang, X.; Tang, M.; Kawai, F., Diversity of polyester-degrading bacteria in compost and molecular analysis of a thermoactive esterase from Thermobifida alba AHK119. *Applied Microbiology Biotechnology* **2010,** *87* (2), 771-9.

1. R. L. Lundblad and F. M. MacDonald, *Handbook of Biochemistry and Molecular Biology, Fourth Edition*, CRC Press, Boca Raton, FL, 2010.
2. S. R.W., *Handbook of Acid-Base Indicators* CRC Press, Boca Raton, FL, 2007.
3. S. B. Needleman and C. D. Wunsch, *Journal of Molecular Biology*, 1970, **48**, 443-453.
4. I. Kleeberg, K. Welzel, J. VandenHeuvel, R.-J. M. ller and W.-D. Deckwer, *Biomacromolecules*, 2005, **6**, 262-270.
5. S. Yoshida, K. Hiraga, T. Takehana, I. Taniguchi, H. Yamaji, Y. Maeda, K. Toyohara, K. Miyamoto, Y. Kimura and K. Oda, *Science*, 2016, **351**, 1196-1199.
6. S. Sulaiman, S. Yamato, E. Kanaya, J. J. Kim, Y. Koga, K. Takano and S. Kanaya, *Applied Environmental Microbiology*, 2012, **78**, 1556-1562.
7. Y. C. Huang, G. H. Chen, Y. F. Chen, W. L. Chen and C. H. Yang, *Biochemical and Biophysical Research Communications*, 2010, **400**, 718-723.
8. E. Herrero Acero, D. Ribitsch, G. Steinkellner, K. Gruber, K. Greimel, I. Eiteljoerg, E. Trotscha, R. Wei, W. Zimmermann, M. Zinn, A. Cavaco-Paulo, G. Freddi, H. Schwab and G. Guebitz, *Macromolecules*, 2011, **44**, 4632-4640.
9. Y. Yang, M. Malten, A. Grote, D. Jahn and W. D. Deckwer, *Biotechnology Bioengineering*, 2007, **96**, 780-794.
10. D. Ribitsch, S. Heumann, E. Trotscha, E. Herrero Acero, K. Greimel, R. Leber, R. Birner-Gruenberger, S. Deller, I. Eiteljoerg, P. Remler, T. Weber, P. Siegert, K. H. Maurer, I. Donelli, G. Freddi, H. Schwab and G. M. Guebitz, *Biotechnology Progress*, 2011, **27**, 951-960.
11. U. Thumarat, T. Kawabata, M. Nakajima, H. Nakajima, A. Sugiyama, K. Yazaki, T. Tada, T. Waku, N. Tanaka and F. Kawai, *Journal of Bioscience Bioengineering*, 2015, **120**, 491-497.
12. X. Hu, U. Thumarat, X. Zhang, M. Tang and F. Kawai, *Applied Microbiology Biotechnology*, 2010, **87**, 771-779.
